# The evolution of gene regulatory networks controlling *Arabidopsis thaliana* L. trichome development

**DOI:** 10.1186/s12870-019-1640-2

**Published:** 2019-02-15

**Authors:** Alexey V. Doroshkov, Dmitrii K. Konstantinov, Dmitrij A. Afonnikov, Konstantin V. Gunbin

**Affiliations:** 1 The Siberian Branch of the Russian Academy of Sciences (IC&G SB RAS), The Institute of Cytology and Genetics, Novosibirsk, Russia; 2 Novosibirsk State University (NSU), Novosibirsk, Russia; 3 School of Life Science, Immanuel Kant Federal Baltic University, Kaliningrad, Russia; 4 Center of Brain Neurobiology and Neurogenetics, Institute of Cytology and Genetics SB RAS, Novosibirsk, Russia

**Keywords:** Leaf epidermis, Gene regulatory network, Protein evolution, Combinatorial gene regulation, Trichome

## Abstract

**Background:**

The variation in structure and function of gene regulatory networks (GRNs) participating in organisms development is a key for understanding species-specific evolutionary strategies. Even the tiniest modification of developmental GRN might result in a substantial change of a complex morphogenetic pattern. Great variety of trichomes and their accessibility makes them a useful model for studying the molecular processes of cell fate determination, cell cycle control and cellular morphogenesis. Nowadays, a large number of genes regulating the morphogenesis of *A. thaliana* trichomes are described. Here we aimed at a study the evolution of the GRN defining the trichome formation, and evaluation its importance in other developmental processes.

**Results:**

In study of the evolution of trichomes formation GRN we combined classical phylogenetic analysis with information on the GRN topology and composition in major plants taxa. This approach allowed us to estimate both times of evolutionary emergence of the GRN components which are mainly proteins, and the relative rate of their molecular evolution. Various simplifications of protein structure (based on the position of amino acid residues in protein globula, secondary structure type, and structural disorder) allowed us to demonstrate the evolutionary associations between changes in protein globules and speciations/duplications events. We discussed their potential involvement in protein-protein interactions and GRN function.

**Conclusions:**

We hypothesize that the divergence and/or the specialization of the trichome-forming GRN is linked to the emergence of plant taxa. Information about the structural targets of the protein evolution in the GRN may predict switching points in gene networks functioning in course of evolution. We also propose a list of candidate genes responsible for the development of trichomes in a wide range of plant species.

**Electronic supplementary material:**

The online version of this article (10.1186/s12870-019-1640-2) contains supplementary material, which is available to authorized users.

## Background

To understand the processes of development and evolution of living organisms, the “gene regulatory networks”, or GRNs have to be taken into account. The variability of such networks determines the diversity of organ forms and functions in plants and animals [1, 2]. Specialized trichome cells are useful as a model for studying the molecular processes of cell fate determination, cell cycle control and cellular morphogenesis [3]. In particular, this model was instrumental in dissecting the mechanisms of epidermal morphogenesis in the model plant *Arabidopsis thaliana L* [4]. The central role in determining the cellular fate of cells with trichomes is played by the assembly of the trichome initiation MBW complex - (GL3/EGL3-GL1-TTG1), which initiates the expression of the gene GLABRA2 (GL2) encoding a transcription factor to initiate the cell transition to differentiation into trichomes [5]. In addition to GL2, the MBW complex induces the expression of repressor genes (TRY/CPC), which can move between the cells and assemble into a complex (GL3/EGL3-CPC/TRY-TTG1) that is unable to initiate trichome formation. In *Arabidopsis*, seven R3-MYB proteins of inhibitors of the MBW complex were found: TRIPTYCHON (TRY) [6, 7], CAPRICE (CPC) [8], ENHANCER OF TRY and CPC 1, 2 и 3 (ETC1, ETC2 и ETC3) [9–11], and TRICHOMELESS 1 и 2 (TCL1 2 TCL2) [12, 13]. Different efficiency of the function between them was shown [11, 14]. TCL1 most likely acts as a negative regulator of GL1 expression [12] as well as trichome development, influencing both the expression of GL1 and competing with GL1 for binding to GL3 [15, 16]. It is to be noted that the pattern of trichome formation is described by the widespread mechanism of lateral inhibition, which is known to exist in various plant and animal organisms. In addition, it is responsible for the cyanobacterial heterocyst development [17, 18].

In addition to the MBW complex, a number of genes that increase the expression of the genes of the initiator complex, were found in leaves and flowers: GLABROUS INFLORESCENCE STEMS (GIS), [19] GIS2, ZINC FINGER PROTEIN 8 (ZFP8) [20, 21], ZFP5 [22, 23], и ZFP6 [24]. It was shown that GL1 and GL3, which are the key transcription factors in the MBW complex, function after being activated by GIS2 and ZFP8 [24].

A number of studies have shown that genes orthologous to the *Arabidopsis* trichome related genes are involved in the cotton hair formation [25–29]. However, it was earlier suggested that in more phylogenetically distant species trichomes can develop in a convergent way through other genetic mechanisms [30]. Also, certain data speak in favor of functional diversification of individual regulatory pathways of trichome development. It was shown that the ectopic expression of the rice R3 MYB transcription factor OsTCL1 in the *Arabidopsis* genome influences the trichome formation; however, changes in OsTCL1 expression in rice do not lead to any trichome-related phenotypic changes [31]. In addition, the overexpression of the GL1 gene in tobacco has no effect on the development of trichomes. One explanation is that the gene network with the GL1 gene first appeared in Rosids. Besides, tobacco has five types of trichomes, which should reflect no differences in genetic mechanisms, either [30].

It should be noted that the MBW complex, together with its regulators, directly participates in inhibition of morphogenesis of root hairs [7, 9, 10, 32]. Thus, there is reason to suggest that variations of one gene network are responsible for formation of the trichome pattern of leaf epidermis and root hairs in *A. thaliana* [33]. Using RNA-seq data, Huang showed that the main set of genes responsible for root hairs is preserved at evolutionary distances up to 200 million years or more [34]. However, the patterns of expression of these genes can vary significantly between different species [35].

It is also known that outgrowths of epidermal cells are widespread and are extremely ancient formations. Simple outgrowths are found in algae - *Chara* (*Charophytales*) and *Spirogyra* (*Zygnematales*) [36]. Risoids in mosses have a characteristic pattern and perform the functions of fixation in the substrate involved in absorption of water and nutrients [37]. It was revealed that *Physcomitrella patens* genes PpRSL1 and PpRSL2 affect the number of rhizoids on a plant [38, 39]. Mutants of *Arabidopsis* devoid of the function of RHD6 (one of the key genes of hair development) develop root hairs if they are transformed by the genes PpRSL1 from *Physcomitrella*. This indicates that the function of the RSL family proteins has not been lost for 420 million years of the species divergence [38].

Thus, to understand the processes of development and evolution of trichome morphogenesis of GRN, we need to combine data on proteins and their functions into the GRN topologies related to each major plant taxa divergence, and after we need to associate the changes in the GRN topologies with the changes in the GRN components (individual proteins).

Functions of any protein are a direct consequence of its chemical and physical properties, which in turn are defined by sterical and physico-chemical requirements for native folding in three-dimensional space into the protein globule. Therefore, it is anticipated that the change of residue interacting with other amino acids in a protein globule, is closely related to changes in the context of epistatic interactions of residues in a globule. In other words, protein evolution is rugged, and unevenness is driven by abrupt changes in the optimal three-dimensional protein space topology (e.g. Gibbs energy), which in turn leads to rugged selection in protein space and evolutionary time. Computational studies of protein evolution detected several well-known major epistasis signatures. These are (1) variability in amino acid states that cause protein malfunctions (or diseases) in various lineages [40]; (2) mutation tolerability switching along protein evolution, or, in other words, deleterious mutations at one evolutionary time becoming non-deleterious or vice versa [41]; (3) pervasive signatures of covariation in any proteins and any lineages [42–44]. In addition, gradual emergence of restrictive epistatic interactions was demonstrated to take place in the course of protein evolution [45, 46]. These interactions in turn makes the ancestral state deleterious or irreversible [45] or ‘Stokes shifts’ in protein evolution [46]. Despite these facts, until now the vast majority of currently available reconstruction procedures of ancestral sequences [47, 48] are based on reversibility of a single empirical amino acid substitution matrix (that is applied to all protein sites. Thus, the novel ancestral protein reconstruction software tools (e.g. ProtASR) [49] that adapt the protein structure and the folding stability should be most suitable. However, there is still a lack of experimentally solved 3D protein structures, notably in the plant science. Another way to account for protein epistasis in the standard ancestral protein reconstruction is construction of ancestral libraries to address the sequence uncertainty as a result of ancestral sequence reconstruction imperfection [50]. This approach takes into consideration a well-known pitfall that there is no guarantee that the ancestral sequences are correct biologically functional proteins and most useful in studying deep evolutionary events. The recent experimental study of mRFP1 protein artificial evolution shows that the ancestral sequences obtained by the maximum likelihood approach is most closely related to natural ancestral mRFP1 proteins, while the best proteins reconstructed by using the phylogenetic-tree-aware Bayesian method are not so similar to native ancestors [51]. However, only one best ancestral protein can be reconstructed using this approach that cannot be used in ancestral libraries generation. In order to make ancestral libraries generation sufficiently accurate, it was recently suggested using the ‘AltAll’ reconstruction approach. This approach combines all plausible alternative states introduced into a single protein and then functionally characterizes this protein by the set of these states [52, 53]. It was shown that this approach significantly corrects imperfection of ancestral sequences generated by Bayesian posterior probability exploration. Thus, the best we could do in the case of the lack of 3D protein structures was to use the ‘AltAll’ derived approach to construct ancestral libraries for subsequent evolutionary studies and to make evolutionary protein function inferences.

Thus, two general objectives are highly relevant to our study: (1) to fill the gaps in understanding of evolutionary dynamics of the trichome morphogenesis GRN topology, we need to combine taxa-specific GRN and analyze their differences and (2) to fill the gaps in understanding the molecular basis of protein interactions into the taxa-specific GRN and the molecular basis on differences between the taxa-specific GRN, the evolution of structure and function of GRN proteins should be analyzed. In this work, we combine the qualitative information on the topology of GRN related to trichome morphogenesis with in-detail phylogenetic analysis of its components. The raw phylogenetic analysis allowed us to find a simple answer when the origination point of the core gene subnetwork is formed. Additionally, using detailed information about protein sequence structural classes/features, we studied the evolutionary variation of protein globules related to various speciation or duplication points and potential protein-protein interactions. This allows to hypothesize divergence and/or specialization in the GRN function associated with origination of plant taxa. Information about the structural targets of the protein evolution in the GRN also plays a predictive role for future discriminations of evolutionary switching points in the functioning of gene networks.

## Results and discussion

### GRN reconstruction

Based on multiple expert analysis of the functional annotation, a list containing 90 genes associated with formation of the trichome *A. thaliana* was created*.* In the process of enrichment, we used manual analysis of articles and information from the STRING database and the Cytoscape (GeneMania plugin) system. As a result, genes with the highest score of connection with our gene sample were added. The resulting enriched gene network contained 123 nodes.

Among nodes, 51 transcription factors and 6 genes of the cell cycle were detected (Additional file 1). In the sample set 109 Superfamily domains were revealed (Additional file 1). Among them, MYB, SANT, Homeobox, C2H2 should be noted - these domains are characteristic for proteins, whose functions include the regulation of transcription, In addition, we found proteins that contain the HLH-domain, which mediates protein-protein interactions. The gene network was then analyzed by co-occurrence of the GO-terms (For details see Additional file 1).

**Fig. 1:**
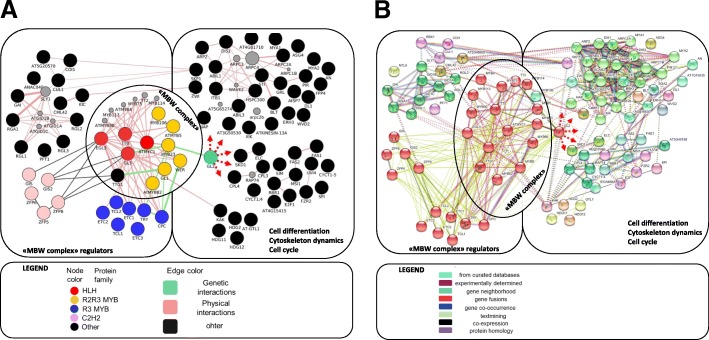
GRN related to trichome development reconstructed using Cytoscape GeneMANIA plugin (**a**) and same network enriched with edges from STRING DB (**b**). Node and edge color schemes were shown under each network

Genes that do not interact with other genes and do not have experimentally confirmed information about direct participation in initiation and/or development of trichomes have been removed from the network. Statistics of this network before and after verification and reduction is provided in Additional file 2. The graphic representation of the gene network by means of STRING and Cytoscape data is shown in Fig. 1. In this network, an area associated with a large number of protein-protein interactions corresponding to the components of the MBW complex (11 nodes, marked in Fig. 1), as well as its 7 inhibitors - TRY, CPC, ETC1, ETC2, ETC3, TCL1, and TCL2, were shown. In the left part of the network, a number of regulators of the expression of MBW complex components are represented: these are 21 genes, some of which show the expression of the genes of the trichome initiator complex, its direct regulation or participation in the transmission of the hormonal signal (6 of which are sensitive to GA and cytokinins, 6 are sensitive to jasmonic acid, see Additional file 1. In the right part of the network there are regulatory cascades, whose work is presumably under control of the initiator complex, this part of the network contains genes associated with the growth and differentiation of the trichome cells. They regulate such processes as cell differentiation, cytoskeleton dynamics and cell cycle.

For the vast majority of genes, e.g. SPI, SIM, FAS, KAK, STI, direct participation in the regulation of the processes of cell differentiation of trichomes, formation of branches, and control of endoreduction of cells in trichomes is shown (Additional file 1). We marked the messenger GL2 in Fig. 1, because it is the key protein in the selection of the cellular fate of the trichoblast.

### Phylogenetic analysis of MBW trichome initiation complex components

**Fig. 2:**
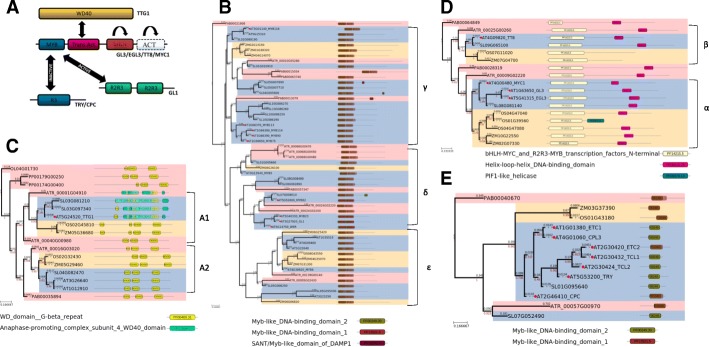
Scheme of MBW complex assembly (**a**) and reduced phylogenetic trees of its components: Gl1 (**b**), Gl3/EGL3/MYC1/TT8 (**c**), TTG1 (**d**) and TRY/CPC/ETC1,2/TCL1,2/CPL3 (**e**). Full tree topology given in supplementary Figs. S3-S6

The MBW complex contains 11 proteins, and their function is partially repeated. These proteins belong to four families: HLH (4 proteins: TT8, EGL3, GL3, MYC1), MYB-R2R3 (5 proteins: GL1, WER, MYB82, MYB5, MYB23) and WD40 (1 representative -TTG1). The MBW complex assembly scheme and the reduced phylogenetic trees of the nearest homologues are shown in Fig. 2. The complete trees are given in (Additional files 3, 4, 5 and 6: Figures S1A-S3A). Data on changes in protein structures also given in corresponding (Additional files 3, 4, 5 and 6: Figures S1B-S3B ).

The GL3 (AT1G63650) and EGL3 (AT5G41315) sequences are clustered together. Their divergence occurred in the common ancestor of *Brassicaceae*. The MYC/MYB N-terminal domain of transcription factors (IPR025610 (PF00010)) is about 180 amino acids long. It was predicted for all the homologous sequences of dicotyledons comprising clade α1 (in Fig. 2b and Additional file 3), whereas the basic helix-loop-helix domain (bHLH, IPR011598 (PF00010)) with the length of about 45 amino acids, mediates protein dimerization and characterizes protein transcription factors. Diversification of the evolutionary lineages of Gl3/EGL3 and MYC1 occurred in the common ancestor of dicotyledonous species before the divergence of the main evolutionary lineages of dicotyledonous plants. It should be noted that the α2 clade sequences have the bHLH domain in a reliably predicted state only in a part of the sequences, whereas in the outer group of monocots, both domains are strongly predicted. The protein sequences orthologous to TT8 in dicotyledonous and monocotyledonous plants form a separate clade β and have the same domain composition. Separation of these lineages occurred before the divergence of dicotyledonous and monocotyledonous plants. It should be noted that the homologous sequences in mosses and gymnosperms in the nearest outgroup reveal the bHLH and MYC/MYB N-terminal domains (Fig. 2b and (Additional file 3: S1A). In addition to Gl3 and EGL3, there are indications that close genes (TT8 AT4G09820 and MYC1 AT4G00480) also affect trichome development [54–56]. Together with the conservative domain organization, this suggests that participation in the assembly of the complex regulating morphogenesis is a primary function and could have appeared in early land plants.

As a result of analysis of the evolutionary changes in protein structures (see Methods), it was found that the largest changes occurred on the long basal branch of the homologues EGL3 and GL3 of *Brassicaceae* sequences before their separation from each other (Additional file 3: Figure S1B ). The same was observed for the basal branch of the *Brassicaceae* MYC1 clade. However, the common ancestor of the huge clade of flowering plants containing EGL3, GL3 and MYC1 is characterized by low protein structure variability; the same can be said about the other studied taxa of dicotyledonous plants (*Carica papaya; Fragaria vesca; Manihot esculenta; Populus trichocarpa; Prunus persica*). Significant changes in the structure of the EGL3/GL3 protein are observed on the basal branch of monocotyledonous plants, as well as in the divergence of dicotyledons and monocotyledons and in the divergence within monocotyledons. Similarly, significant changes are observed in the common ancestor of Solanaceae, but this is probably due to the fact that they diverged very early in the evolution.

The TTG1 protein (AT5G24520) of *A. thaliana* participates in assembling the MBW complex [57–59] Sequences orthologous to TTG1 form a clade α (Fig. 2c and (Additional file 4: S2A)) observed in dicots and monocots. In all homologous sequences of plants belonging to the adjacent clade (α in (Additional file 4: S2)), WD40 blocks were predicted, which, according to [60], allows proteins to mediate the assembly of protein complexes. Together with the clade α, an additional clade β is clustered that includes the AT3G26640 (LWD2) and AT1G12910 (ATAN11) proteins, which are associated with the functioning of circadian rhythms. This suggests that the ancestor of dicotyledons and monocots experienced diversification of the ancestral WD40 into two evolutionary lineages that differed in their biological functions. Sequences of coniferous plants (PAB00042769, PAB00049457, PAB00035894, PAB00018188) and mosses (PP00290G00030, PP00092G00020, PP00179G00250, PP00174G00400) also have domain organization similar to TTG1 *A.thaliana*. In the ancestral evolutionary lineage of TTG1 sequences in monocotyledons, the structure of the protein undergoes essential changes (Additional file 4: Figure S2B). In the *Brassicaceae* clade, the results differ from each other. Protein disorder regions and secondary structure annotated using 3-state model show drastic changes. At the same time, the secondary structure described by 8-state model and the amino acid substitution rate (compared to the protein-specific amino acid replacement rate model) show relatively high conservatism of structures. There are also significant changes in Solanaceae, but this is probably due to the fact that they diverged very early in evolution. The other dicotyledons do not reveal any specific patterns of protein changes.

The Gl1 protein, an important protein for the MBW complex, belongs to the R2R3-MYB protein family. This family contains 126 proteins in *A. thaliana* and is divided into 25 subgroups depending on the C-terminal motive [61]. Representatives of the 15th subgroup R2R3 MYB (e.g. MYB0/GLABROUS1 (GL1), MYB23 [62], MYB5 [63], MYB82 [64], WER [65] are involved in morphogenesis of trichomes. These phylogenetic relationships are shown in (Additional file 5: Figure S3A) and 2D. All the proteins contain two MYB-DNA binding domains at the N-terminus. The phylogenetic relationships of proteins are weakly resolved, which is related to the specificity of the evolution of MYB factors and their small protein length. The divergence of MYB23, WER, GL1 occurred in the ancestor of *Brassicaceae*. The divergence of their lineage with the MYB82 lineage seems to have occurred in the ancestor of dicotyledons. For AT3G13540 (MYB5), orthologs are identified in both dicotyledonous and monocotyledonous plants. The same is observed for AT4G38620 - MYB4. However, together with MYB4, the genes AT4G09460 (MYB6) and AT1G22640 (MYB3) are clustered. These particular genes are not known as participating in the development of trichomes. Genes MYB113, MYB114, MYB90, MYB75, MYB116 are clustered together, they have been described as participating in the synthesis of anthocyanins [66, 67]. Thus, a wide spectrum of R2R3-MYB proteins of *Arabidopsis* is observed, and for some of them groups of orthologous sequences down to a common ancestor of flowering plants are found. In the R2R3-MYB homologous phylogenetic tree, there are three clades (MYB75/MYB90/MYB113/MYB114, WER/MYB23/GL1 and a clade containing no sequences of *Brassicaceae*). MYB75/MYB90/MYB113/MYB114 and the dicotyledonous proteins close to them changed their protein structure more strongly than the protein from clade WER/MYB23/GL1 (Additional file 5: Figure S3B). In the dicotyledonous group not containing *Brassicaceae* sequences, an accelerated change in secondary structures is also observed in comparison with the clade WER/MYB23/GL1. In the clade of monocots, relative conservatism of proteins is observed (in comparison with all the dicots). It should be noted that secondary structures are relatively well-preserved (compared to in-store ones) amongst the clades, while the tree branches are rather long, indicating a high rate of accumulation of amino acid substitutions.

Proteins of the R3-MYB family are presented in *Arabidopsis* as a series of 7 paralogical genes (AT2G46410 (CPC), AT1G01380 (ETC1), AT4G01060 (CPL3), AT2G30420 (ETC2), AT2G30432 (TCL1), AT2G30424 (TCL2), AT5G53200 (TRY). In other species of dicots, the number of homologues varies from 1 (strawberry) to 4–5 (EG and MD, respectively) (Additional file 6: Figures S4 and 2E) All the monocot genes are represented in 1 copy. All the R3-MYB proteins contain one MYB-DNA binding domain in the middle. In the phylogenetic tree of R3-MYB proteins, there are three clades. The first one includes monocotyledonous and dicotyledonous plants. The second one includes dicotyledonous plants and gymnosperms. We found an accelerated change in the secondary structure from the ancestor of flowering plants to monocotyledonous ones (with a change in the secondary structures within the monocotyledons, see (Additional file 6: Figure S4B)). In the second clade, a slowdown in the accumulation of substitutions is observed in both dicots and gymnosperms.

Phylogenetic analysis of the other nodes of the network is given in Additional files 7, 8, 9, 10, 11 and 12.

### Phylogenetic analysis of trichome initiation complex components

**Fig. 3:**
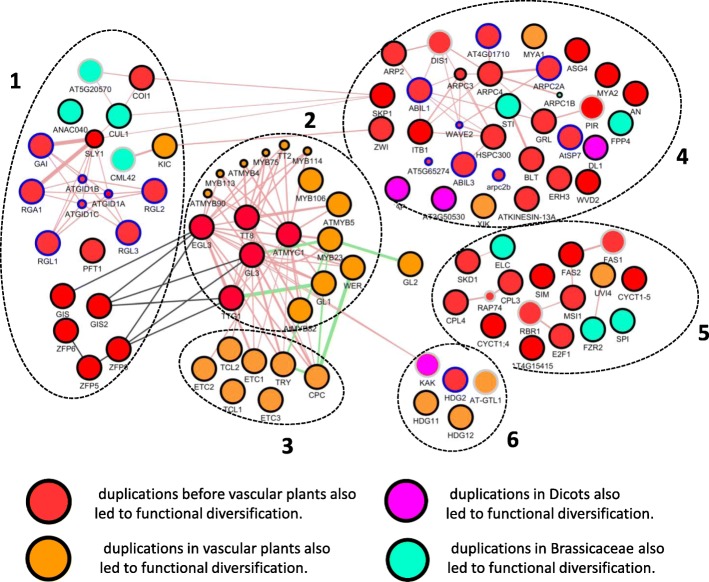
Estimated time of appearance of the function of each of the nodes of the network of epidermal morphogenesis *A. talyana.* The color scheme is given below. Clusters are distinguished according to functional similarity of nodes: 1 - Nodes related to hormonal signal transduction. This cluster contains hormone-responsive regulators and regulators responsible for the perception of the hormonal signal. 2 - A complex of MBW trichome initiation components. This cluster contains transcription factors and factors responsible for initiating cell development along the path of trichomes. 3 - A complex of MBW trichome initiation inhibitors. This cluster contains the R3MYB genes and is responsible for the inhibition of the initiator complex (see the introduction for details). 4 - Nodes related to the cytoskeleton structure and the functioning dynamics. This cluster contains genes involved and the regulating assembly of actin and myosin filaments in the cell. 5 - Nodes related to cell cycle dynamics. This cluster contains genes regulating the cell cycle, in particular G1/S transition of the mitotic cell cycle; the endomitotic cell cycle; and regulation of DNA endoreduplication. 6 - Specific trichome differentiation genes. This cluster contains genes for which participation in the development of trichomes has been shown, but their role is currently not fully understood

Large-scale analysis of evolution made it possible to estimate the time of appearance of the function of each of the nodes in the gene network. The most extensive orthological groups including *P.patens* proteins and those not containing early duplications correspond to 40 nodes of the gene network, which are shown on Fig. 3. For these proteins, we can assume earlier isolation of the function - at the level of the common ancestor of vascular plants.

These nodes are relatively evenly represented in different parts of the gene network and include 21 proteins associated with the functioning of the cytoskeleton, 12 proteins that mediate hormonal regulation, as well as 6 proteins not belonging to these groups (see Additional file 2). These proteins are marked as red node in the Fig. 3.

A number of proteins having basal duplication in the tree topology in the common ancestor of dicotyledonous plants or in cruciferous plants and has duplications in both daughter clades (see Additional file 2). These proteins are marked in blue ring in the Fig. 3.

A number of duplications in vascular plants led to functional diversification. For such proteins, we can assert the presence of a basal function corresponding to the gene network only at the level of the common ancestor of vascular plants. Genes associated with the cytoskeleton function (AT1G17580 (MYA1); AT5G20490 (XIK)), functioning of the cell cycle (AT2G42260 (UVI4)), involved in the transmission of hormonal signals (AT2G46600 (KIC); AT4G24210 (SLY1)) as well as three proteins with not fully established functions (see. Additional file 2). These proteins are marked as the orange node in Fig. 3.

A number of duplications in dicotyledons led to functional diversification. For such proteins, we can assert the presence of a function at the level of the common ancestor of dicotyledonous plants. These are 2 genes associated with the work of the cytoskeleton (AT5G42080 (DL1); AT3G50530), and two genes, the function of which has not yet been fully elucidated (AT1G69490; AT4G38600 (KAK)). For the KAK gene, duplication is also found in the ancestor of monocotyledonous plants. These proteins are marked as the pink node in Fig. 3.

A number of duplications in *Brassicaceae* also led to functional diversification. For such proteins, we can assert the presence of a function of interest at the level of the common ancestor of *Brassicaceae* or only in *Arabidopsis* species. The function of these proteins was considered as “young”. These nodes include 3 proteins associated with the functioning of the cytoskeleton **(**AT2G31300 (ARPC1B); AT1G19835), 2 proteins associated with cell cycle dynamics (AT4G22910 (FZR2); AT3G12400 (ELC)), 6 proteins that mediate hormonal regulation (AT4G20780 (CML42); AT5G20570 (RBX1); AT4G02570 (CUL1); AT2G27300 (ANAC040); AT5G20570 (RBX1)), as well as six proteins not belonging to these groups (AT2G02480(STI); AT1G03060(SPI)). These proteins are marked as the green node in Fig. 3.

Proteins that have relatively early duplications in monocotyledonous plants are noted. These are 5 genes associated with the functioning of the cytoskeleton (AT1G13180 (TMM); AT5G18410 (PIR121); AT3G12280 (RBR1); AT1G65470 (FAS1)), 2 genes of hormonal regulatory pathways (AT4G20780 (CML42); AT5G20570 (RBX1)), and six proteins not belonging to these groups (AT4G38600 (KAK); AT1G33240 (AT-GTL1); AT4G12610 (RAP74)). These proteins are marked in the gray ring in Fig. 3.

A number of genes do not fit into a simple classification and require a separate mention.

AT5G28646 WVD2 is a gene associated with the work of the cytoskeleton. The ancestor of dicotyledons had a duplication after divergence from monocots. The ancestor of the cereal plants has been identified as having two duplications.

Another gene associated with the work of the cytoskeleton, AT5G43900 MYA2, also has two duplications in the ancestor of the cruciferous plants and one in the ancestor of the cereal plants.

AT4G15415 associated with the cell cycle progression has two separate clades of dicotyledons and one clade of monocots, in which two duplications have occurred.

Divergence of cyclines (AT1G47870 (E2F2), AT5G22220 (E2F1), AT2G36010 (E2F3)) occurred in the ancestor of the flowering plants after separation of the amborella.

AT1G01520 (ASG4) and AT4G01280 (it is not a node of the network under study) diverged from the ancestor of dicotyledons. Paralog functions (AT4G01280) were not clarified. In monocots, duplication is also noted.

Evolutionary lineages AT5G06650 (GIS2), AT3G58070 (GIS) and AT2G41940 (ZFP8) diverged from the ancestor of flowering plants and had an outgroup PAB00021121. We identified two duplications in the clades of *Brassicaceae* and monocotyledons (one before the divergence from the banana and one after).

Evolutionary lineages of AT5G06650 (GIS2), AT3G58070 (GIS) and AT2G41940 (ZFP8) diverged from each other in the ancestor of the flowering plants (outgroup PAB00021121). We identified two duplications in the clades of *Brassicaceae* and monocots (one before the divergence from the banana and one after).

At the next stage, assessment of the quantitative composition of the gene network was made (Additional file 2). In most flowering plants carrying both trichomes and root hairs, the number of genes varies from 120 to 170. In the species that had recent whole-genome duplication (the last 10 MYA), there are more than 190 genes (*Brassica rapa*, *Glycine max*, *Gossypium raimondii*, *Malus domestica*, *Manihot esculenta*, *Populus trichocarpa*). In *Beta vulgaris*, we found a relatively small number of genes - 95. The well-described representative of gymnosperms, for which the presence of differentiation of the integumentary tissues and the presence of the root hairs were shown, *Picea abies* has 150 genes orthologous to the GRN nodes under study.

Representatives of lower plants carrying a variety of outgrowths have about 100 genes corresponding to the GRN nodes (*Amborella trichopoda* - 108; *Physcomitrella patens* - 119). Even more anatomically simple organisms - *Selaginella moellendorffii* and *Marchantia polymorpha* have 57 and 48 genes, respectively, and unicellular *Chlamydomonas reinhardtii Ostreococcus lucimarinus* - have 19 and 22 genes respectively.

For a more detailed assessment of the dependence of the quantitative composition of the GRN on the complexity of morphogenesis, the principal component analysis was applied.

**Fig. 4:**
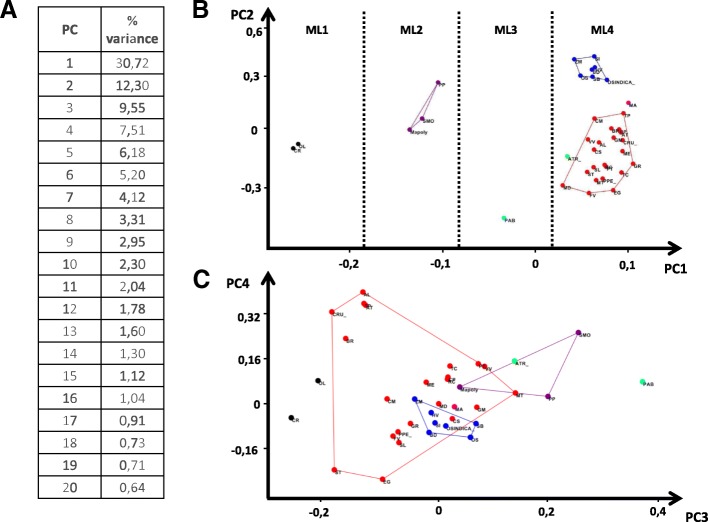
Principal component analysis of quantitative composition of the gene network corresponds to morphogenesis complexity. Given the percentage of variance corresponding to each component (**a**) and scatter plots of 1–2 (B) and 3–4 (C) components. Dots represent species, labels according to Additional file 2. Morphogenesis complexity groups (**b**) as indicated. MC1 - Single cell plants without any tissues, MC2 - multicellular plants with some tissues. Epidermal outgrowths presented as rhizoid and in some cases papillae, MC3 - (Gymnosperms) multicellular plants with distinct tissues types. Epidermal tissues contain several cell types. They have a pattern of root hairs, MC4 - (Flowering plants) multicellular plants with distinct tissue types. Epidermal tissues contain several cell types. They have a pattern of root hairs and a wide range of trichome types

Figure 4c shows the scattering of the species for the first two principal components, they describe 43% of the variance (30.7 and 12.3%, respectively). It can be noted that the plant taxa are ranked along the first component according to the increasing complexity of morphogenesis (Fig. 4 ML1-ML4).

Thus, the first component discriminates the general complication of the morphogenetic pattern. Along this component, some representatives of various orthologous genes were found These are MYB components of MBW trichome initiation complex (AT4G38620 (MYB4), AT2G16720, AT4G34990 (MYB7)), main gene trichome initiation AT1G79840 (GL2) and fractions of genes related to hormonal signal transduction (AT1G66350 (RGL1); AT3G03450 (RGL2); AT5G17490 (RGL3); AT2G01570 (RGA1); AT1G14920 (GAI); AT2G27300 (ANAC040); AT4G24210 (SLY1)) and to cytoskeleton structure (AT3G50530 (CRK); AT5G28646 (WVD2); AT5G43900 (MYA2); AT1G19835 (FPP4); AT2G35110 (GRL); AT2G46225 (ABIL1); AT5G42030 (ABIL4); AT5G24310 (ABIL3); AT4G01710 (CRK); AT5G65274; AT5G42080 (DL1)). We detected also 2 genes related to cell cycle dynamics (AT2G42260 (UVI4); AT4G15415) and 5 specific trichome differentiation genes (AT1G64690 (BLT); AT1G69490 (NAP); AT1G05230 (HDG2); AT4G21750 (ATML1); AT4G04890 (PDF2)).

The second component does not allow such an unambiguous biological interpretation, but it clearly separates the evolutionary lineages of dicots and monocots. Along this component, certain representatives of various orthologous genes were found. These are inhibitors of the MBW trichome initiation complex). It is known that the inhibitors of the initiator complex play the key role in the formation of the trichome pattern in *Arabidopsis* [6–13]. This reflects different pattern complexity of external outgrowth in monocots and dicots. More MBW inhibitors in dicots most likely raise the number of the degrees of freedom to form two-dimensional patterns on different organs in dicots. Monocots have leaf epidermis (like the root) in the form of cell rows or files. Perhaps, a similar nature of the pattern makes it possible to use a less flexible molecular system for its formation. Another explanation is the presence of a large number of TTG1 orthologs with variation in the domain composition in monocotyledonous plants, which can work in a similar way as a set of inhibitors.

Along this component the number of representatives of a various orthologous genes were found. Among them should be noted 3 genes - different components of MBW trichome initiation complex (AT4G00480 (ATMYC1), AT4G09820 (TT8), AT5G41315(GL3)), 9 genes related to hormonal signal transduction (AT2G46600; AT5G27320 (GID1C); AT3G05120 (GID1A); AT3G63010 (GID1B); AT4G02570 (CUL1); AT4G20780 (CML42); AT5G06650 (GIS2), AT2G41940 (ZFP8), AT3G58070 (GIS)), 7 genes related to cytoskeleton structure and dynamics (AT1G29170 (WAVE2), AT2G34150 (ATRANGAP2), AT2G38440 (SCAR2); AT1G80350 (ERH3); AT3G16630 (KINESIN-13A); AT5G20490 (XIK), AT1G17580 (MYA1)) and 4 specific trichome differentiation genes (AT1G01520 (ASG4); AT1G33240 (GTL1); AT2G02480 (STI); AT4G12610 (RAP74)).

Figure 4b shows the scattering of the species for the third and fourth principal components, both components describe 17% of the variance (9.5 and 7.5% respectively). Third components separates gymnosperms flowering plants and algae. The fourth component do not separate flowering plants and algae. The greatest PC4 value have *Brassicaceae*, *Solanum lycopersicum* and *Eucalyptus grandis*. However, the biological interpretation of these data is difficult.

The number of specific trichome differentiation genes (AT1G6949, AT1G01510) several genes related to hormonal signal transduction (AT2G46600, AT2G39940, AT2G39940) and to cytoskeleton structure (AT5G43900) have the greatest impact on the third component. A greatest impact to the third component have the number of genes containing MBW trichome initiation complex (AT1G01380, AT2G30420, AT2G30424, AT2G30432, AT2G46410, AT4G01060, AT5G53200), genes related to hormonal signal transduction (AT2G27300, AT2G27300), and related to cell cycle dynamics (AT5G45190, AT4G19600), related to cytoskeleton structure (AT2G46225, AT5G42030, AT5G24310) specific trichome differentiation genes (AT1G05230, AT4G21750, AT4G04890, AT1G79840).

Therefore, it can be concluded that orthologous genes of the main components of the MBW trichome initiation complex are present in both dicotyledonous and monocotyledonous plants, which suggests that the gene network studied already existed in the common ancestor of flowering plants. Thus, the hypothesis about the unique mechanisms of trichome formation in *A. thaliana* in the light of modern data requires revision [30]. Characteristic domain architecture of the components of the complex detected at the level of the common ancestor of gymnosperms and earlier (Fig. 2, Additional files 3, 4, 5 and 6). This allows us to infer that the MBW complex is a relatively ancient structure. Epidermal outgrowths of epidermal cells are widespread and also ancient formations. Simple outgrowths are found in algae - Chara (*Charophytales*) and *Spirogyra* (*Zygnematales*) [36]. Rhizoids in mosses have a characteristic pattern and perform the functions of fixation in the substrate, involved in the absorption of water and nutrients [37]. It was revealed that *Physcomitrella patens* genes PpRSL1 and PpRSL2 affect the number of rhizoids on the plant [38, 39]. Mutants of *Arabidopsis* devoid of the function of RHD6 (one of the key genes of hair development), develop root hairs if they are transformed by the genes PpRSL1 from *Physcomitrella*. This indicates that the function of the RSL family proteins has not been lost for 420 million years of the species divergence [38]. At the same time, a number of duplications of the HLH genes occurred before the divergence of dicots and monocots. This corresponds to the information that the ectopic expression of the rice R3 MYB transcription factor OsTCL1 in the *Arabidopsis* genome influences trichome formation [31]. At the same time, changes in OsTCL1 expression in rice do not lead to any trichome-related phenotypic changes indicating important functional differences between the operation of corresponding GRNs in these species [31]. In *Brassicaceae*, we observe acceleration of the accumulation of substitutions in proteins containing the HLH domain and some MYB factors (Additional files 4, 5 and 6). At the same time, we observe a series of duplication events and domain rearrangements in TTG1 orthologous in rice (Fig. 2d, (Additional files 3, 4, 5 and 6). Thus, at the molecular level of regulation by HLH in cereal plants, the GRN complex has more degrees of freedom and can potentially perform a wider range of particular functions.

The details of structural and functional evolution of proteins could be solved directly, through the 3D structure construction and investigation and indirectly, by simple prediction of structural residue types based on protein similarity. Finally, we chose the latter option to analyze the structural evolution of the proteins under study. This option, which is significantly less expensive in terms of computation, allowed us to demonstrate that there were at least two opposite trends of evolution of plant protein in the MBW complex: one is to change the protein surface (TTG1 and EGL3) leaving the inner structure of the protein globule conservative, while the other one is to change the inner structure of the protein globule (CPC and GLABRA), mainly by optimization of disordered regions.

It should be noted that the MBW complex, together with its regulators, directly participates in the inhibition of morphogenesis of root hairs [7, 9, 10, 32]. Thus, there is a reason to suggest that the variations of one gene network are responsible for the formation of the trichome pattern of leaf epidermis and of root hairs in *A. thaliana* [33]. Using RNA-seq data, Huang showed that the main set of genes responsible for root hairs is preserved at the evolutionary distances up to 200 million years or more [34]. However, the patterns of expression of these genes can vary significantly between different species [35]. Using the principal component method, we established 18 main orthological groups (30 individual nodes in GRN *A. thaliana*), corresponding to epidermal morphogenesis complexity in the evolutionary aspect.

## Conclusions

The main players (genes) of the initiator complex are old and probably had a similar function in the ancestor of all vascular plants to form a simple one-dimensional pattern. Duplications and gene losses are revealed in various evolutionary lines. Various trends in monocotyledonous and dicotyledonous plants were identified. Gene networks of organisms with a more complex pattern have passed through a huge number of duplication events of individual genes that probably played a role in the formation of complex patterns. However, monocotyledonous and dicotyledon patterns are formed by the same gene networks in complexity.

- In the gene network of development, the following functional blocks are distinguished by trichomes (hormone-responsive regulators, initiator complex and its inhibitors, cytoskeleton genes, cell cycle genes, and other);
- The ancestor of all vascular plants already had all these elements but less of them;
- The number of candidate genes responsible for development of trichomes was predicted for a wide range of species.

## Methods

### GRN reconstruction

According to the analysis of associated GO terms based on the GeneOntology [68], TAIR [69], PLAZA databases [70], 90 genes associated with trichome formation in *A. thaliana* were found (negative regulation of trichome patterning, trichome branching, regulation of trichome morphogenesis, trichome morphogenesis, trichome differentiation, trichome patterning). The enriched set of genes/proteins was obtained based on protein-protein-interaction data hosted in Cytoscape databases. The preliminary list of genes and their interactions was enriched by additional interactions (regulatory, protein-protein, etc.) using databases that store regulatory and other (STRING [71], Cytoscape [72] and Andsystem [73]). This list included 100 genes (Tabel 1). Additionally, we used the GeneMania dataset to check the gene-to-gene network interrelation and to enrich the gene set. Also, genes were added from the STRING database and using Cytoscape program (GeneMania plugin). Genes which had the highest score of connection with our sample were chosen for. Expert gentrification of genes was carried out, taking into account the functional annotation, mention in peer-reviewed articles and connectivity in the gene network.

### Sequences databases and phylogenetic analysis

To clarify the evolutionary pathway of the trichome-related gene network, we investigated the phylogenetic relationships between all of the homologs available in fully sequenced genomes in PLAZA 3.0 (http://bioinformatics.psb.ugent.be/plaza/ [70]. A BLAST+ [74, 75], we conducted sequence retrieval to form a list of sequences with significant similarity (E value <1e-5) to the A.thaliana GRN components. Using the reciprocal blasts of the search, the most complete groups of homologues were obtained. Identification of domains in proteins was carried out using the hmmsearch program of the package HMMER v.3 (http://hmmer.org/) [76] and the Hidden Markov Models (Hidden Markov Model – HMM) taken from the pfam database (https://pfam.xfam.org/) using the threshold e-value = 1e-7. Proteins that did not contain any domains corresponding to the query were excluded from the analysis. Multiple alignment of the proteins was performed using mafft 7 [77] with parameters “--add” “--auto” and “--keeplength”. Automatic cleaning of multiple alignments from uninformative sites (a site in which more than 80% of proteins have a gap) was made using an in-house script written in Python. In addition, the proteins having more than 75% of the gaps in the alignment after cleaning were removed from the analysis. An expert evaluation of the alignment was carried out to identify proteins with significant deletions in the domains. In the case of finding such a tree, topology was verified by constructing a tree without the detected defective sequences. Analysis of molecular evolution was carried out with the help of a pipeline SAMEM v. 0.82 [78]. The construction of the model of amino acid substitutions based on multiple alignment was carried out by the algorithm Modelestimator [79]. FastTree 2.1.1 [80] was used for estimating the primary topology. The construction of the final phylogenetic tree on the basis of previously generated substitution model was carried out by Phyml [81] by optimization of primary tree topology and branch lengths. To test the stability of the tree branching points, we used the aLRT procedure. Tree visualization and topology analysis were performed in programs FigTree v.1.4.2 [82], Archaeopteryx (https://sites.google.com/site/cmzmasek/home/software/archaeopteryx) and ETE toollkit [83]. In the trees, according to the topology and OTU (Operational Taxonomic Units), species assessment was made, the information on the protein functions and their domain composition was specified. Clades of orthological sequences and the time of diversification of the corresponding functions of the proteins were estimated. Evolutionally, later duplications that arose after the divergence of flowering plants by families and orders of flowering plants were estimated by counting the number of sequences in the respective monophyletic groups.

Principal component analysis results were incorporated into the procedures for reducing the number of metrical and morphological variables. We performed principal component analysis (PCA) to ordinate the different number of genes in different evolutionary lines. To describe morphological variability, we calculated the principal component using the PAST statistical program, version 2 [84]. In this case, the signs were the number of genes in the orthologic group, and the objects were the species themselves. Before the analysis, the number of genes in each orthologic group was normalized to eliminate noise. The number of orthologues in each species is given in Additional file 2.

### In-depth analysis of the evolution of GRN proteins

To conduct in-depth analysis of the evolution of GRN components, we selected 4 objects of investigations (protein families): EGL3, GLABRA, CPC, TTG1. The multiple protein alignments of CPC proteins, which had been previously constructed by MAFFT, were additionally refined by PROMALS. Selection of alignment regions for phylogenetic analysis was done sequentially by GBlocks [85] and by manual alignment checking (selecting out gap-enriched sequences in core alignment blocks). The best matrices (models) of relative rates of amino acid substitutions were selected by IQTree 1.5.4. For all four protein alignments, JTT + G4 was found to be the best model. This model was used for reconstruction of initial tree topologies. Initial protein tree topologies were corrected using the Viridiplantae species tree from TimeTree DB using the TreeFix v1.1.10 software, after that reoptimization of the branch lengths was done by the IQTree 1.5.4 and JTT + G4 model.

MCMC phylogenetic-tree-aware Bayesian sampling of ancestral sequences in each inner node of four trees was conducted using the PhyloBayes 4.1, CAT evolutionary model and 6 discrete categories of site evolutionary rates. The MCMC sampling was used for full and sequestered (using modified approach called ‘AltAll*N’) ancestral libraries generation. Our ‘AltAll*N’ procedure is iterative rewriting of all plausible (posterior probability> 0.1) alternative states in the ancestral sequences at each inner tree node. For instance, if there are 3 alternative states in site A and 4 alternative states in site B of ancestral node X we should rewrite ancestral sequence 4 times to obtain 4 alternative ancestors in node X: a) a sequence composed of best states in A and B sites, b) a sequence with second probable states of A and B, c) a sequence with third probable states of A and B, and d) a sequence with the third probable states of A and the forth probable states of B.

In order to find the epistatic conversion signatures or the evolutionary ‘Stokes shifts’, we analyzed deviation of protein evolution from the protein-specific matrix of the relative rates of amino acid substitutions on each of the protein tree branches (1) and simply compared the inner branch lengths calculation based on protein structure data (2).

(1) To compare the branch-specific rates of amino acid substitutions with whole tree-specific representing as a matrix of the relative rates of amino acid substitutions, we used full ancestral libraries. To do that, we consecutively took the following steps: a) reconstruction of the protein-specific time-reversible model of amino acid replacement relative rates (model estimator software) for alignments of extant protein sequences of 4 trees under analysis; b) *d* measure calculation for each possible substitution of each inner tree node, *d* = *PP*a*PPb*2*NC, where *PP*a and *PP*b are the posterior probabilities of a and b amino acids, a is not equal to b, *NC* = 1/(1 + e^(200*RFab)), *RF*ab is the relative rate of ab substitution in the protein-specific time-reversible matrix of amino acid substitutions; c) summing the *d* measures across all sites in each inner tree node and the calculating the natural logarithms of these sums; d) nonparametric comparison (by percentiles) of log-sums across all tree in order to identify branches with maximal log-sum (branches with epistatic conversions signatures).

(2) To compare the branch-specific rates of structural changes, we used sequestered ‘AltAll*N’ ancestral libraries. To do that, we consecutively took the following steps: a) deducing the secondary structure, the surface state and the disorder signature for each residue of each alternative ancestral sequence in each inner tree node using RaptorX_Property Fast pipeline [86]; b) computation of the change frequencies for secondary structures, surface states and disorder signatures between all the alternative ancestral sequences of neighboring inner tree nodes; c) nonparametric comparison (by percentiles) of the above change frequencies across all the tree in order to identify branches with maximal structural changes.

## Additional files

Additional file 1: **Table S1:** Table contains the following sheets: GRN_statistics - this sheet contains information on the number of nodes and edges the network. GO_enricment - this sheet contains information about the GO enrichment of all nodes from the network (according to AgriGO, the date of appeal is 11/07/2018). GO_terms_associated_GRN_nodes - this sheet contains information about the terms assigned to each node from the network (according to TAIR, the date of appeal is 11/07/2018). GO terms of GRN clusters - This sheet contains information about which term belongs to which cluster of the gene network. (XLSX 64 kb)

Additional file 2: **Table S2:** Table contains the following sheets: Number of ortologous genes - This sheet contains information on the number of orthologous genes for each gene from the gene network in the species research. Evolutionary characteristics of GRN nodes - This sheet contains information on duplication events in different evolutionary lines for each gene from the gene network. (XLSX 26 kb)

Additional file 3: **Figure S1: A.** PhyML phylogenetic relations and composition of domains for EGL3 (AT1G63650), GL3 (AT5G41315), MYC1 (AT4G00480) and TT8 (AT4G09820) homologues of representative plant species. **B.** Evolutionary changes in protein structure of the EGL3 (AT1G63650), GL3 (AT5G41315), MYC1 (AT4G00480) and TT8 (AT4G09820) homologous proteins being studied branch reflects from right to left: disorder (2 residue types), secondary structure (3 types), secondary structure (8 types), globule surface (3 residue types), rare (comparing with protein specific model) amino acid substitutions. **Color scheme:** black- outer branch (not analyzed); colours define branch lengths quartile: blue – Q1; green – Q2; orange –Q3; red –Q4. (PDF 5022 kb)

Additional file 4: **Figure S2: A.** PhyML phylogenetic relations and composition of domains for TTG1 (AT5G24520) homologues of representative plant species. **B.** Evolutionary changes in protein structure of the TTG1 (AT5G24520) homologous proteins being studied branch reflects from right to left: disorder (2 residue types), secondary structure (3 types), secondary structure (8 types), globule surface (3 residue types), rare (comparing with protein specific model) amino acid substitutions. Color scheme: black- outer branch (not analyzed); colours define branch lengths quartile: blue – Q1; green – Q2; orange –Q3; red –Q4. (PDF 4064 kb)

Additional file 5: **Figure S3: A.** PhyML phylogenetic relations and composition of domains for R2R3-MYB (AT1G66370; AT1G566650; AT1G66390; AT1G66380; AT5G35550; AT5G14750; AT5G40330; AT3G27920; AT5G52600) homologues of representative plant species. **B.** Evolutionary changes in protein structure of the R2R3-MYB (AT1G66370; AT1G566650; AT1G66390; AT1G66380; AT5G35550; AT5G14750; AT5G40330; AT3G27920; AT5G52600) homologous proteins being studied branch reflects from right to left: disorder (2 residue types), secondary structure (3 types), secondary structure (8 types), globule surface (3 residue types), rare (comparing with protein specific model) amino acid substitutions. Color scheme: black- outer branch (not analyzed); colours define branch lengths quartile: blue – Q1; green – Q2; orange –Q3; red –Q4. (PDF 5149 kb)

Additional file 6: **Figure S4: A.** PhyML phylogenetic relations and composition of domains for R3-MYB homologues of representative plant species. **B.** Evolutionary changes in protein structure of the R3-MYB homologous proteins being studied branch reflects from right to left: disorder (2 residue types), secondary structure (3 types), secondary structure (8 types), globule surface (3 residue types), rare (comparing with protein specific model) amino acid substitutions. Color scheme: black- outer branch (not analyzed); colours define branch lengths quartile: blue – Q1; green – Q2; orange –Q3; red –Q4. (PDF 3855 kb)

Additional file 7: **Figure S5:** PhyML phylogenetic relations and composition of domains for AT1G01510 homologues of representative plant species. **Figure S6:** PhyML phylogenetic relations and composition of domains for AT1G01520 homologues of representative plant species. **Figure S7:** PhyML phylogenetic relations and composition of domains for AT1G03060 homologues of representative plant species. **Figure S8:** PhyML phylogenetic relations and composition of domains for AT1G05230; AT4G21750; AT4G04890; AT1G79840 homologues of representative plant species. **Figure S9**: PhyML phylogenetic relations and composition of domains for AT1G67030; AT3G58070; AT2G41940; AT5G06650; AT1G10480; AT1G68360 homologues of representative plant species. **Figure S10**: PhyML phylogenetic relations and composition of domains for AT1G13180 homologues of representative plant species. **Figure S11**: PhyML phylogenetic relations and composition of domains for AT1G66350; AT5G17490; AT3G03450; AT2G01570; AT1G14920 homologues of representative plant species. **Figure S12**: PhyML phylogenetic relations and composition of domains for AT1G17920; AT1G73360 homologues of representative plant species. **Figure S13**: PhyML phylogenetic relations and composition of domains for AT1G19835 homologues of representative plant species. **Figure S14**: PhyML phylogenetic relations and composition of domains for AT1G25540 homologues of representative plant species. (PDF 10138 kb)

Additional file 8: **Figure S15**: PhyML phylogenetic relations and composition of domains for AT1G29170; AT2G34150; AT2G38440 homologues of representative plant species. **Figure S16:** PhyML phylogenetic relations and composition of domains for AT2G33385; AT1G30825 homologues of representative plant species. **Figure S17:** PhyML phylogenetic relations and composition of domains for AT1G33240 homologues of representative plant species. **Figure S18**: PhyML phylogenetic relations and composition of domains for AT1G60430 homologues of representative plant species. **Figure S19**: PhyML phylogenetic relations and composition of domains for AT1G64690 homologues of representative plant species. **Figure S20**: PhyML phylogenetic relations and composition of domains for AT1G65470 homologues of representative plant species. **Figure S21**: PhyML phylogenetic relations and composition of domains for AT5G38110; AT1G66740 homologues of representative plant species. **Figure S22**: PhyML phylogenetic relations and composition of domains for AT1G69490 homologues of representative plant species. **Figure S23**: PhyML phylogenetic relations and composition of domains for AT1G75950 homologues of representative plant species. **Figure S24**: PhyML phylogenetic relations and composition of domains for AT1G80350 homologues of representative plant species. (PDF 7989 kb)

Additional file 9; **Figure S25**: PhyML phylogenetic relations and composition of domains for AT2G02480 homologues of representative plant species. **Figure S26**: PhyML phylogenetic relations and composition of domains for AT2G22640 homologues of representative plant species. **Figure S27**: PhyML phylogenetic relations and composition of domains for AT2G27300 homologues of representative plant species. **Figure S28**: PhyML phylogenetic relations and composition of domains for AT2G27600 homologues of representative plant species. **Figure S29**: PhyML phylogenetic relations and composition of domains for AT2G31300 homologues of representative plant species. **Figure S30**: PhyML phylogenetic relations and composition of domains for AT2G33540 homologues of representative plant species. **Figure S31**: PhyML phylogenetic relations and composition of domains for AT2G35110 homologues of representative plant species. **Figure S32**: PhyML phylogenetic relations and composition of domains for AT2G39940 homologues of representative plant species. **Figure S33**: PhyML phylogenetic relations and composition of domains for AT2G42260 homologues of representative plant species. **Figure S34**: PhyML phylogenetic relations and composition of domains for AT2G46225; AT5G42030; AT5G24310 homologues of representative plant species. (PDF 6990 kb)

Additional file 10: **Figure S35**: PhyML phylogenetic relations and composition of domains for AT2G46600 homologues of representative plant species. **Figure S36**: PhyML phylogenetic relations and composition of domains for AT3G12280 homologues of representative plant species. **Figure S37**: PhyML phylogenetic relations and composition of domains for AT3G12400 homologues of representative plant species. **Figure S38**: PhyML phylogenetic relations and composition of domains for AT3G16630 homologues of representative plant species. **Figure S39**: PhyML phylogenetic relations and composition of domains for AT3G27000 homologues of representative plant species. **Figure S40**: PhyML phylogenetic relations and composition of domains for AT3G50530 homologues of representative plant species. **Figure S41**: PhyML phylogenetic relations and composition of domains for AT5G27320; AT3G05120; AT3G63010 homologues of representative plant species. **Figure S42:** PhyML phylogenetic relations and composition of domains for AT4G01710; AT5G65274 homologues of representative plant species. **Figure S43**: PhyML phylogenetic relations and composition of domains for AT4G02570 homologues of representative plant species. **Figure S44**: PhyML phylogenetic relations and composition of domains for AT4G12610 homologues of representative plant species. (PDF 7692 kb)

Additional file 11: **Figure S45**: PhyML phylogenetic relations and composition of domains for AT4G14147 homologues of representative plant species. **Figure S46**: PhyML phylogenetic relations and composition of domains for AT4G15415 homologues of representative plant species. **Figure S47:** PhyML phylogenetic relations and composition of domains for AT4G20780 homologues of representative plant species. **Figure S48**: PhyML phylogenetic relations and composition of domains for AT4G22910 homologues of representative plant species. **Figure S49**: PhyML phylogenetic relations and composition of domains for AT4G24210 homologues of representative plant species. **Figure S50**: PhyML phylogenetic relations and composition of domains for AT4G38600 homologues of representative plant species. **Figure S51**: PhyML phylogenetic relations and composition of domains for AT4G04470 homologues of representative plant species. **Figure S52**: PhyML phylogenetic relations and composition of domains for AT5G06650; AT2G41940; AT3G58070 homologues of representative plant species. **Figure S53**: PhyML phylogenetic relations and composition of domains for AT5G18410 homologues of representative plant species. **Figure S54**: PhyML phylogenetic relations and composition of domains for AT1G17580; AT5G20490 homologues of representative plant species. (PDF 7225 kb)

Additional file 12: **Figure S55**: PhyML phylogenetic relations and composition of domains for AT5G20570 homologues of representative plant species. **Figure S56**: PhyML phylogenetic relations and composition of domains for AT2G36010; AT1G47870; AT5G22220 homologues of representative plant species. **Figure S57**: PhyML phylogenetic relations and composition of domains for AT5G24310; AT5G42030; AT2G46225 homologues of representative plant species. **Figure S58:** PhyML phylogenetic relations and composition of domains for AT5G28646 homologues of representative plant species. **Figure S59:** PhyML phylogenetic relations and composition of domains for AT5G42080 homologues of representative plant species. **Figure S60:** PhyML phylogenetic relations and composition of domains for AT5G43900 homologues of representative plant species. **Figure S61**: PhyML phylogenetic relations and composition of domains for AT5G45190; AT4G19600 homologues of representative plant species. **Figure S62**: PhyML phylogenetic relations and composition of domains for AT2G33540 homologues of representative plant species. **Figure S63**: PhyML phylogenetic relations and composition of domains for AT5G58230 homologues of representative plant species. **Figure S64**: PhyML phylogenetic relations and composition of domains for AT5G64630 homologues of representative plant species. **Figure S65:** PhyML phylogenetic relations and composition of domains for AT5G65930 homologues of representative plant species. (PDF 9760 kb)

## Abbreviations

bHLH: basic helix-loop-helix
C2H2: zinc finger C2H2-type
GRN: Gene regulatory networks
MBW: a special protein complexes contain MYB–bHLH–WDR proteins
MYA: Million year ago
OTU: Operational taxonomic units
PC: Principal component
