# Supplementary material for: The evolution of gene regulatory networks controlling Arabidopsis thaliana L. trichome development
Source: BMC Plant Biol. 2019 Feb 15;19(Suppl 1):53. doi: 10.1186/s12870-019-1640-2 (PMC6393967; doi:10.1186/s12870-019-1640-2)

Figure S1A PhyML phylogenetic relations and composition of domains for EGL3 (AT1G63650), GL3 (AT5G41315), MYC1 (AT4G00480) and TT8 (AT4G09820) homologues of representative plant species.

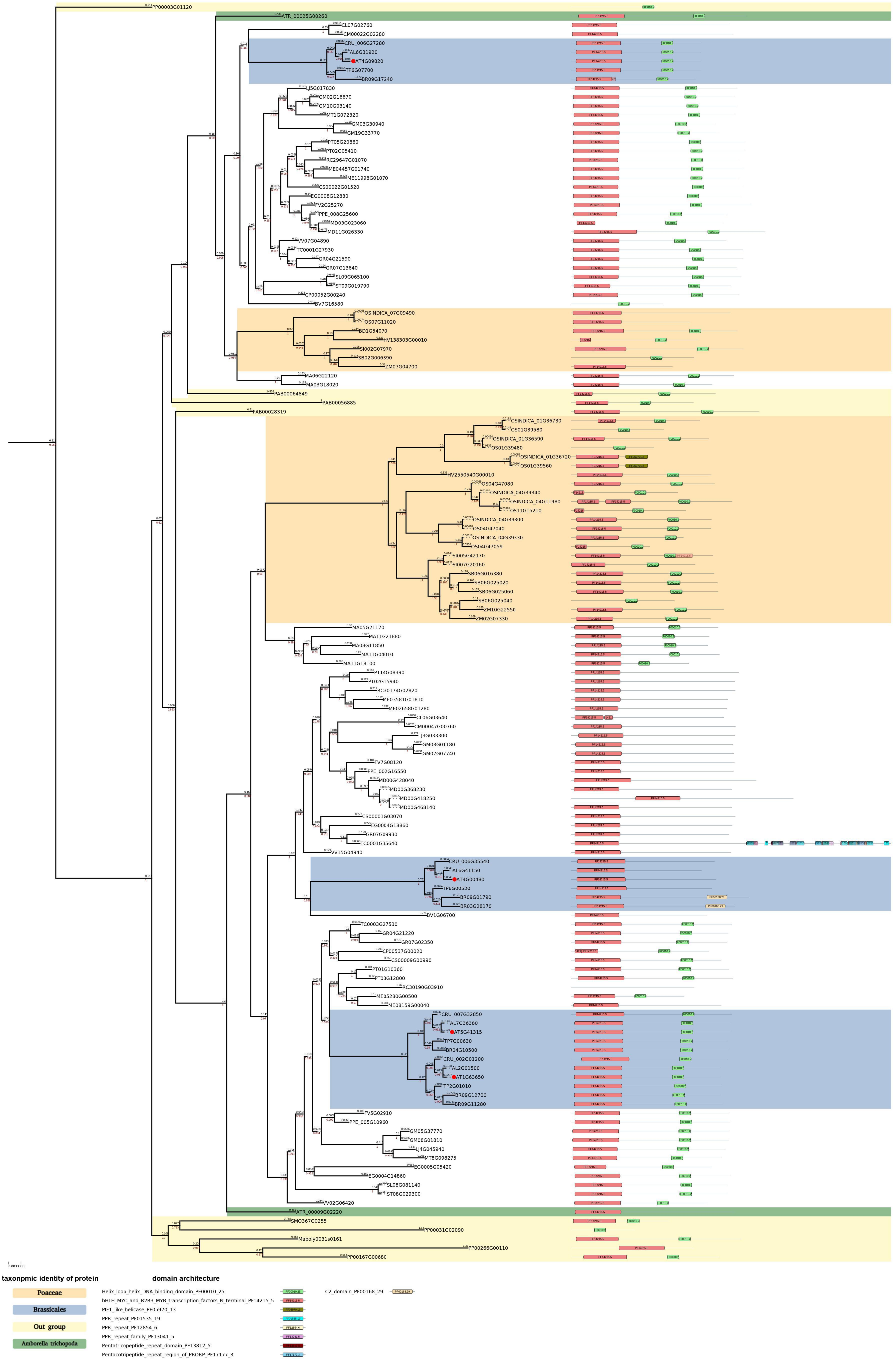

Figure S1B Evolutionary changes in protein structure of the EGL3 (AT1G63650), GL3 (AT5G41315), MYC1 (AT4G00480) and TT8 (AT4G09820) homologous proteins being studied branch reflects from right to left: disorder (2 residue types), secondary structure (3 types), secondary structure (8 types), globule surface (3 residue types), rare (comparing with protein specific model) amino acid substitutions
Color scheme:
black- outer branch (not analyzed);
colours define branch lengths quartile: blue – Q1; green – Q2; orange –Q3; red –Q4.

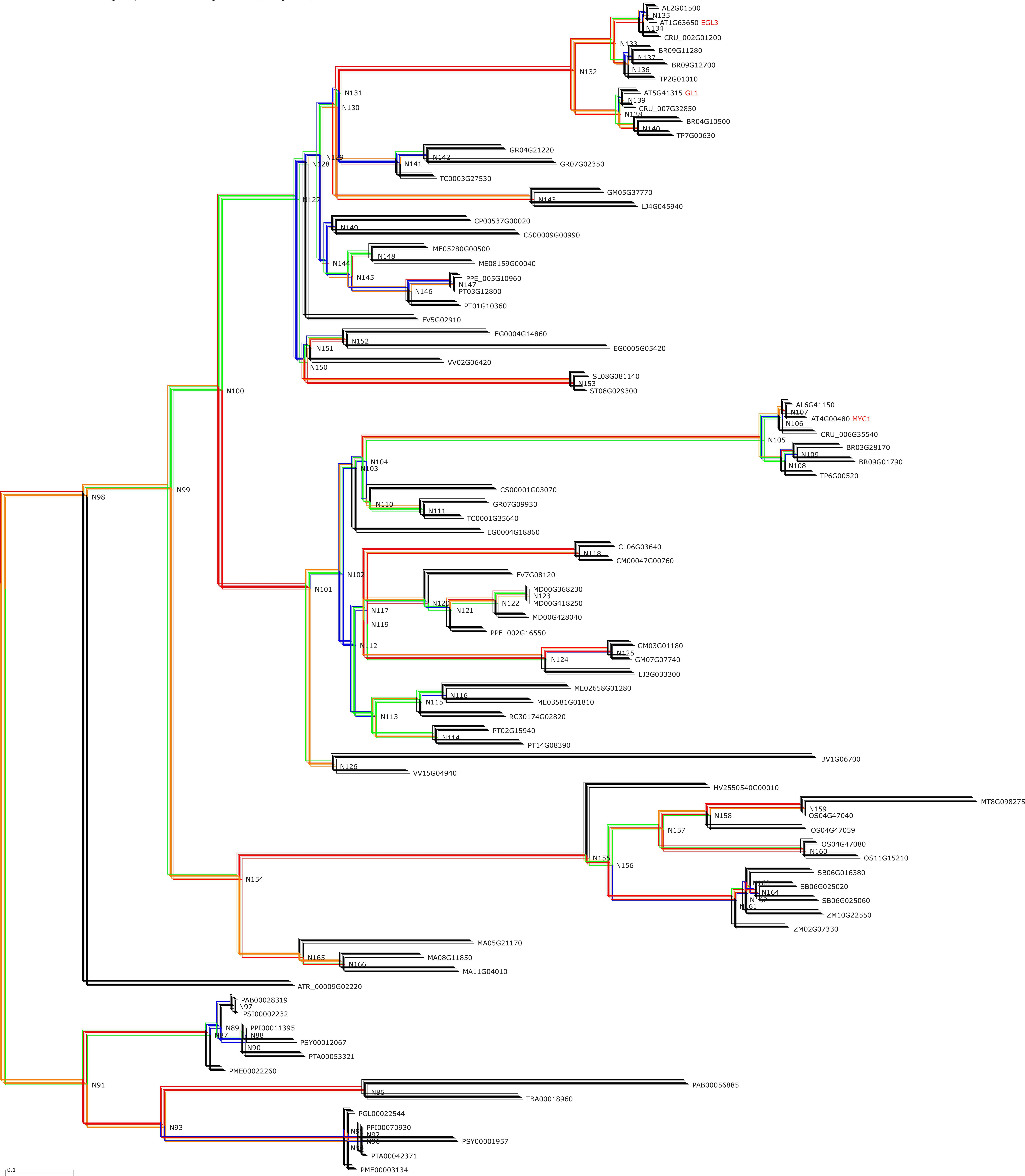

Supplement: Supplementary file 3 — Figure S1: A. PhyML phylogenetic relations and composition of domains for EGL3 (AT1G63650), GL3 (AT5G41315), MYC1 (AT4G00480) and TT8 (AT4G09820) homologues of representative plant species. B. Evolutionary changes in protein structure of the EGL3 (AT1G63650), GL3 (AT5G41315), MYC1 (AT4G00480) and TT8 (AT4G09820) homologous proteins being studied branch reflects from right to left: disorder (2 residue types), secondary structure (3 types), secondary structure (8 types), globule surface (3 residue types), rare (comparing with protein specific model) amino acid substitutions. Color scheme: black- outer branch (not analyzed); colours define branch lengths quartile: blue – Q1; green – Q2; orange –Q3; red –Q4. (PDF 5022 kb) [file 12870_2019_1640_MOESM3_ESM.pdf]
