# Supplementary material for: The evolution of gene regulatory networks controlling Arabidopsis thaliana L. trichome development
Source: BMC Plant Biol. 2019 Feb 15;19(Suppl 1):53. doi: 10.1186/s12870-019-1640-2 (PMC6393967; doi:10.1186/s12870-019-1640-2)

Figure S2A PhyML phylogenetic relations and composition of domains for TTG1 (AT5G24520) homologues of representative plant species.

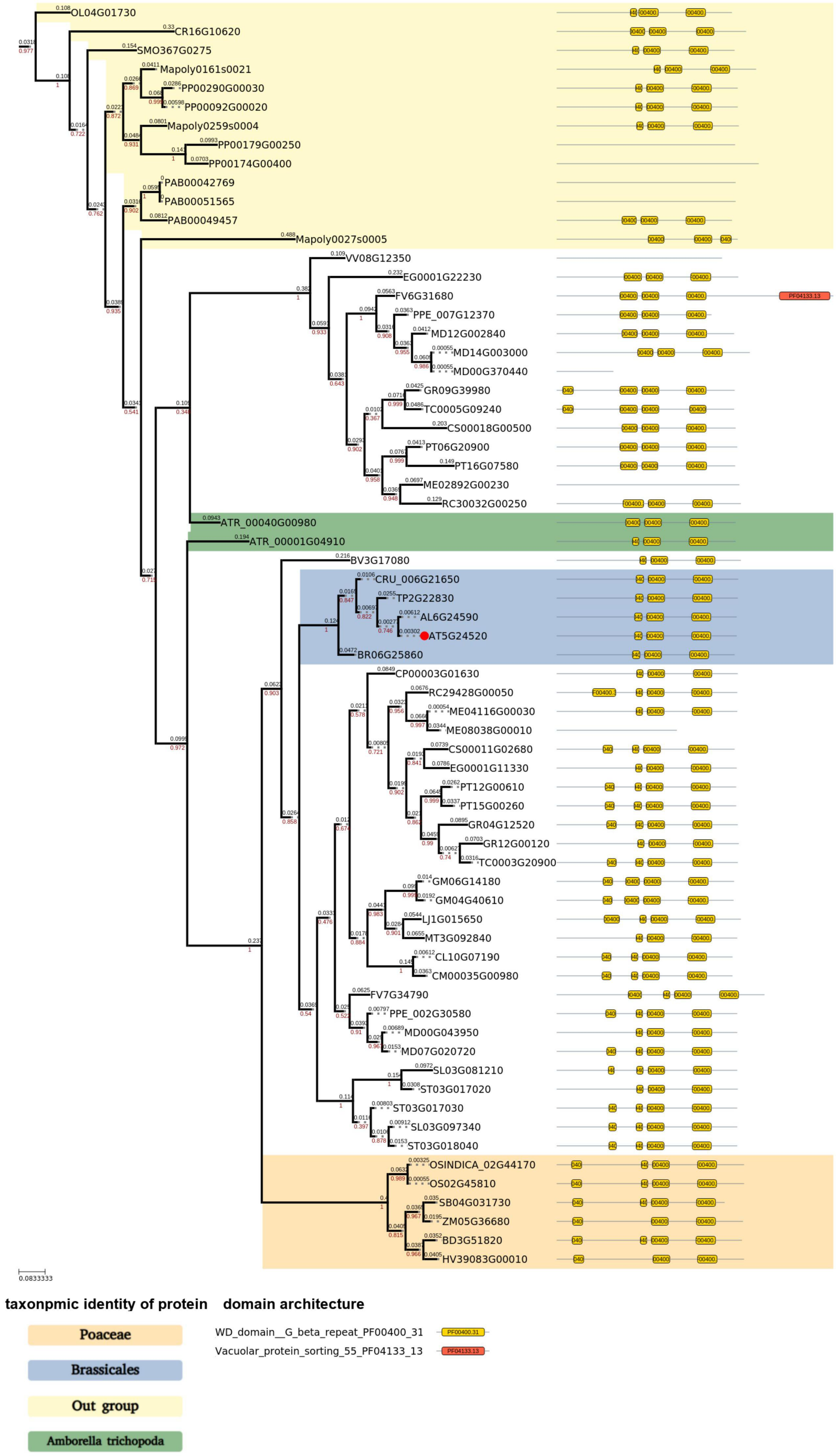

Figure S2B Evolutionary changes in protein structure of the TTG1 (AT5G24520) homologous proteins being studied branch reflects from right to left: disorder (2 residue types), secondary structure (3 types), secondary structure (8 types), globule surface (3 residue types), rare (comparing with protein specific model) amino acid substitutions
Color scheme:
black- outer branch (not analyzed);
colours define branch lengths quartile: blue – Q1; green – Q2; orange –Q3; red –Q4.

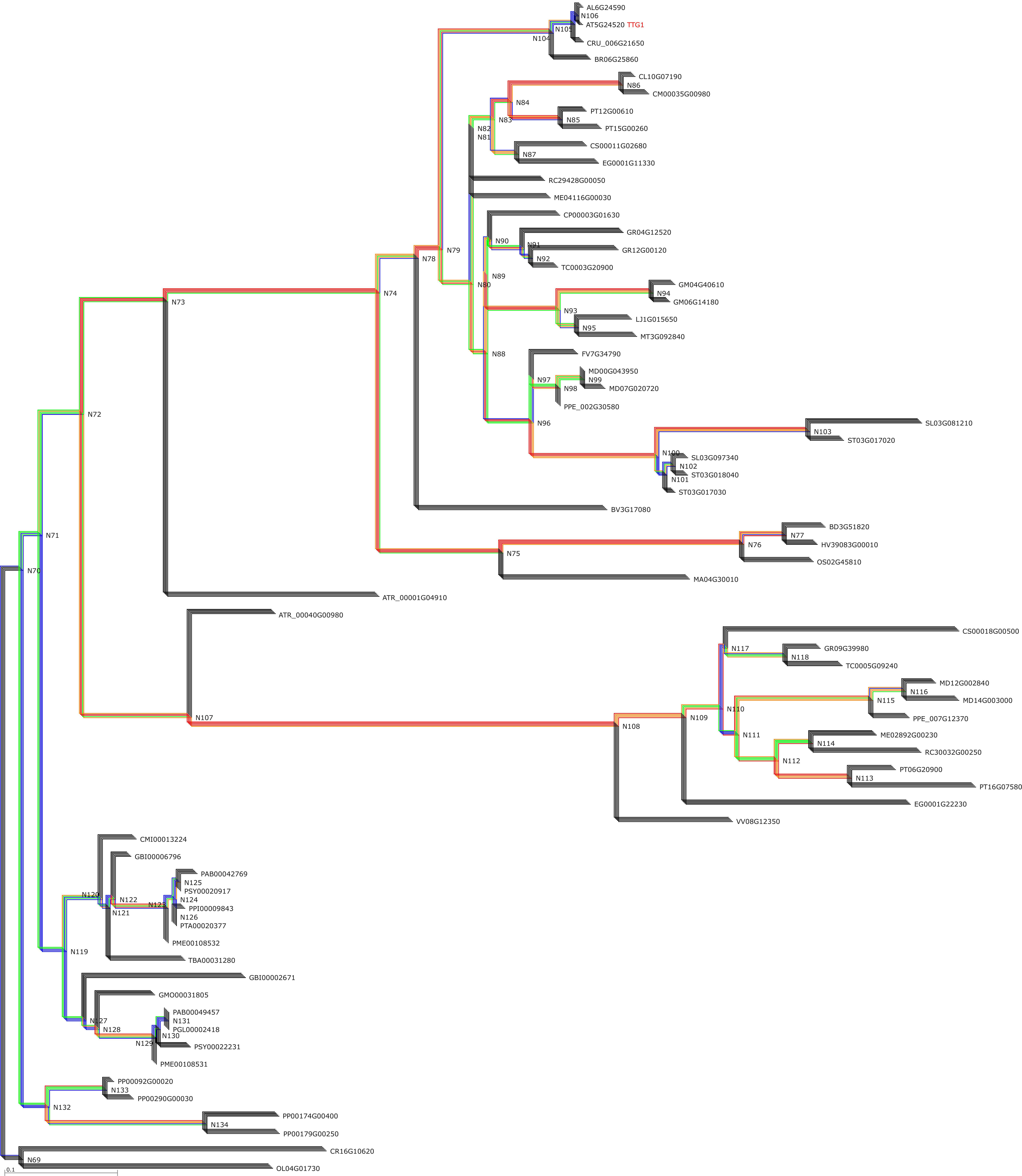

Supplement: Supplementary file 4 — Figure S2: A. PhyML phylogenetic relations and composition of domains for TTG1 (AT5G24520) homologues of representative plant species. B. Evolutionary changes in protein structure of the TTG1 (AT5G24520) homologous proteins being studied branch reflects from right to left: disorder (2 residue types), secondary structure (3 types), secondary structure (8 types), globule surface (3 residue types), rare (comparing with protein specific model) amino acid substitutions. Color scheme: black- outer branch (not analyzed); colours define branch lengths quartile: blue – Q1; green – Q2; orange –Q3; red –Q4. (PDF 4064 kb) [file 12870_2019_1640_MOESM4_ESM.pdf]
