# Supplementary material for: The evolution of gene regulatory networks controlling Arabidopsis thaliana L. trichome development
Source: BMC Plant Biol. 2019 Feb 15;19(Suppl 1):53. doi: 10.1186/s12870-019-1640-2 (PMC6393967; doi:10.1186/s12870-019-1640-2)

Figure S4A PhyML phylogenetic relations and composition of domains for R3-MYB homologues of representative plant species.

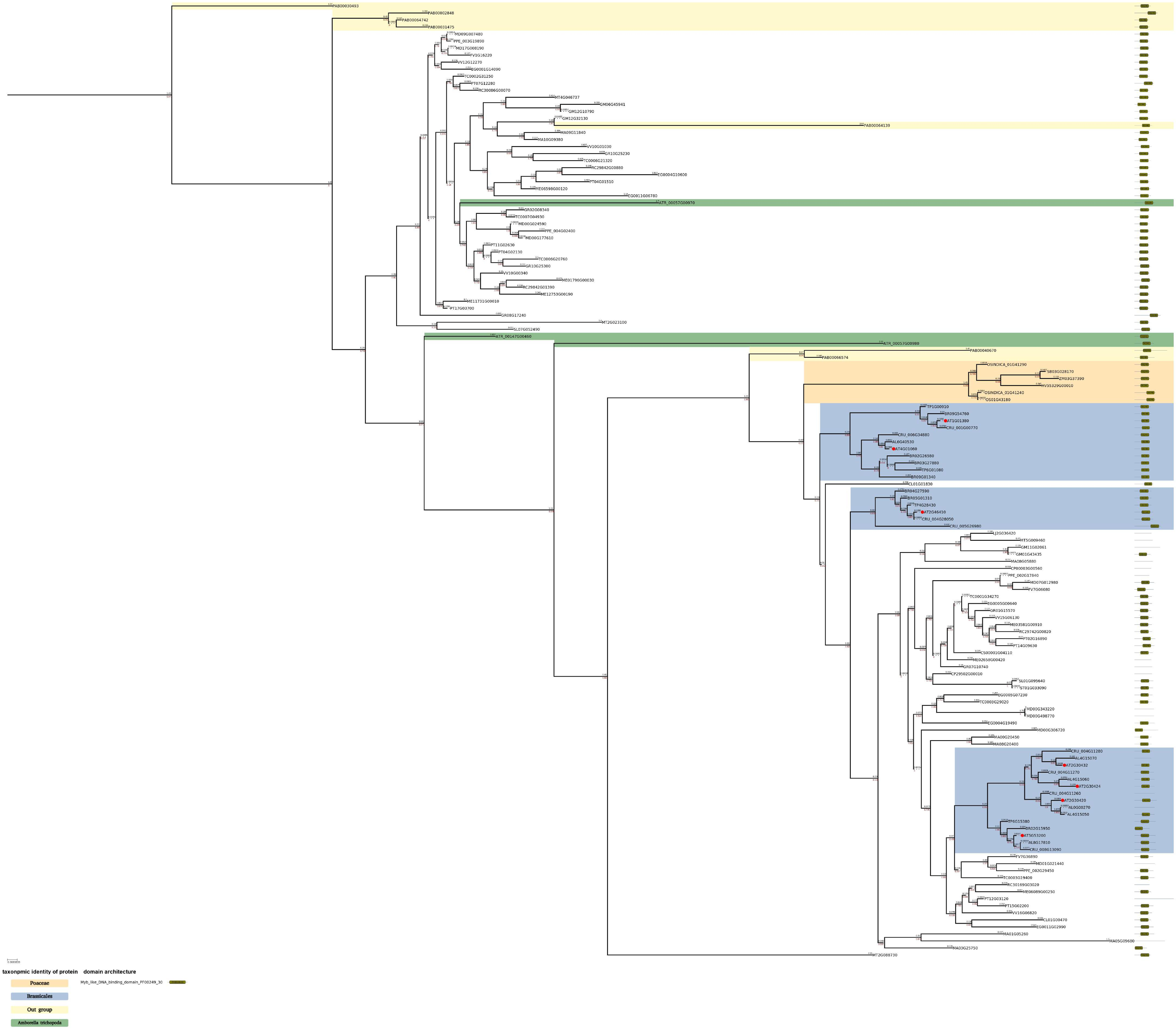

Figure S4B Evolutionary changes in protein structure of the R3-MYB homologous proteins being studied branch reflects from right to left: disorder (2 residue types), secondary structure (3 types), secondary structure (8 types), globule surface (3 residue types), rare (comparing with protein specific model) amino acid substitutions
Color scheme:
black- outer branch (not analyzed);
colours define branch lengths quartile: blue – Q1; green – Q2; orange –Q3; red –Q4.

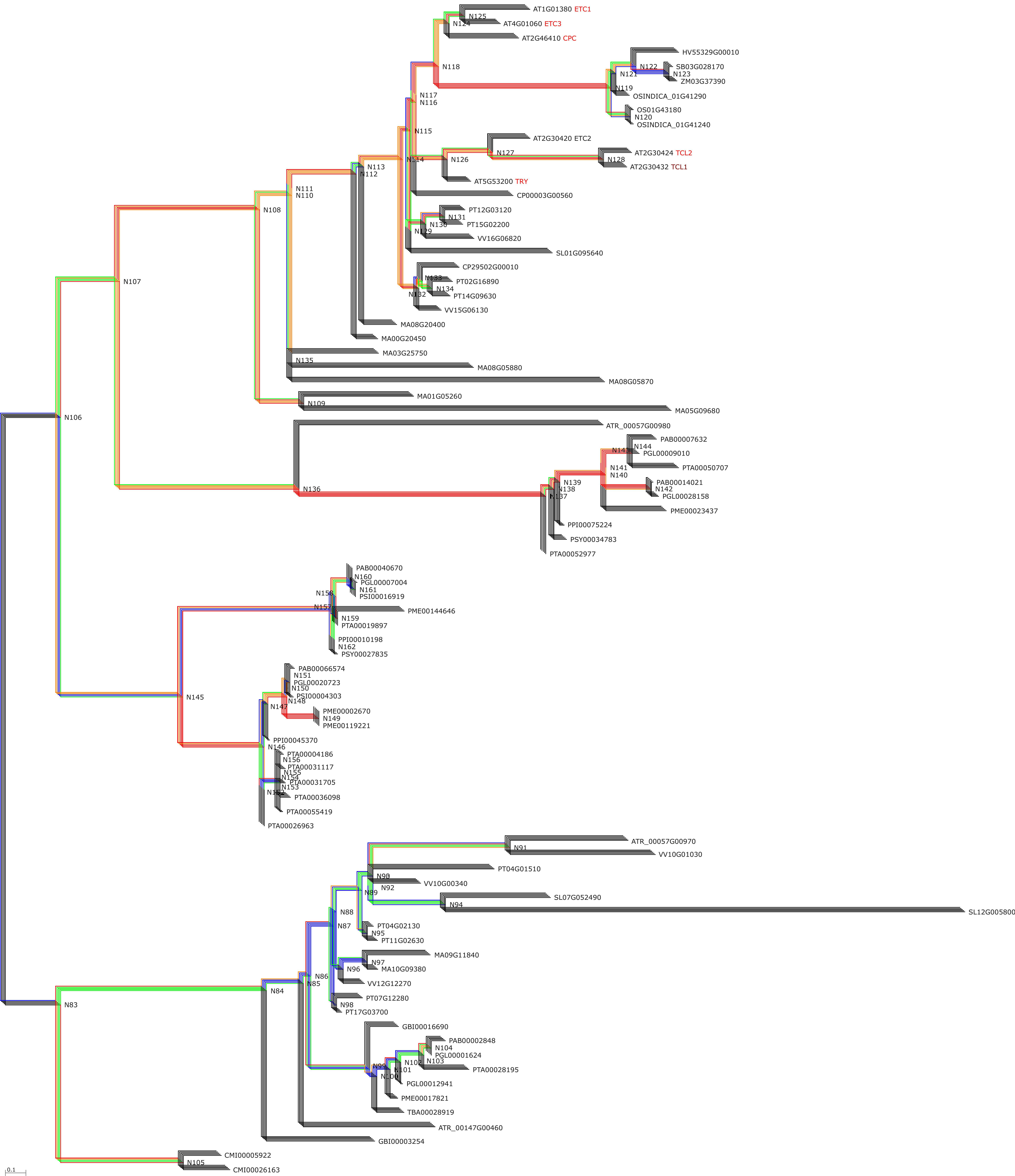

Supplement: Supplementary file 6 — Figure S4: A. PhyML phylogenetic relations and composition of domains for R3-MYB homologues of representative plant species. B. Evolutionary changes in protein structure of the R3-MYB homologous proteins being studied branch reflects from right to left: disorder (2 residue types), secondary structure (3 types), secondary structure (8 types), globule surface (3 residue types), rare (comparing with protein specific model) amino acid substitutions. Color scheme: black- outer branch (not analyzed); colours define branch lengths quartile: blue – Q1; green – Q2; orange –Q3; red –Q4. (PDF 3855 kb) [file 12870_2019_1640_MOESM6_ESM.pdf]
