# Supplementary material for: The evolution of gene regulatory networks controlling Arabidopsis thaliana L. trichome development
Source: BMC Plant Biol. 2019 Feb 15;19(Suppl 1):53. doi: 10.1186/s12870-019-1640-2 (PMC6393967; doi:10.1186/s12870-019-1640-2)

Figure S25. PhyML phylogenetic relations and composition of domains for AT2G02480 homologues of representative plant species. The color legends of species and domains is given below

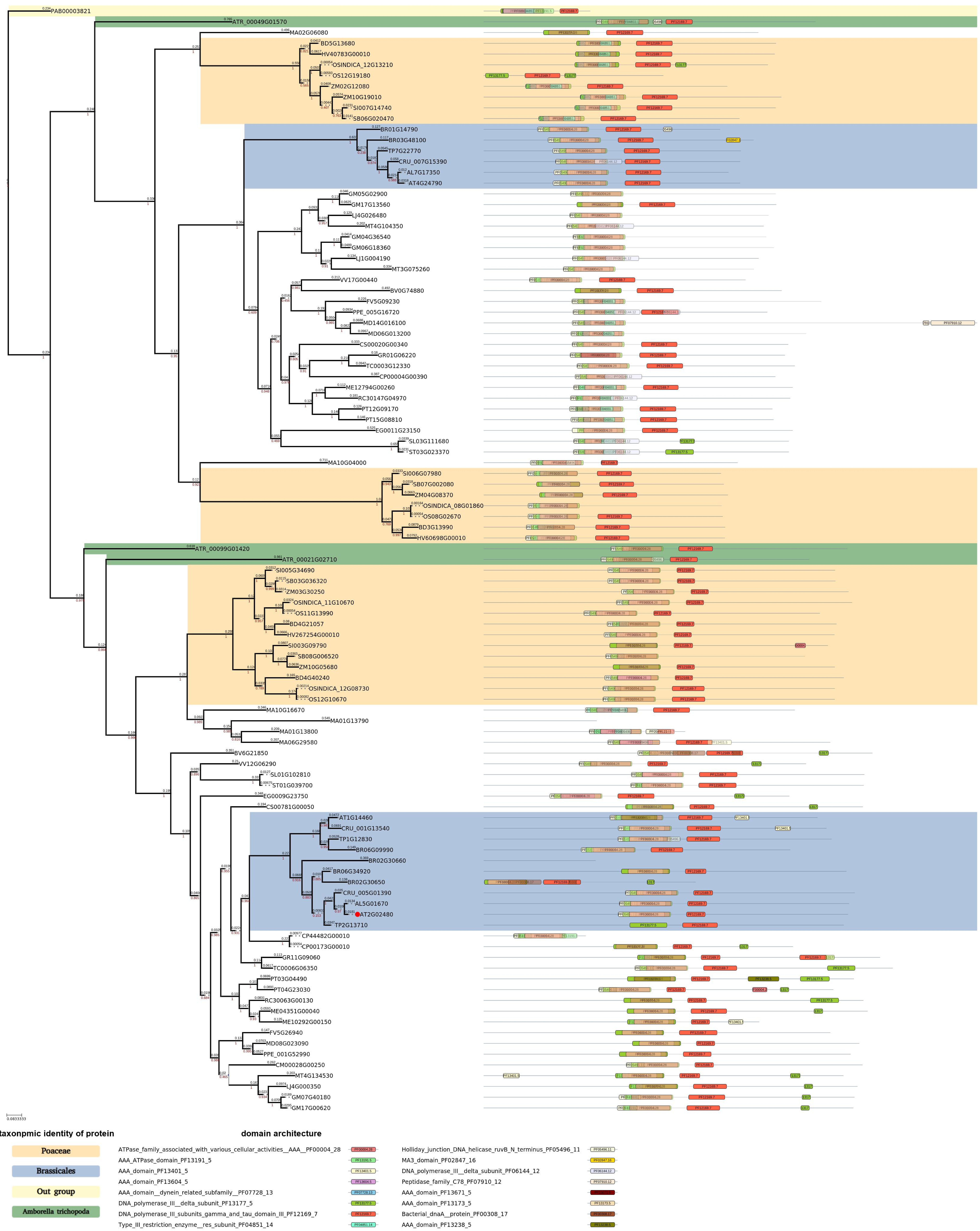

Figure S26. PhyML phylogenetic relations and composition of domains for AT2G22640 homologues of representative plant species. The color legends of species and domains is given below

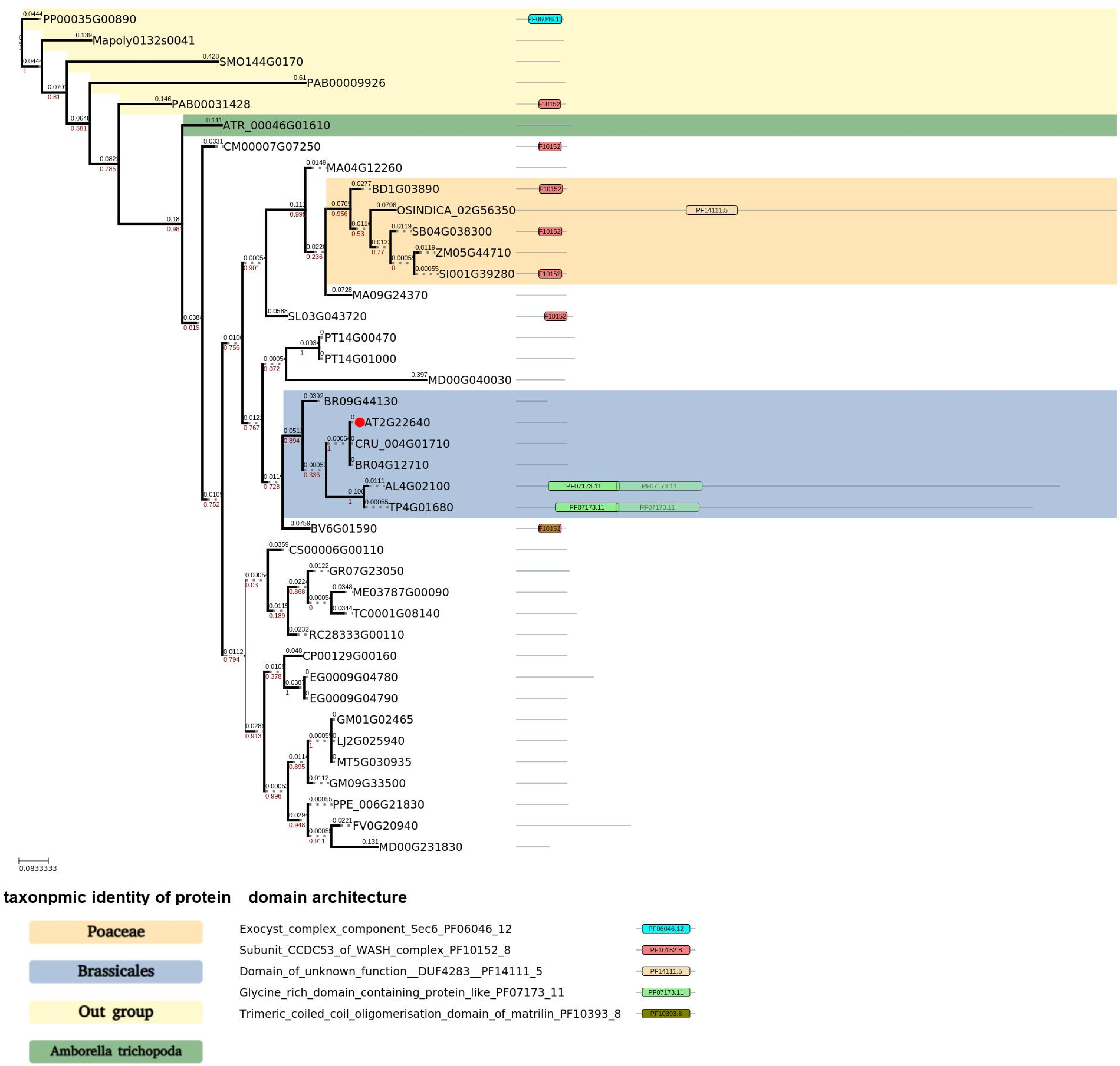

Figure S27. PhyML phylogenetic relations and composition of domains for AT2G27300 homologues of representative plant species.
The color legends of species and domains is given below

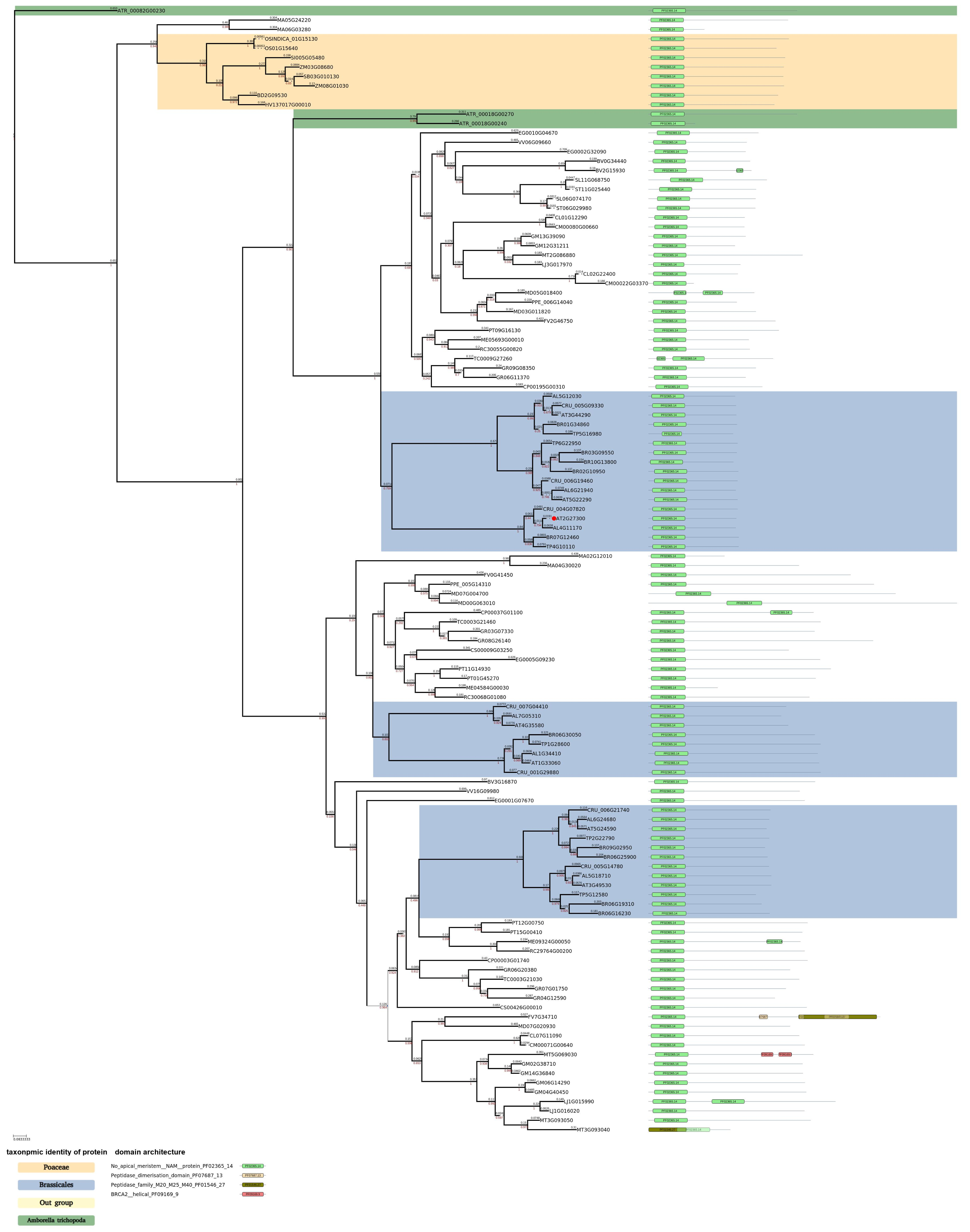

Figure S28. PhyML phylogenetic relations and composition of domains for AT2G27600 homologues of representative plant species. The color legends of species and domains is given below

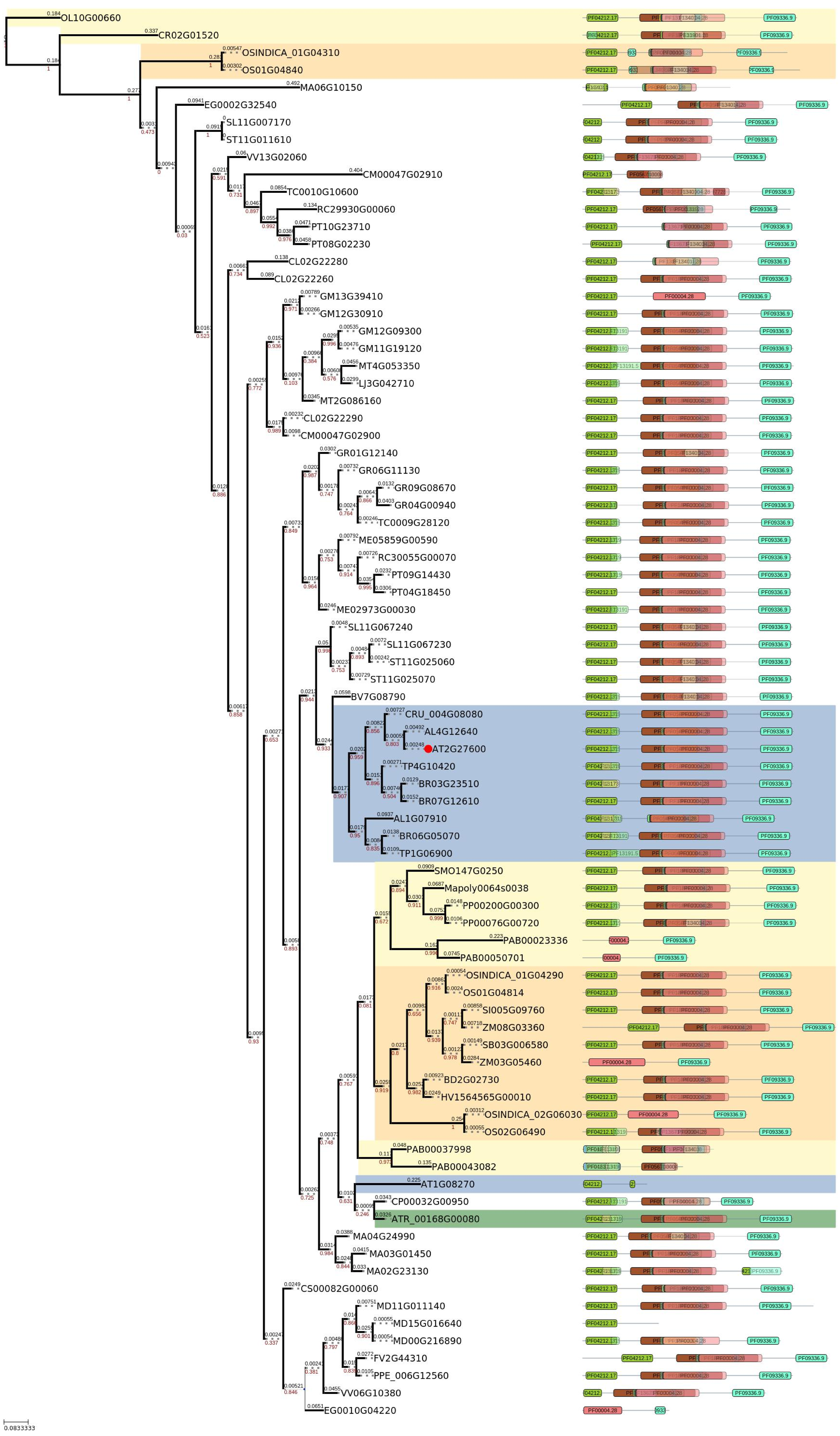

taxonpmic identity of protein

Poaceae
Brassicales
Out group
Amborella trichopoda

domain architecture

ATPase_family_associated_with_various_cellular_activities__AAA__PF00004_28	PF00004.28	IstB_like_ATP_binding_protein_PF01695_16	PF01695.16
AAA_domain_PF13173_5	PF13173.5	MIT__microtubule_interacting_and_transport__domain_PF04212_17	PF04212.17
AAA_ATPase_domain_PF13191_5	PF13191.5	Holliday_junction_DNA_helicase_ruvB_N_terminus_PF05496_11	PF05496.11
AAA_domain__Cdc48_subfamily__PF07724_13	PF07724.13	TIP49_C_terminus_PF06068_12	PF06068.12
AAA_domain_PF13401_5	PF13401.5	Vps4_C_terminal_oligomerisation_domain_PF09336_9	PF09336.9
AAA_domain_PF13671_5	PF13671.5	AAA_domain_PF13238_5	PF13238.5
AAA_domain__dynein_related_subfamily__PF07728_13		Parvovirus_non_structural_protein_NS1_PF01057_16	PF01057.16
Protein_of_unknown_function__DUF815__PF05673_12	PF05673.12	ATPase_domain_predominantly_from_Archaea_PF01637_17	PF01637.17

Figure S29. PhyML phylogenetic relations and composition of domains for AT2G31300 homologues of representative plant species. The color legends of species and domains is given below

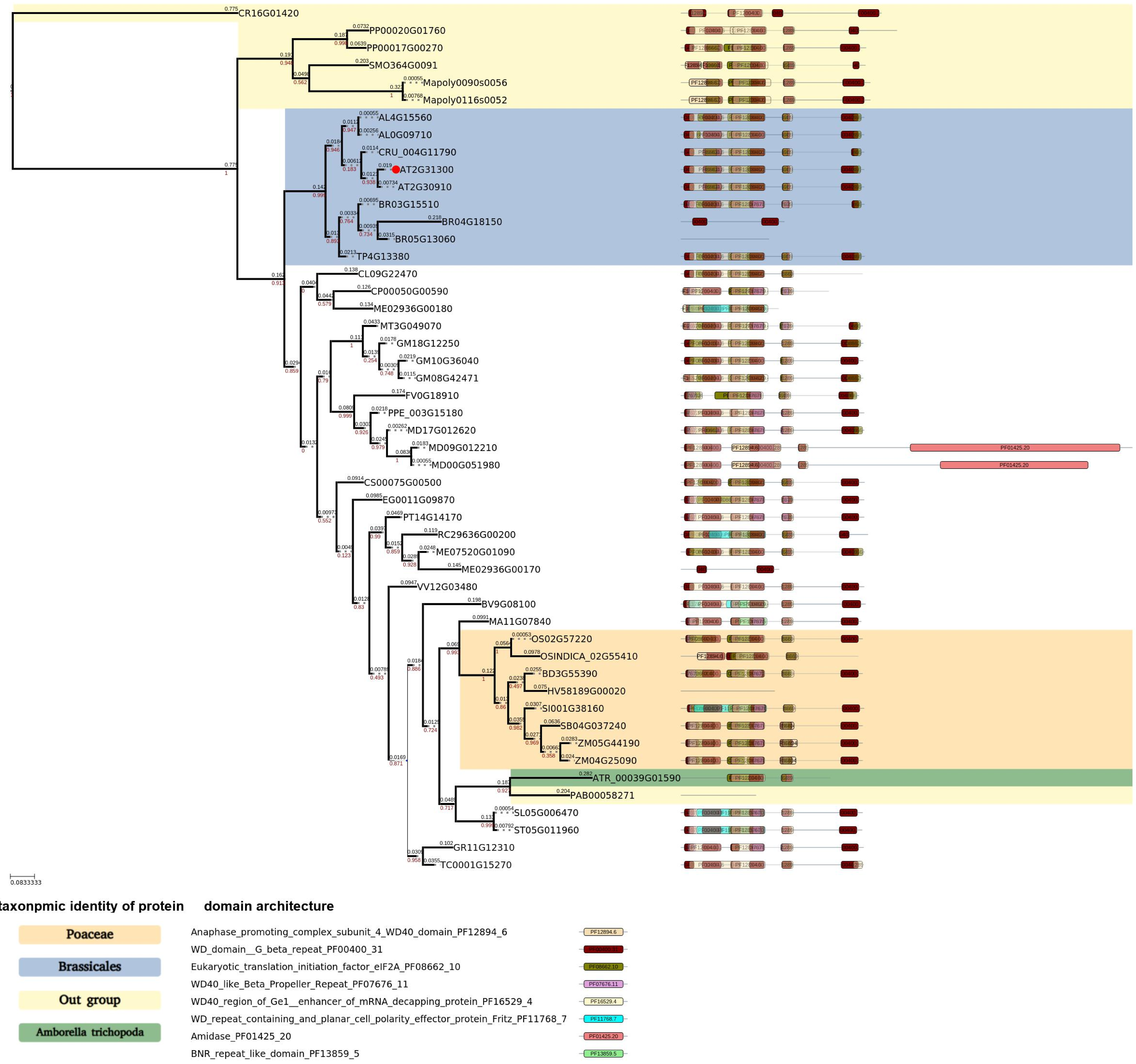

Figure S30. PhyML phylogenetic relations and composition of domains for AT2G33540 homologues of representative plant species. The color legends of species and domains is given below

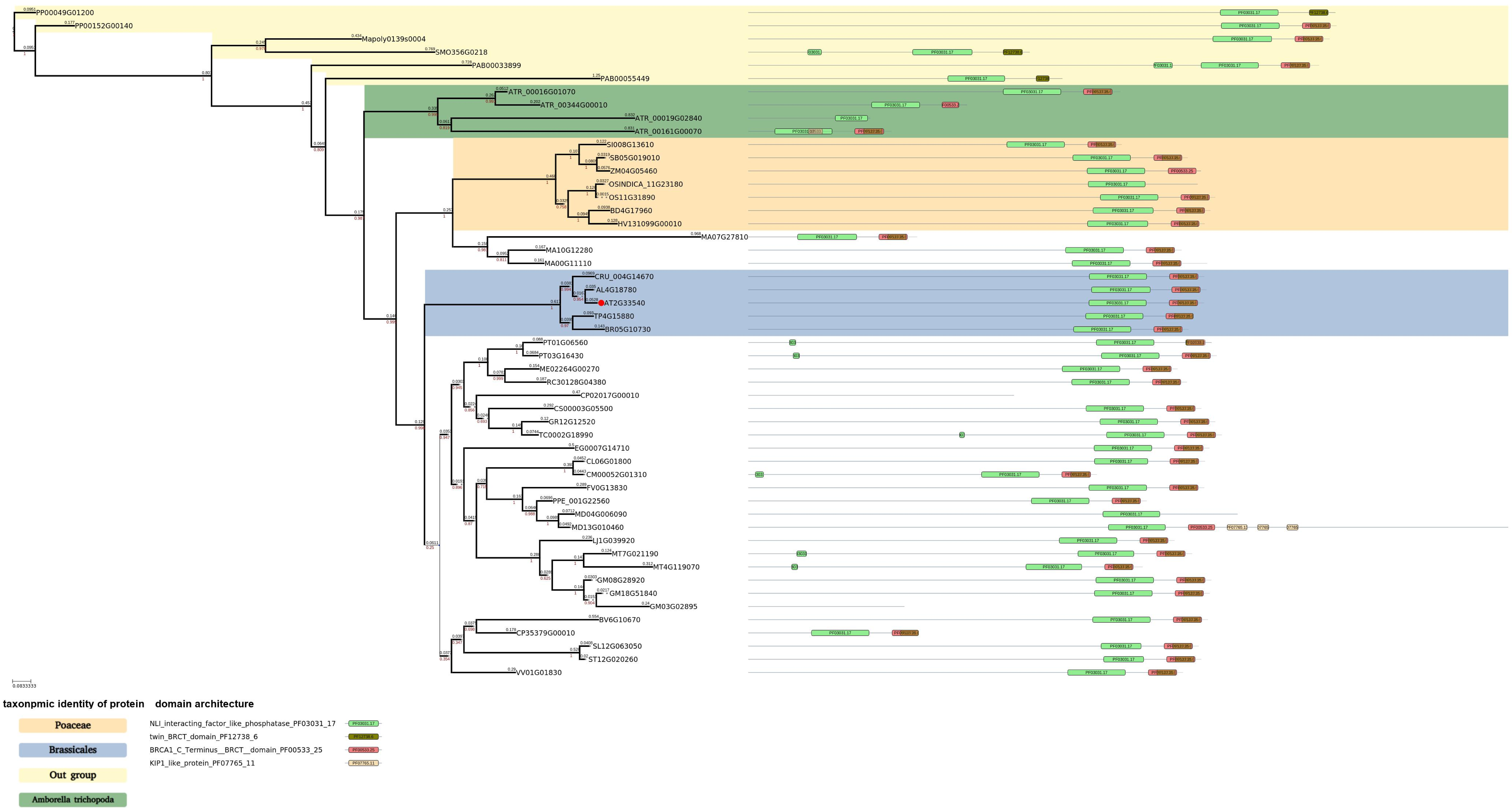

Figure S31. PhyML phylogenetic relations and composition of domains for AT2G35110 homologues of representative plant species. The color legends of species and domains is given below

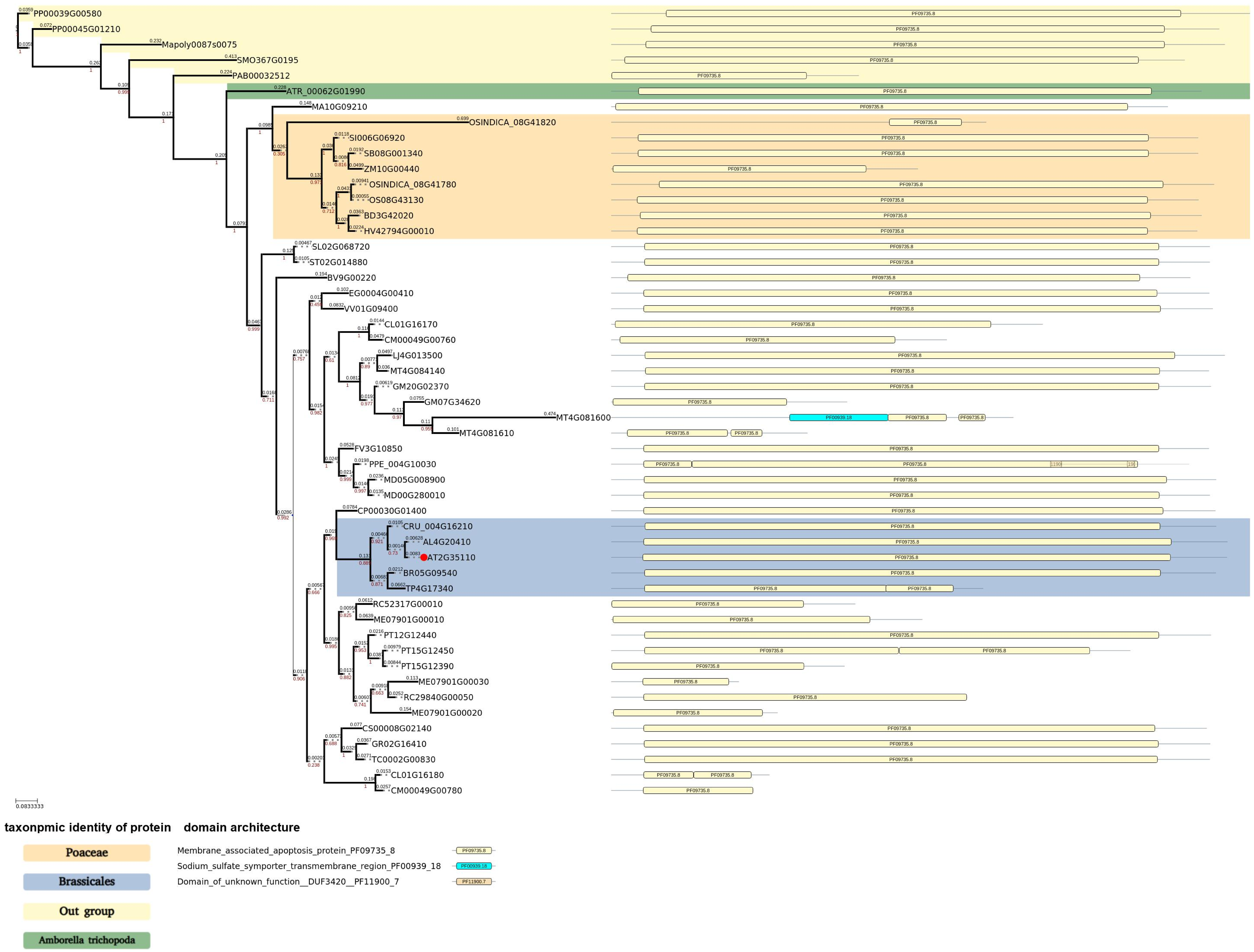

Figure S32. PhyML phylogenetic relations and composition of domains for AT2G39940 homologues of representative plant species. The color legends of species and domains is given below

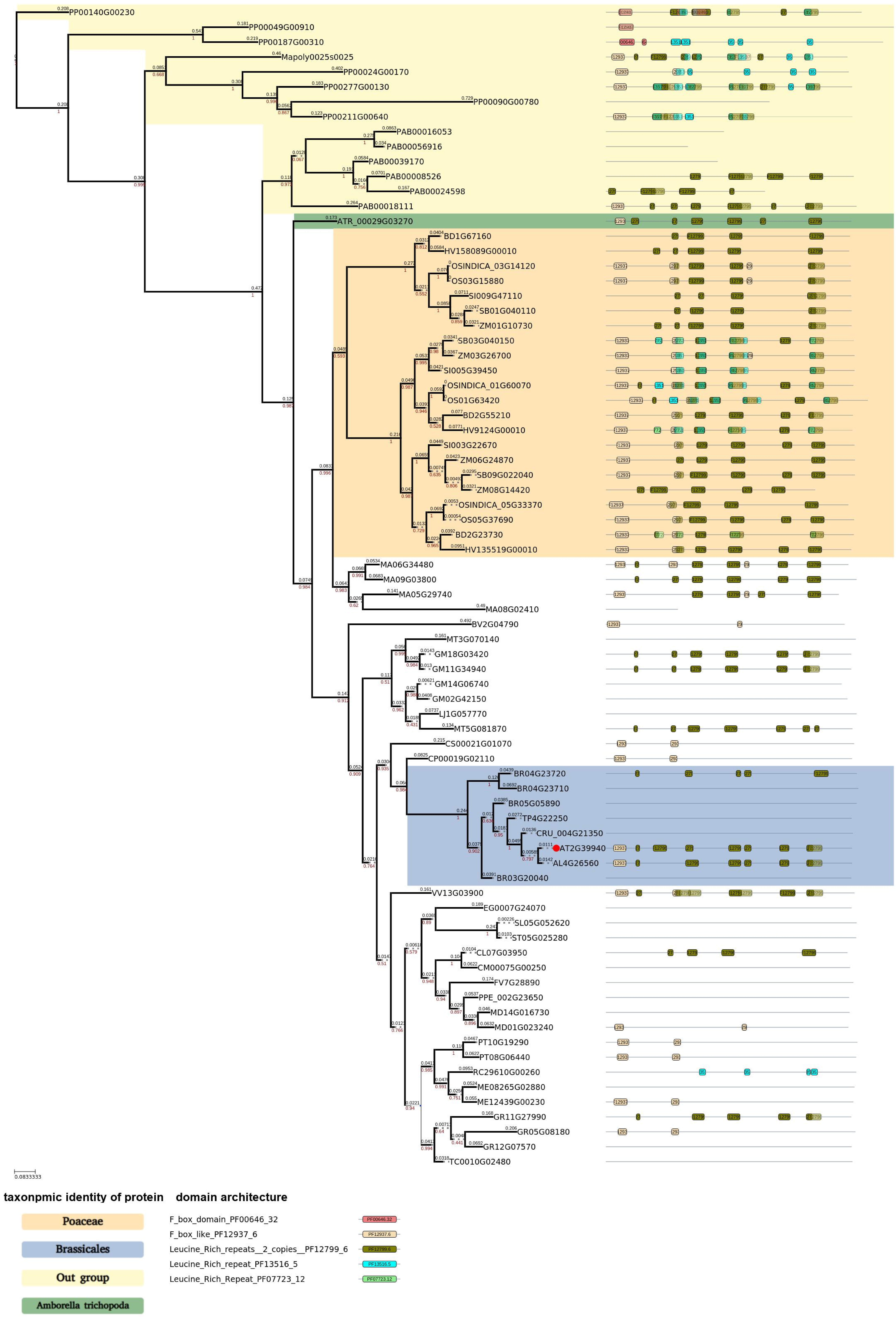

Figure S33. PhyML phylogenetic relations and composition of domains for AT2G42260 homologues of representative plant species. The color legends of species and domains is given below

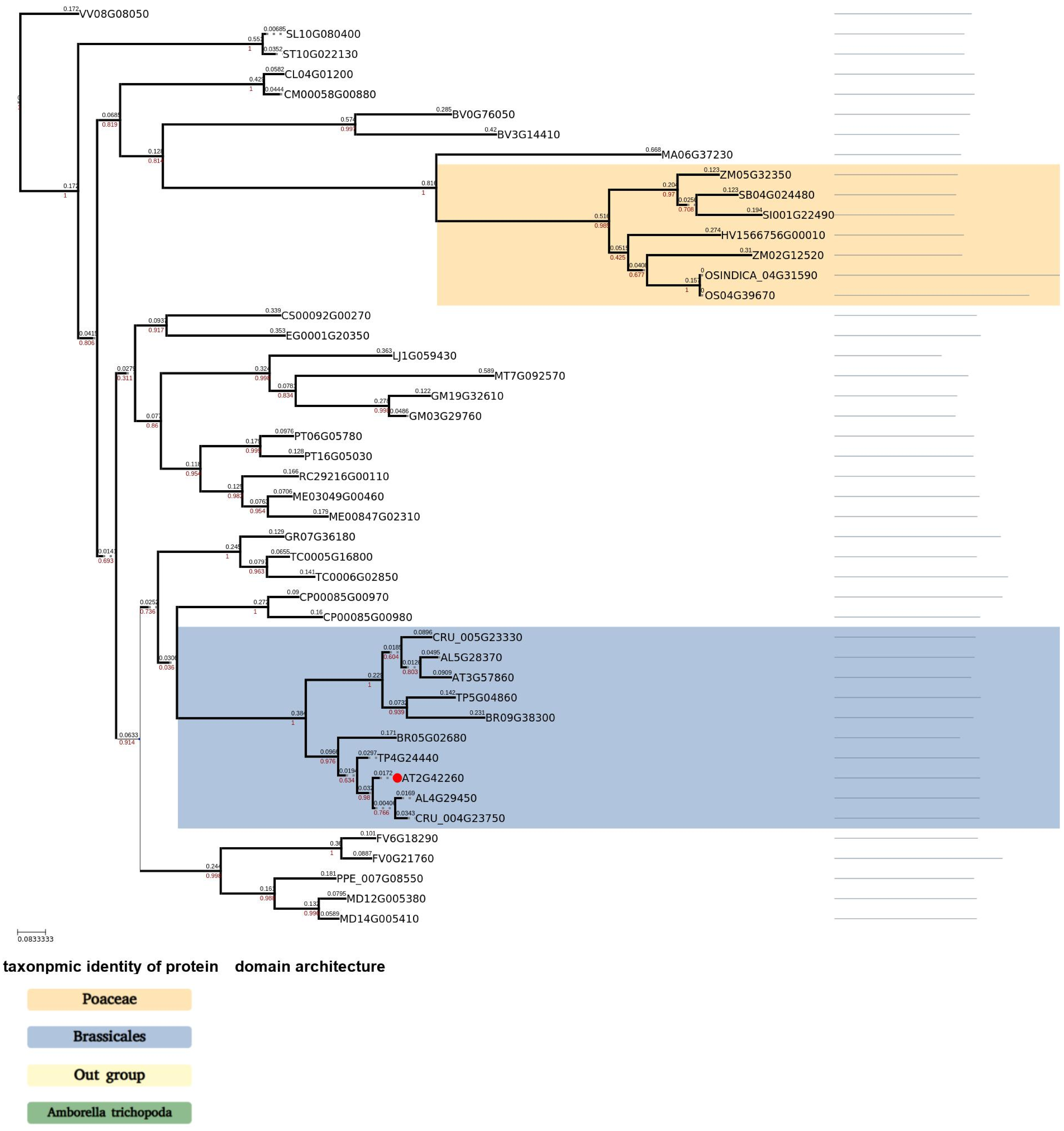

Figure S34. PhyML phylogenetic relations and composition of domains for AT2G46225, AT5G42030, AT5G24310 homologues of representative plant species. The color legends of species and domains is given below

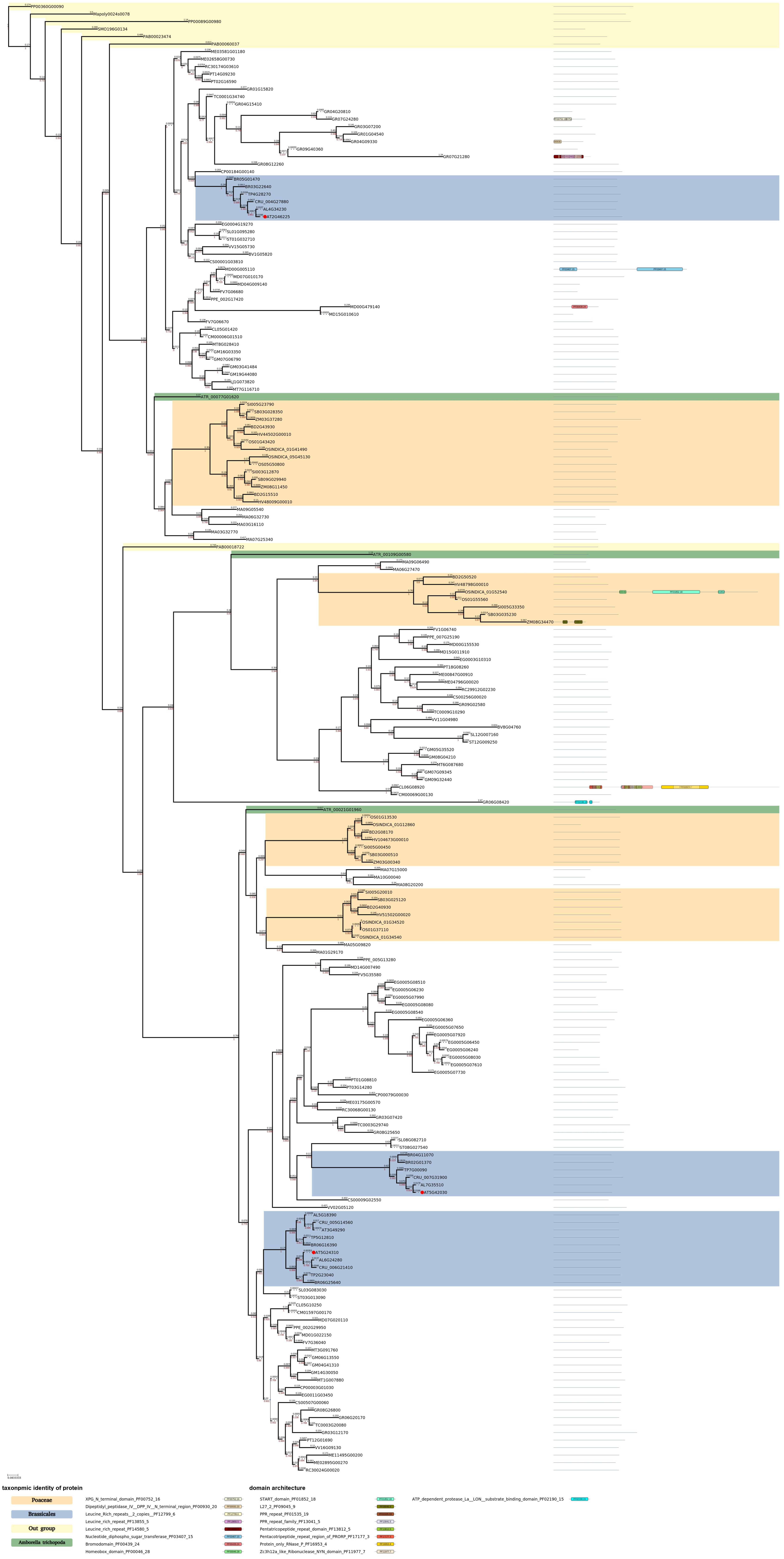

Supplement: Supplementary file 9 — Figure S25: PhyML phylogenetic relations and composition of domains for AT2G02480 homologues of representative plant species. Figure S26: PhyML phylogenetic relations and composition of domains for AT2G22640 homologues of representative plant species. Figure S27: PhyML phylogenetic relations and composition of domains for AT2G27300 homologues of representative plant species. Figure S28: PhyML phylogenetic relations and composition of domains for AT2G27600 homologues of representative plant species. Figure S29: PhyML phylogenetic relations and composition of domains for AT2G31300 homologues of representative plant species. Figure S30: PhyML phylogenetic relations and composition of domains for AT2G33540 homologues of representative plant species. Figure S31: PhyML phylogenetic relations and composition of domains for AT2G35110 homologues of representative plant species. Figure S32: PhyML phylogenetic relations and composition of domains for AT2G39940 homologues of representative plant species. Figure S33: PhyML phylogenetic relations and composition of domains for AT2G42260 homologues of representative plant species. Figure S34: PhyML phylogenetic relations and composition of domains for AT2G46225; AT5G42030; AT5G24310 homologues of representative plant species. (PDF 6990 kb) [file 12870_2019_1640_MOESM9_ESM.pdf]
