# Supplementary material for: The evolution of gene regulatory networks controlling Arabidopsis thaliana L. trichome development
Source: BMC Plant Biol. 2019 Feb 15;19(Suppl 1):53. doi: 10.1186/s12870-019-1640-2 (PMC6393967; doi:10.1186/s12870-019-1640-2)

Figure S35. PhyML phylogenetic relations and composition of domains for AT2G46600 homologues of representative plant species. The color legends of species and domains is given below

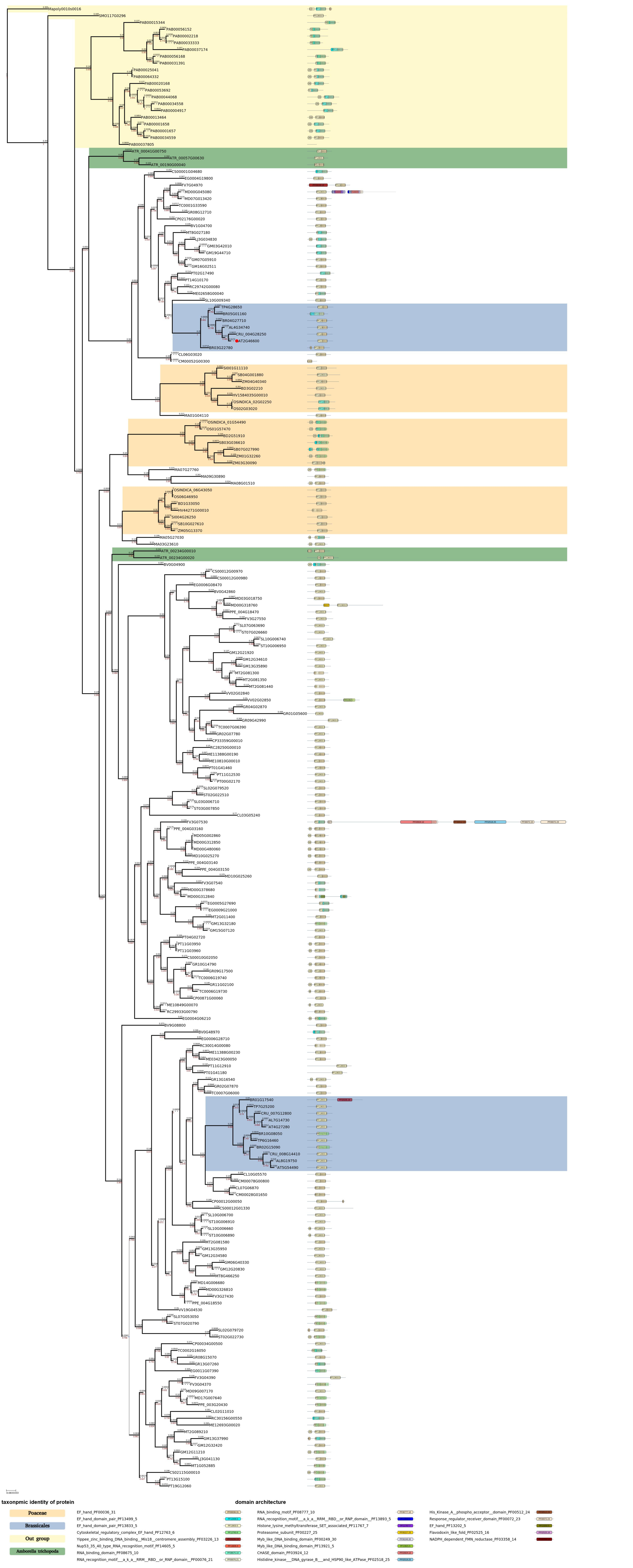

Figure S36. PhyML phylogenetic relations and composition of domains for AT3G12280 homologues of representative plant species. The color legends of species and domains is given below

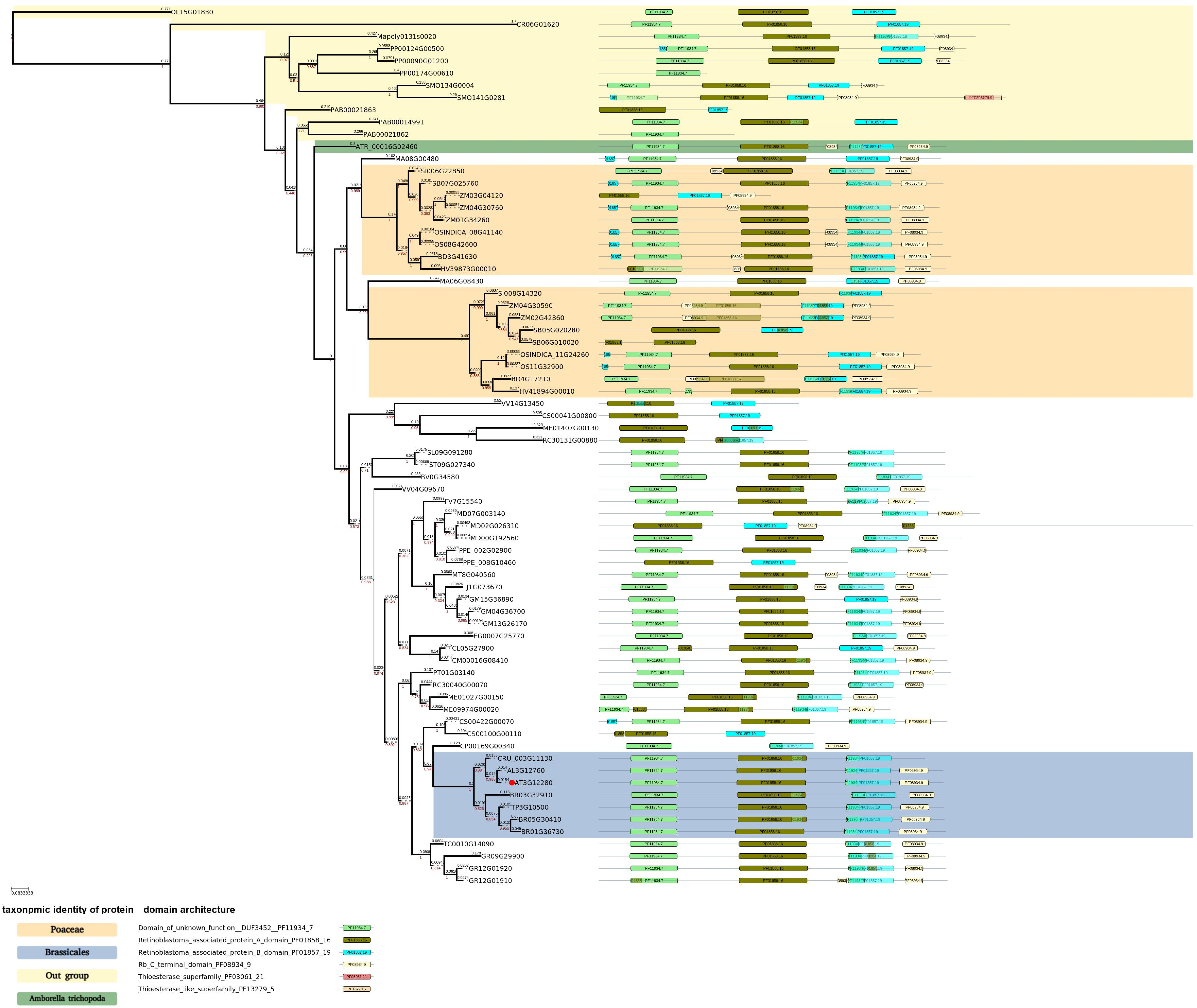

Figure S37. PhyML phylogenetic relations and composition of domains for AT3G12400 homologues of representative plant species. The color legends of species and domains is given below

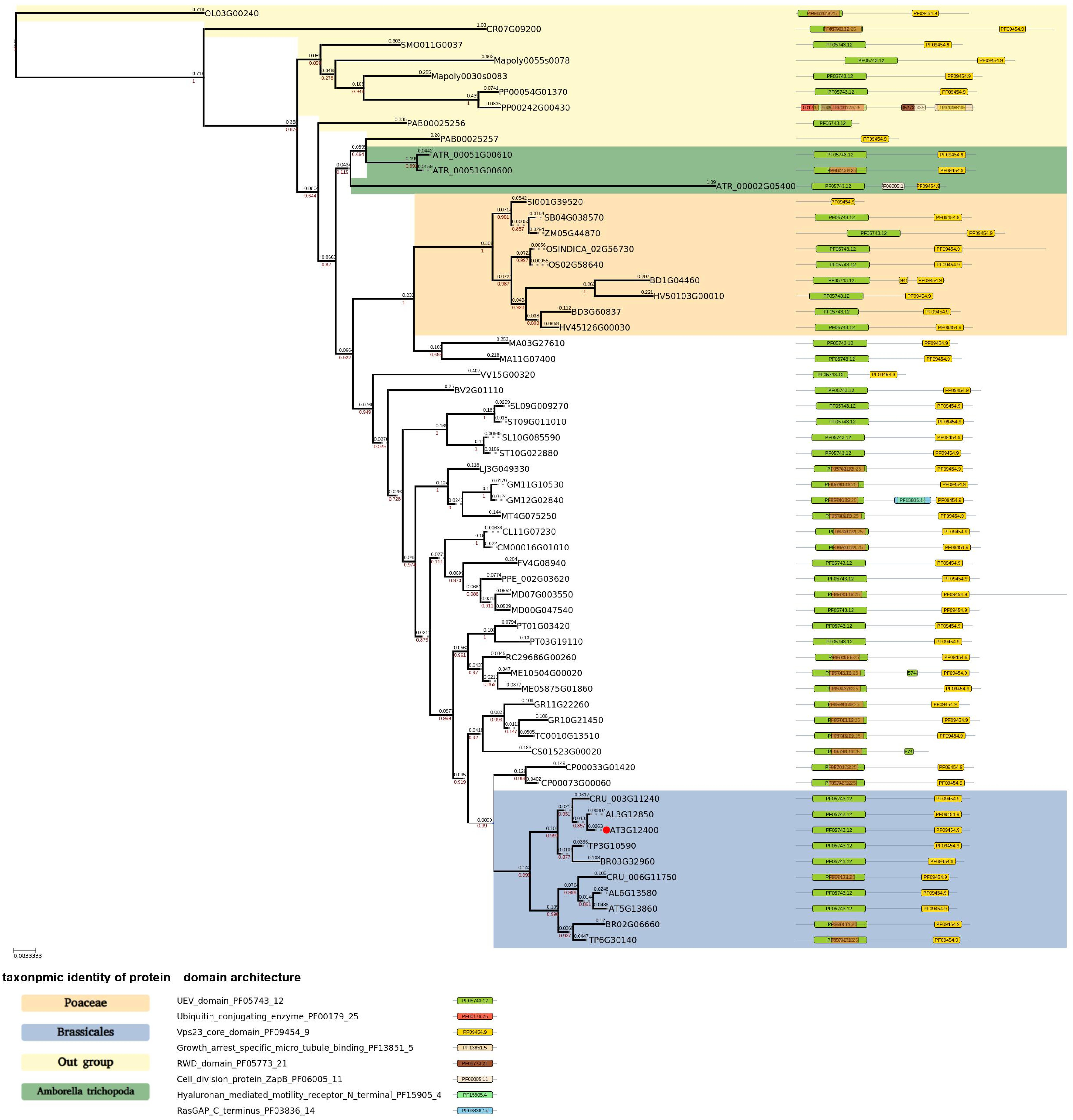

Figure S38. PhyML phylogenetic relations and composition of domains for AT3G16630 homologues of representative plant species. The color legends of species and domains is given below

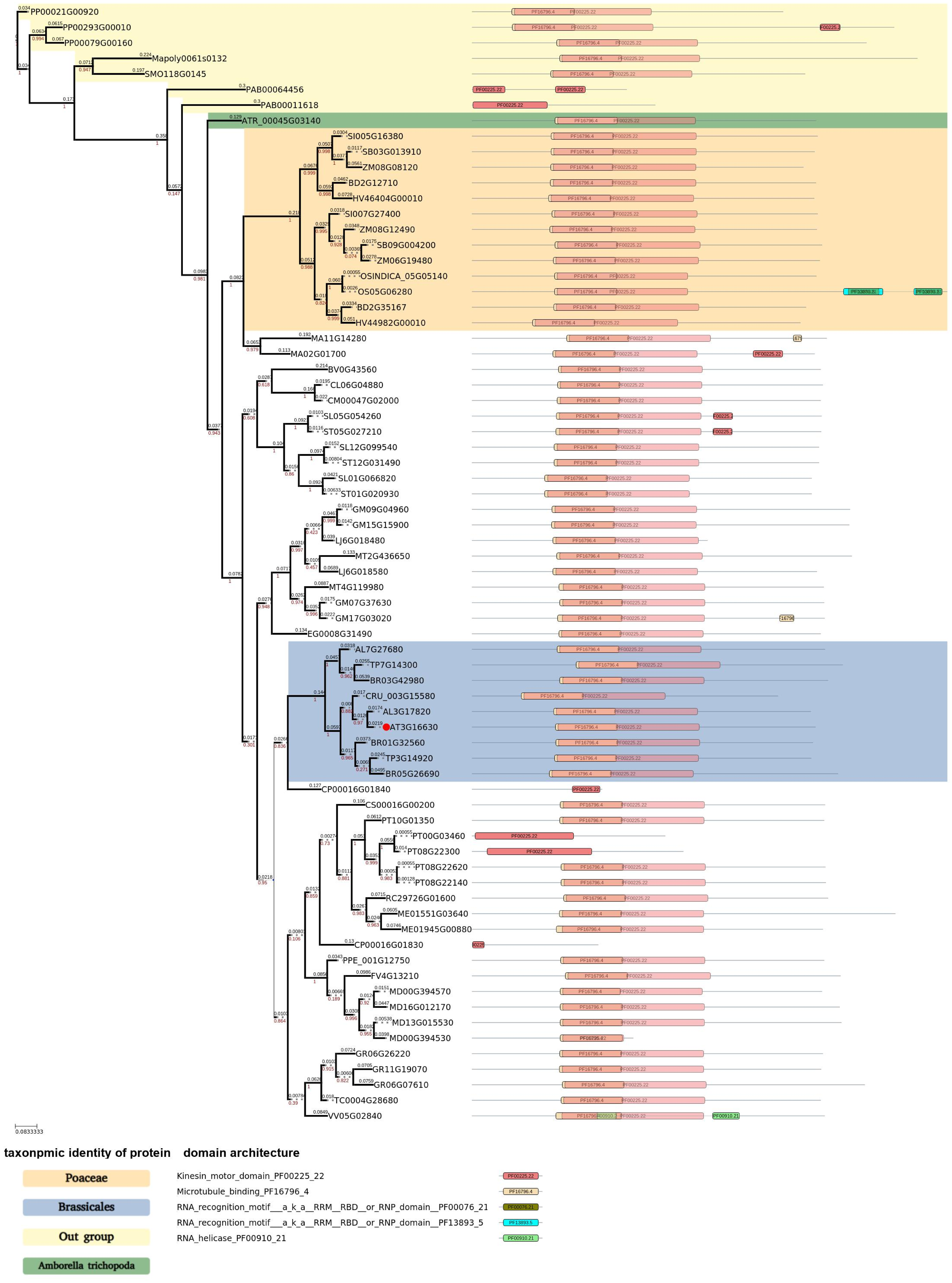

Figure S39. PhyML phylogenetic relations and composition of domains for AT3G27000 homologues of representative plant species. The color legends of species and domains is given below

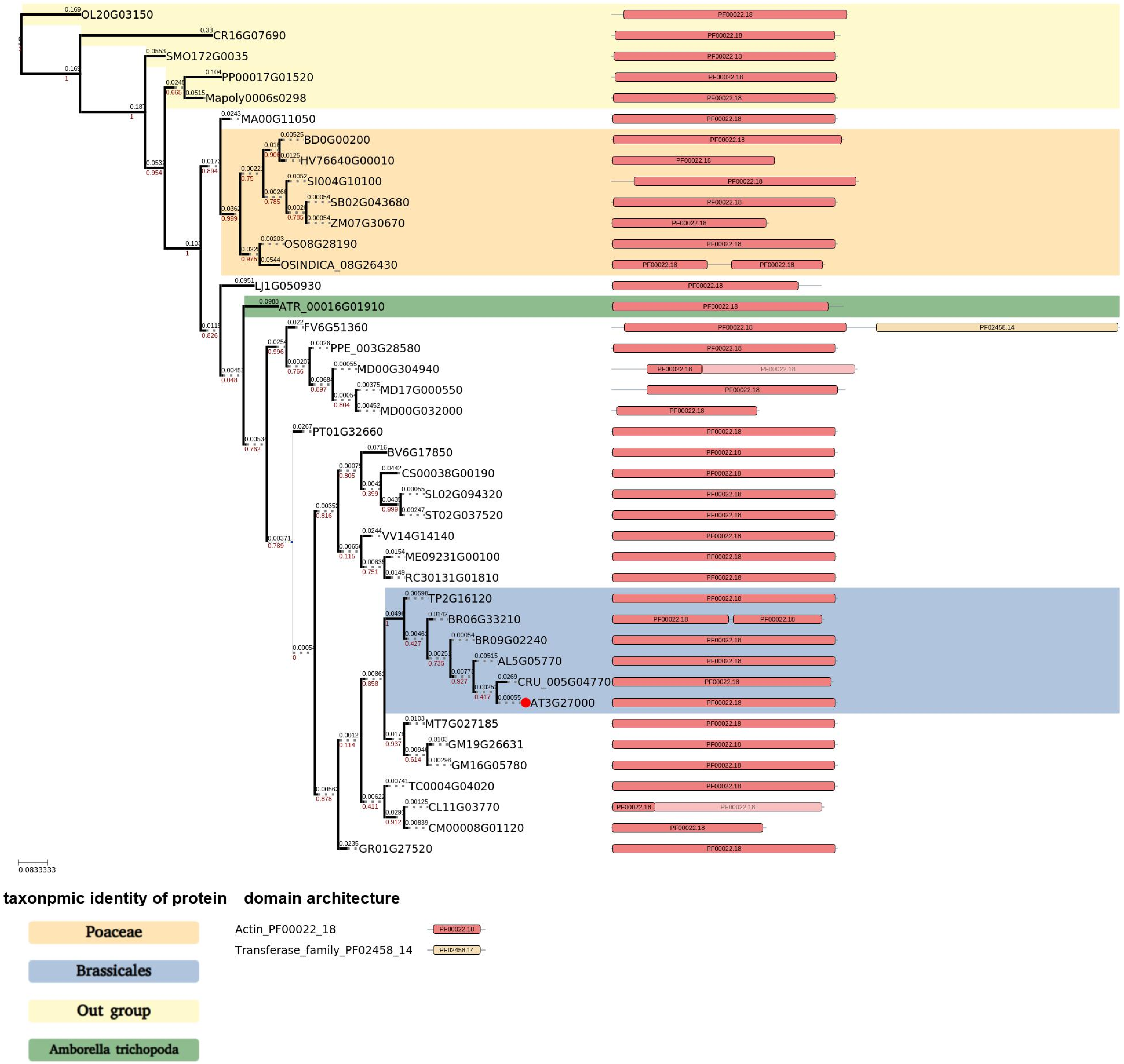

Figure S40. PhyML phylogenetic relations and composition of domains for AT3G50530 homologues of representative plant species. The color legends of species and domains is given below

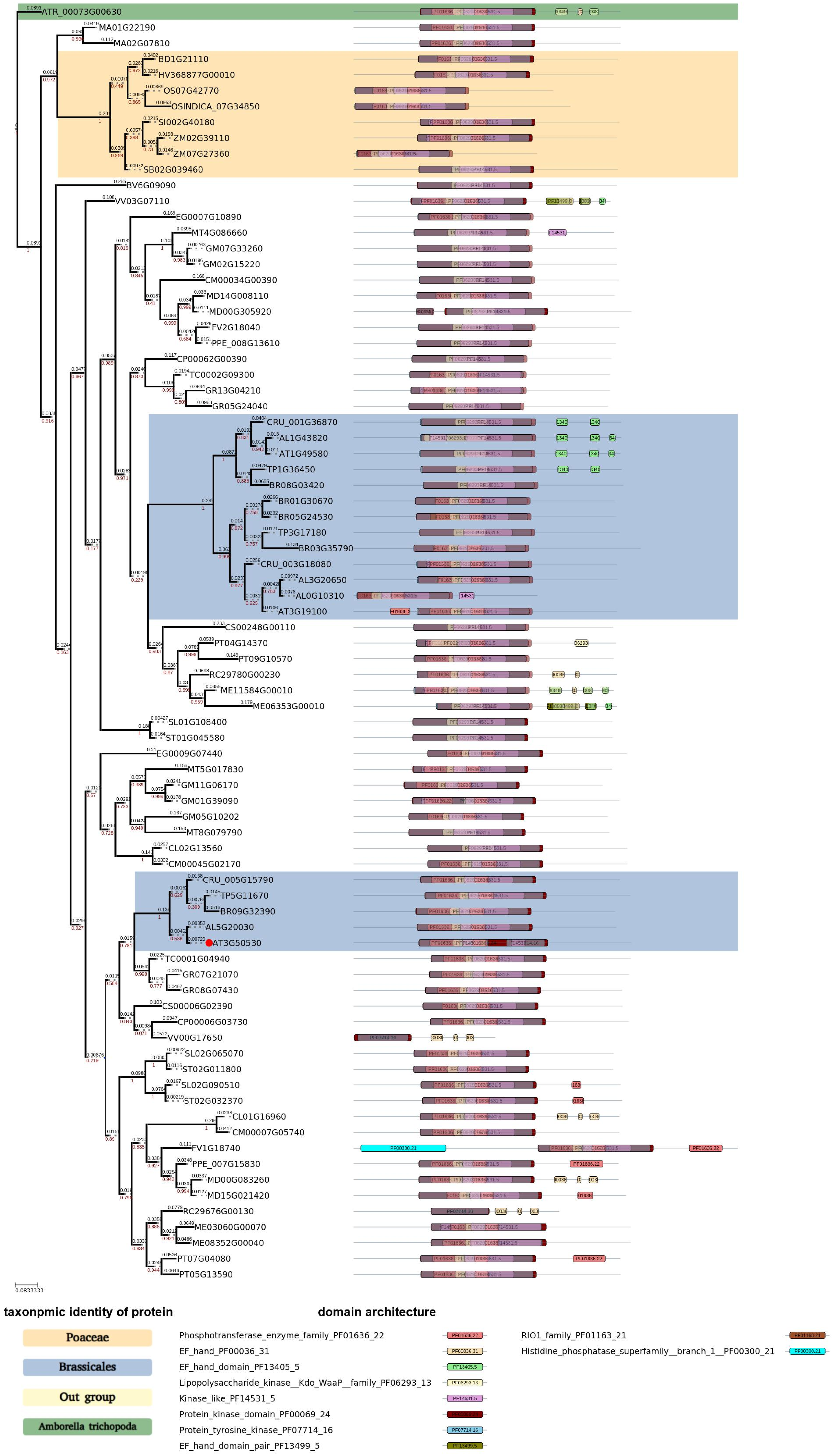

Figure S41. PhyML phylogenetic relations and composition of domains for AT5G27320, AT3G05120, AT3G63010 homologues of representative plant species. The color legends of species and domains is given below

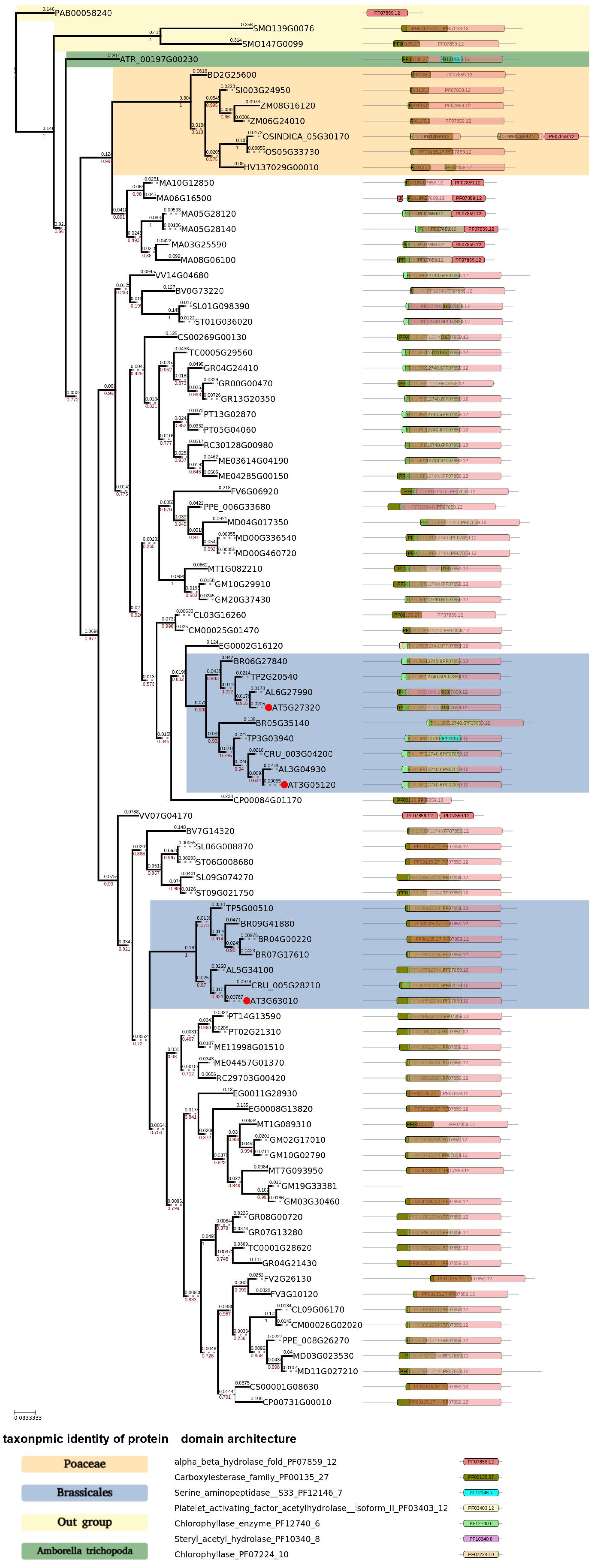

Figure S42. PhyML phylogenetic relations and composition of domains for AT4G01710, AT5G65274 homologues of representative plant species. The color legends of species and domains is given below

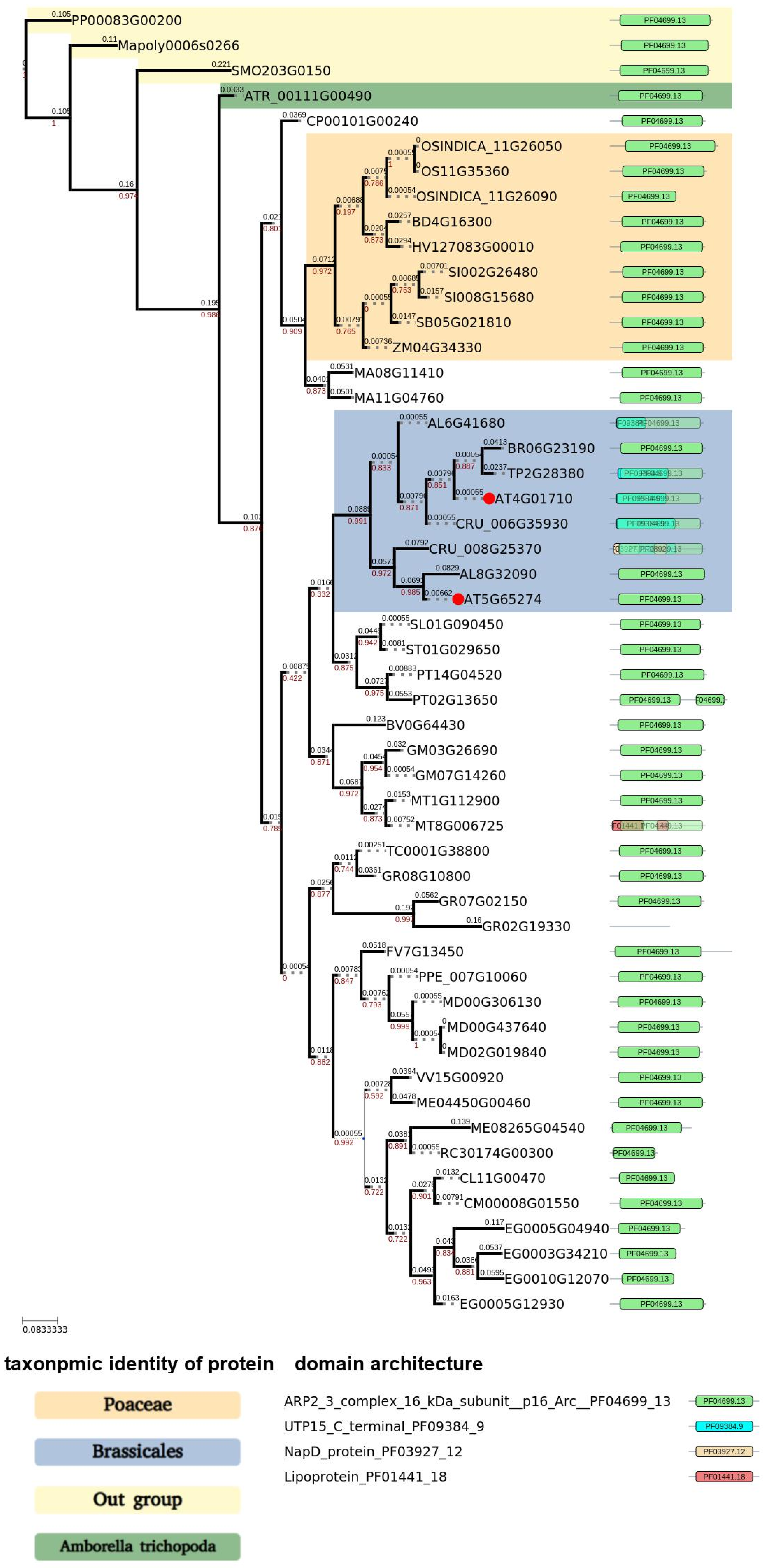

Figure S43. PhyML phylogenetic relations and composition of domains for AT4G02570 homologues of representative plant species.
The color legends of species and domains is given below

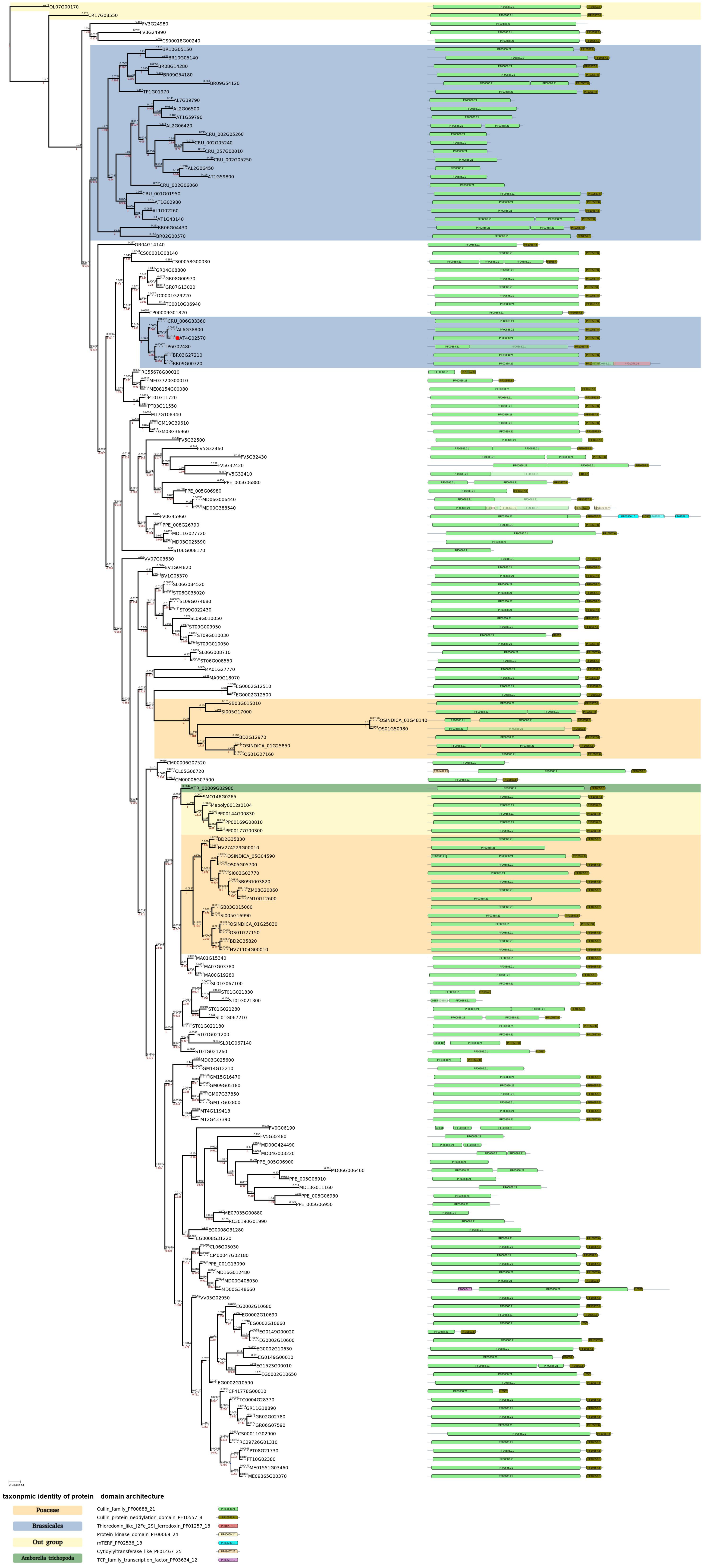

Figure S44. PhyML phylogenetic relations and composition of domains for AT4G12610 homologues of representative plant species. The color legends of species and domains is given below

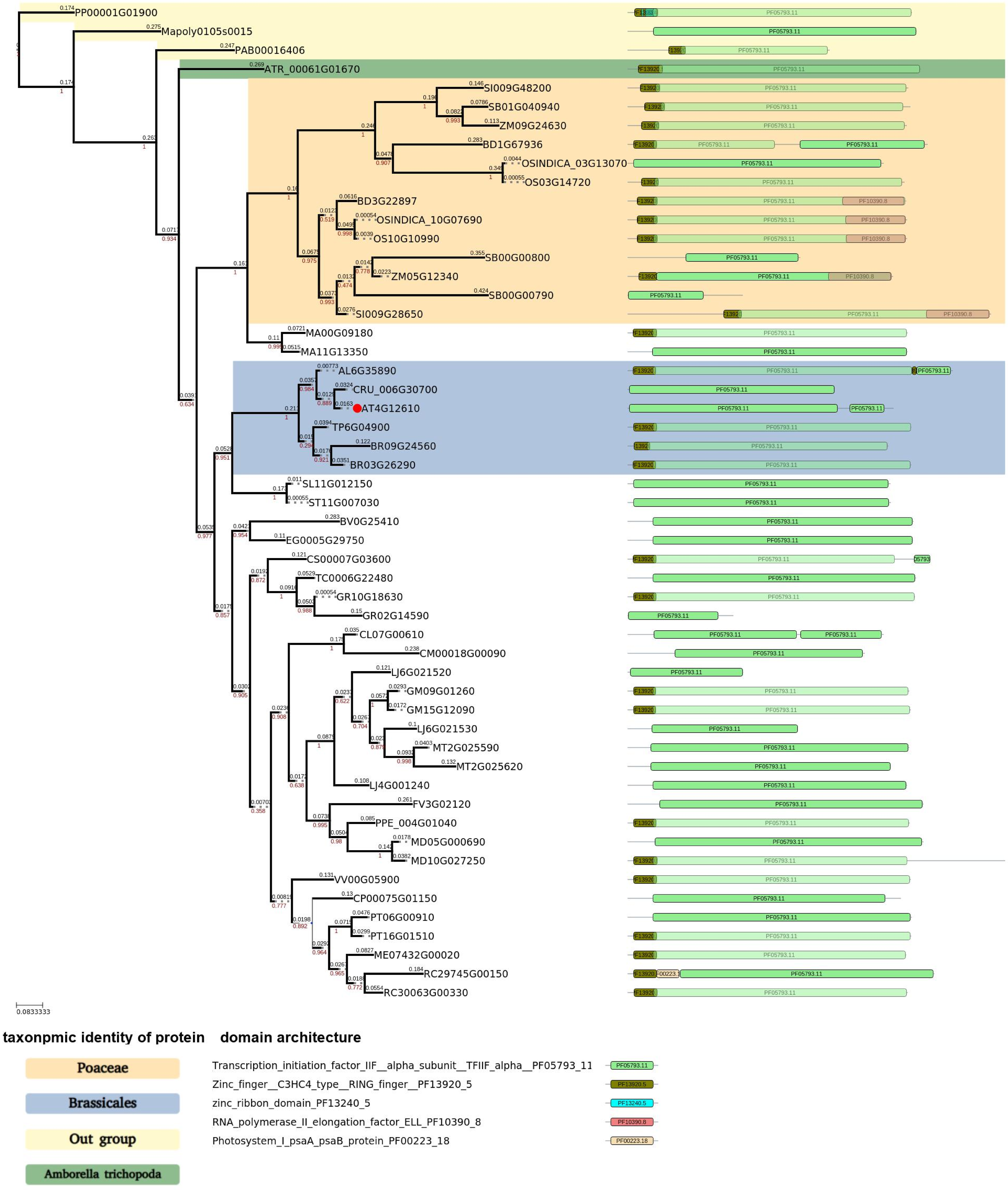

Supplement: Supplementary file 10 — Figure S35: PhyML phylogenetic relations and composition of domains for AT2G46600 homologues of representative plant species. Figure S36: PhyML phylogenetic relations and composition of domains for AT3G12280 homologues of representative plant species. Figure S37: PhyML phylogenetic relations and composition of domains for AT3G12400 homologues of representative plant species. Figure S38: PhyML phylogenetic relations and composition of domains for AT3G16630 homologues of representative plant species. Figure S39: PhyML phylogenetic relations and composition of domains for AT3G27000 homologues of representative plant species. Figure S40: PhyML phylogenetic relations and composition of domains for AT3G50530 homologues of representative plant species. Figure S41: PhyML phylogenetic relations and composition of domains for AT5G27320; AT3G05120; AT3G63010 homologues of representative plant species. Figure S42: PhyML phylogenetic relations and composition of domains for AT4G01710; AT5G65274 homologues of representative plant species. Figure S43: PhyML phylogenetic relations and composition of domains for AT4G02570 homologues of representative plant species. Figure S44: PhyML phylogenetic relations and composition of domains for AT4G12610 homologues of representative plant species. (PDF 7692 kb) [file 12870_2019_1640_MOESM10_ESM.pdf]
