# Supplementary material for: The evolution of gene regulatory networks controlling Arabidopsis thaliana L. trichome development
Source: BMC Plant Biol. 2019 Feb 15;19(Suppl 1):53. doi: 10.1186/s12870-019-1640-2 (PMC6393967; doi:10.1186/s12870-019-1640-2)

Figure S45. PhyML phylogenetic relations and composition of domains for AT4G14147 homologues of representative plant species. The color legends of species and domains is given below

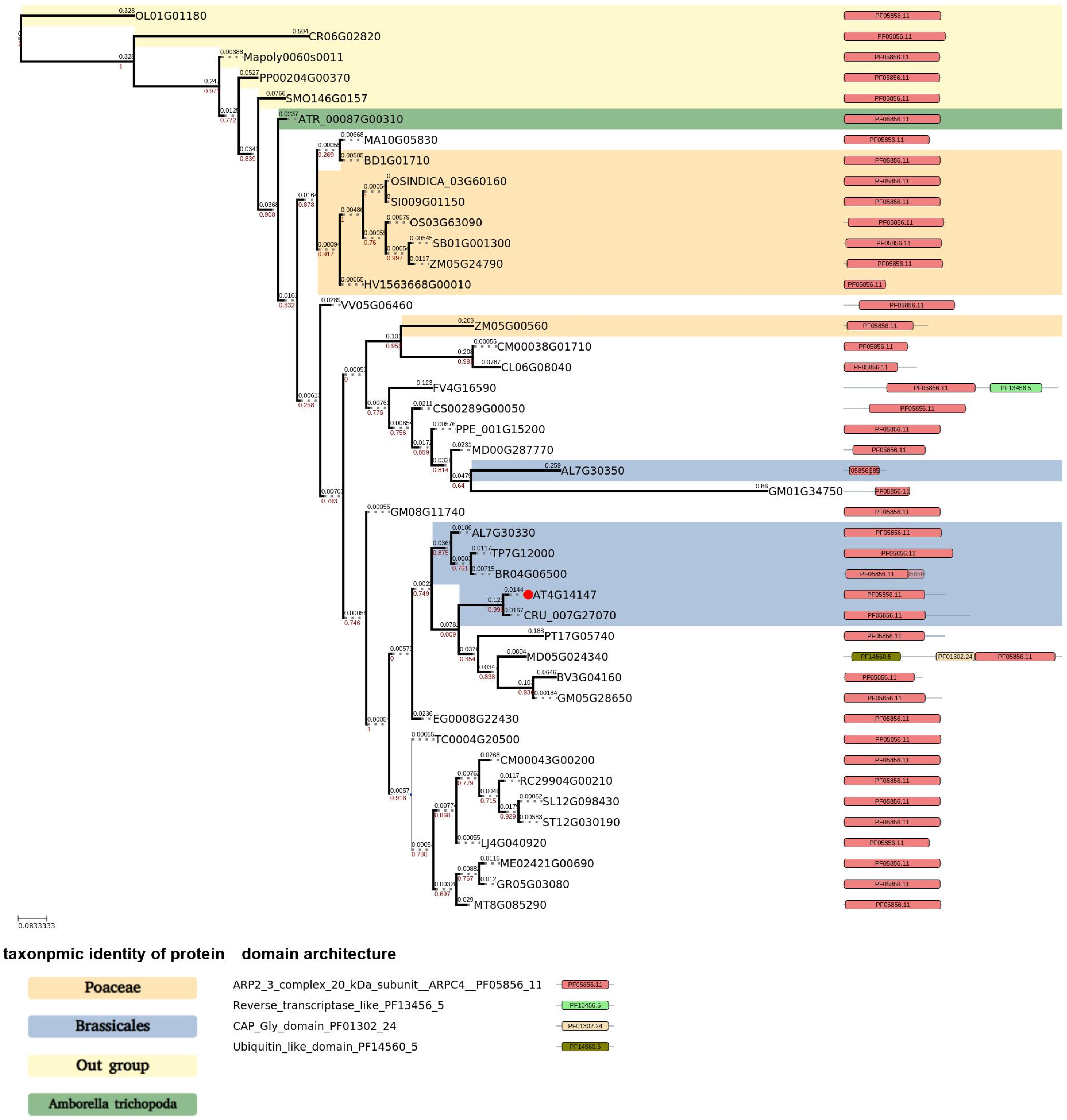

Figure S46. PhyML phylogenetic relations and composition of domains for AT4G15415 homologues of representative plant species. The color legends of species and domains is given below

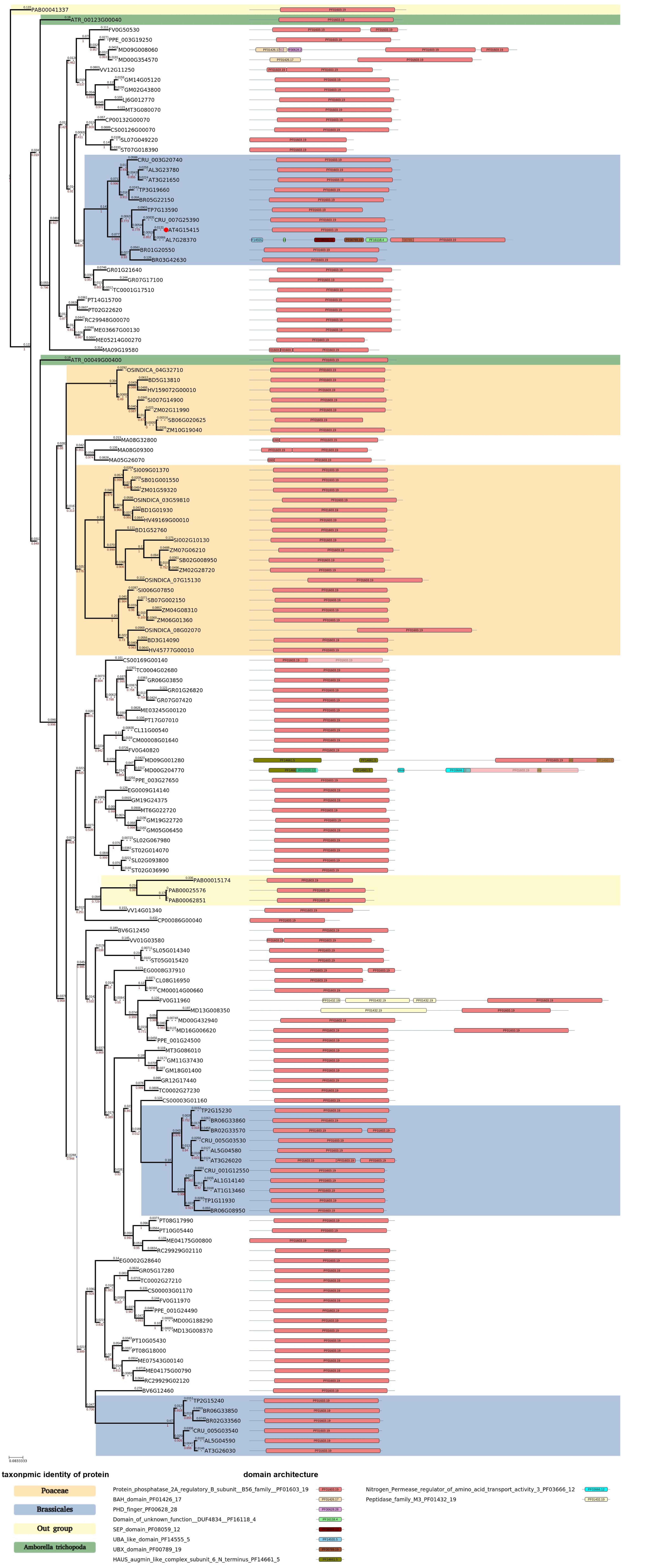

Figure S47. PhyML phylogenetic relations and composition of domains for AT4G20780 homologues of representative plant species. The color legends of species and domains is given below

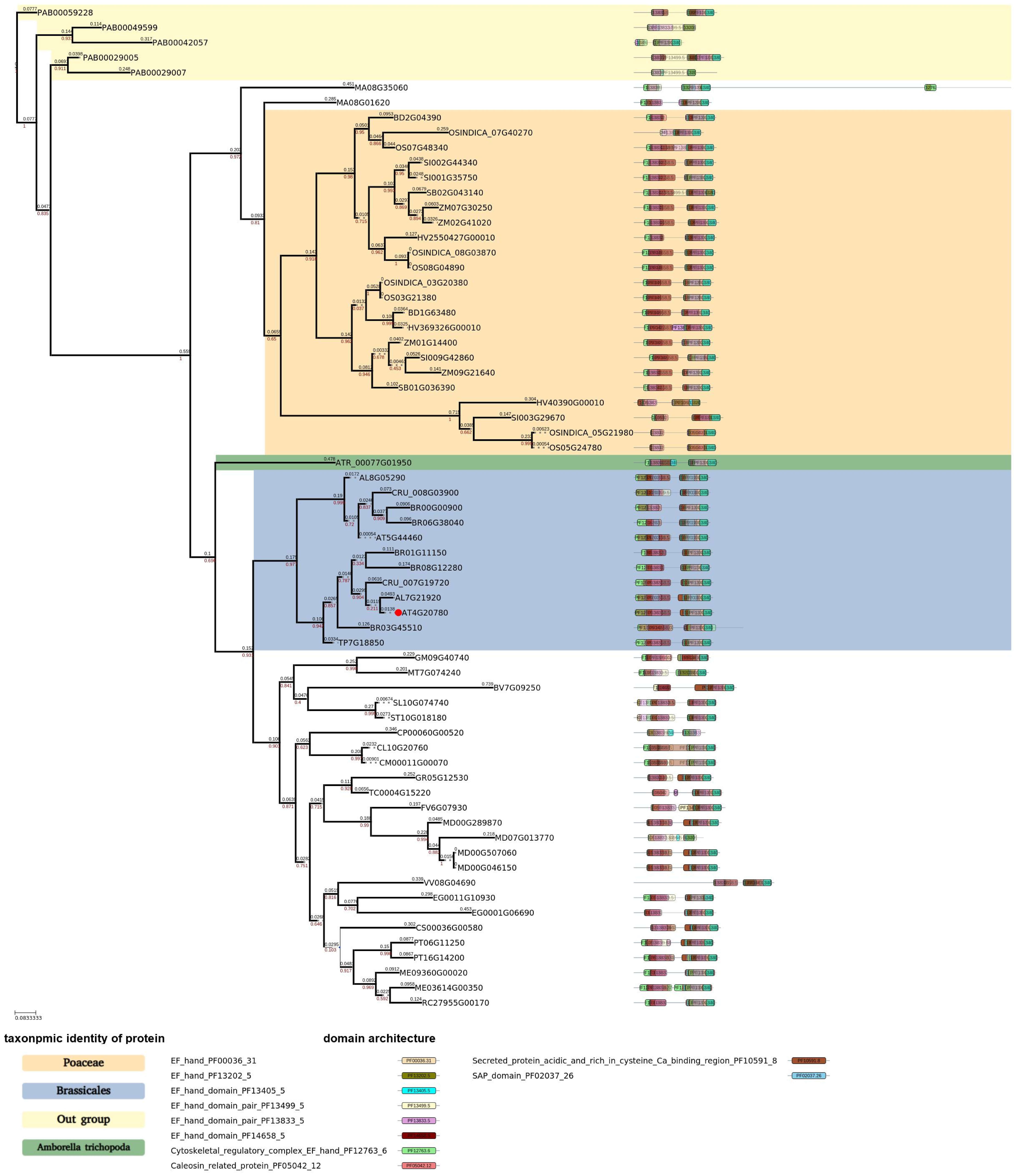

Figure S48. PhyML phylogenetic relations and composition of domains for AT4G22910 homologues of representative plant species. The color legends of species and domains is given below

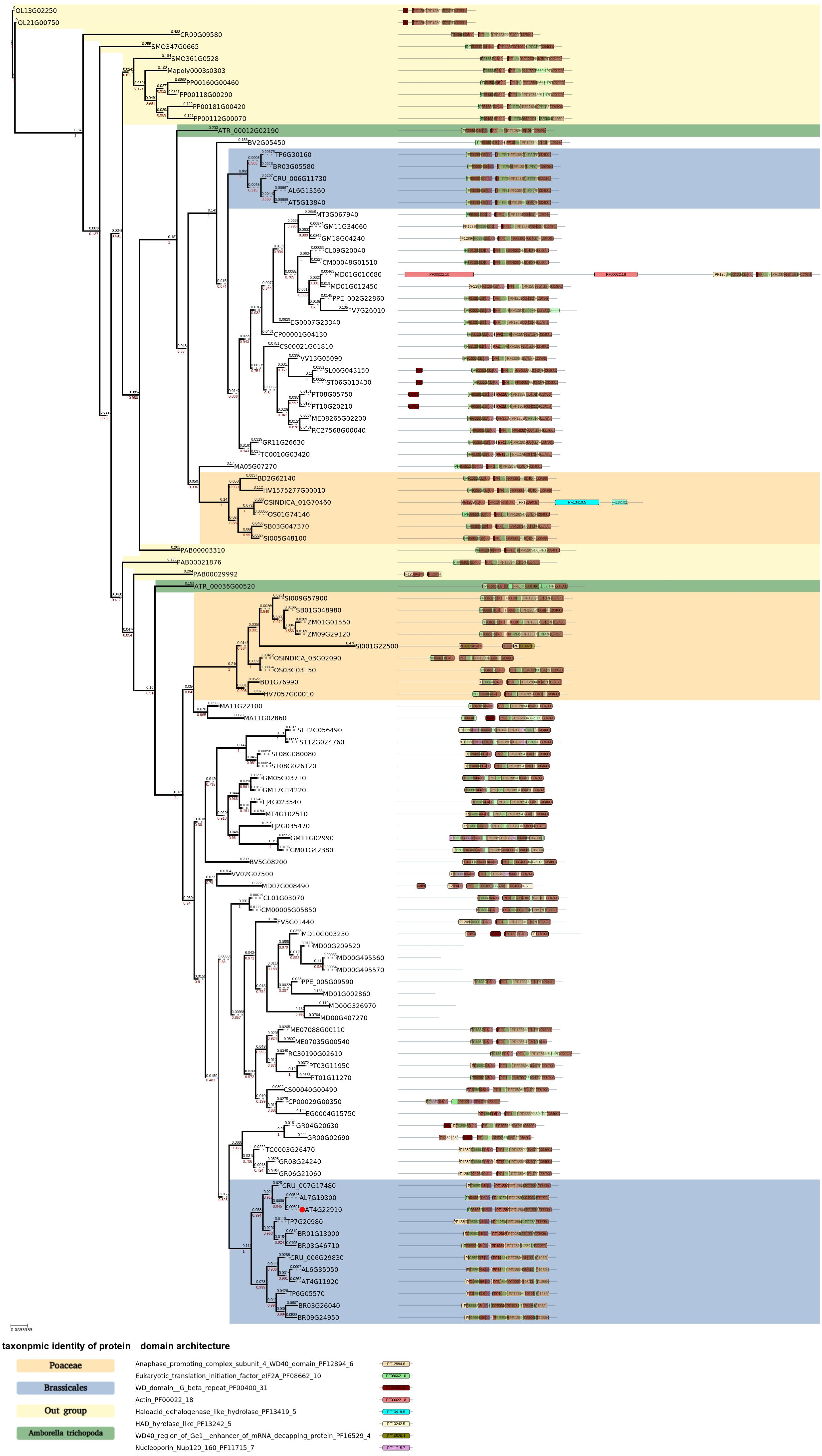

Figure S49. PhyML phylogenetic relations and composition of domains for AT4G24210 homologues of representative plant species. The color legends of species and domains is given below

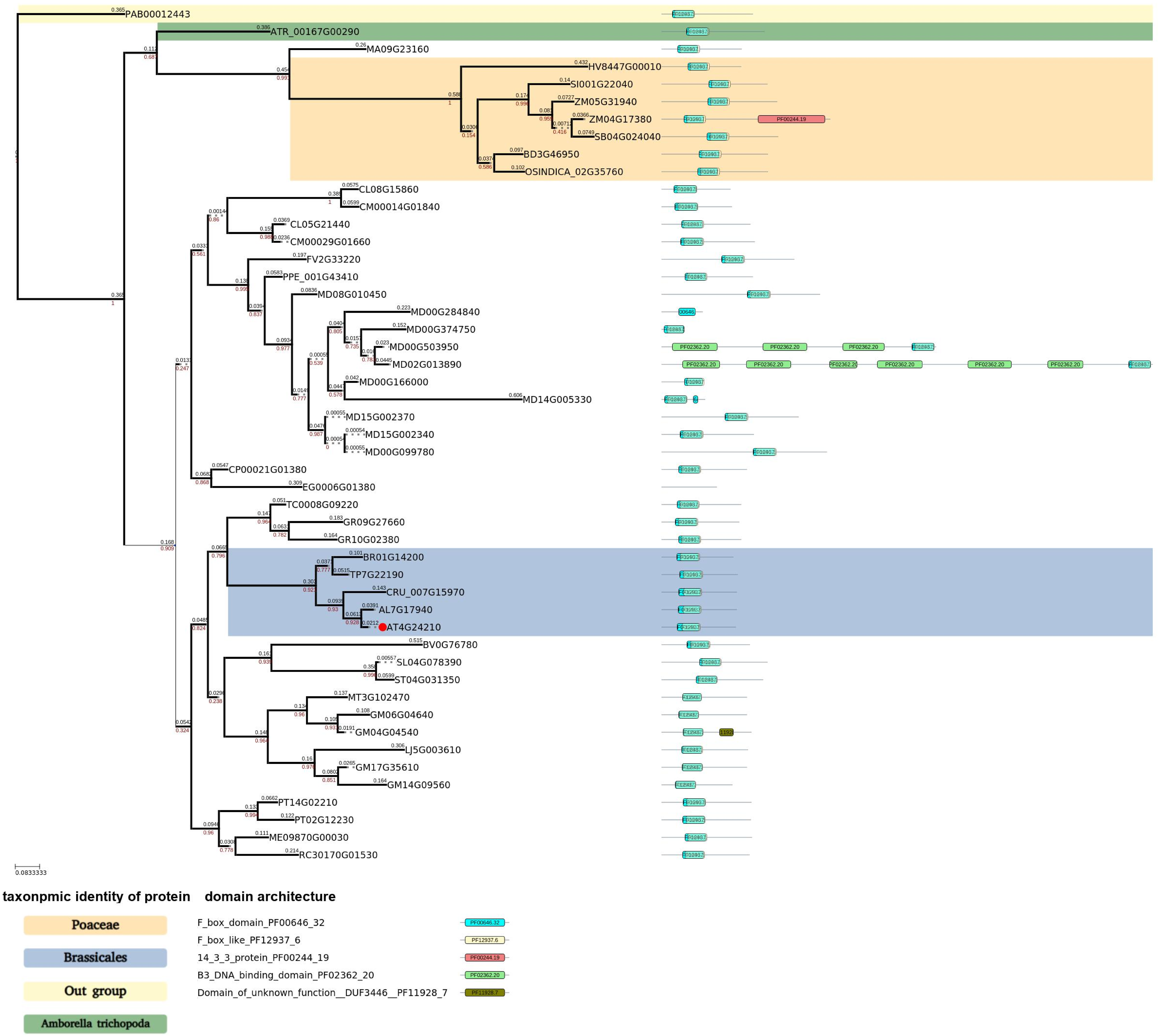

Figure S50. PhyML phylogenetic relations and composition of domains for AT4G38600 homologues of representative plant species. The color legends of species and domains is given below

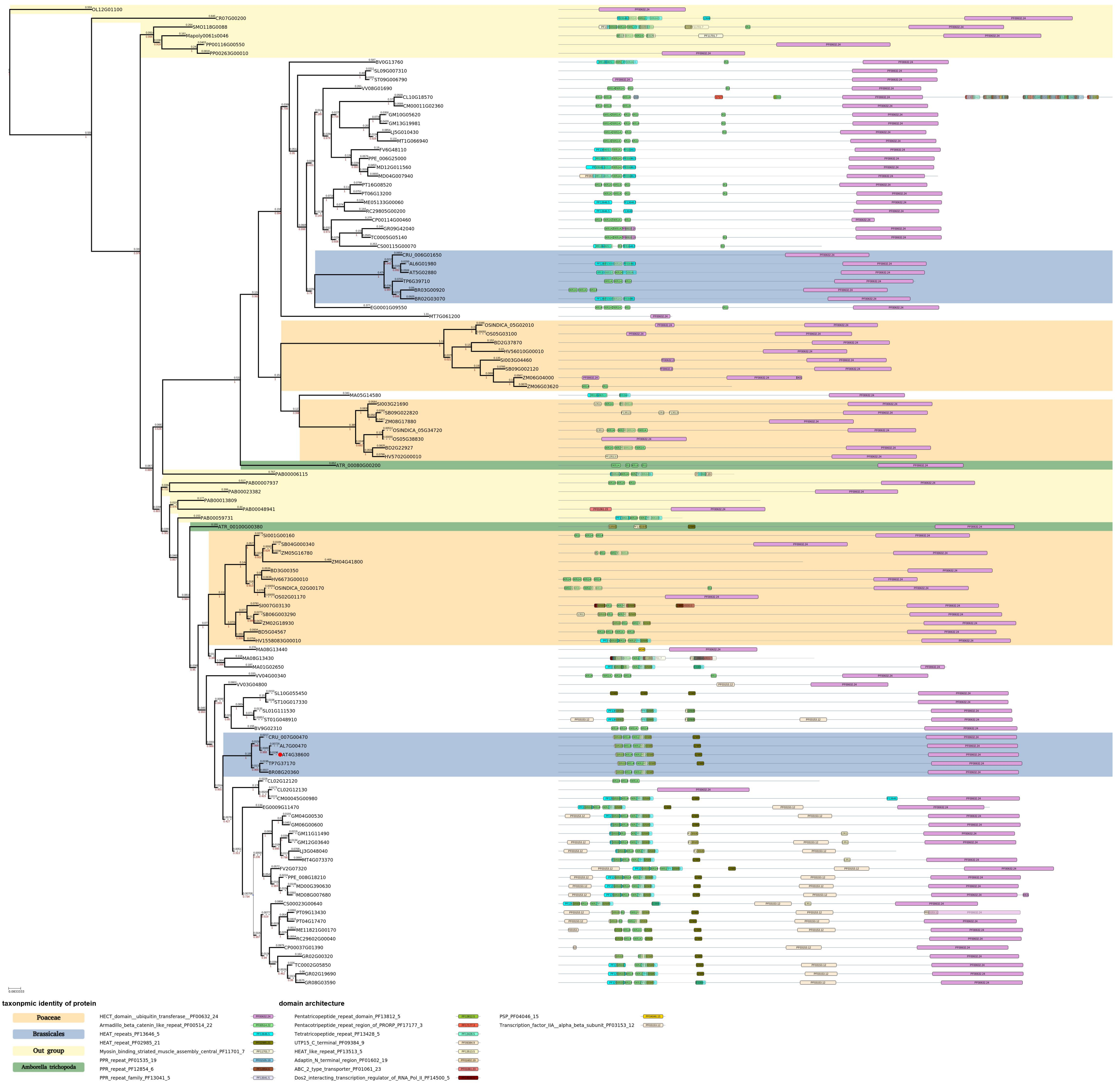

Figure S51. PhyML phylogenetic relations and composition of domains for AT5G04470 homologues of representative plant species. The color legends of species and domains is given below

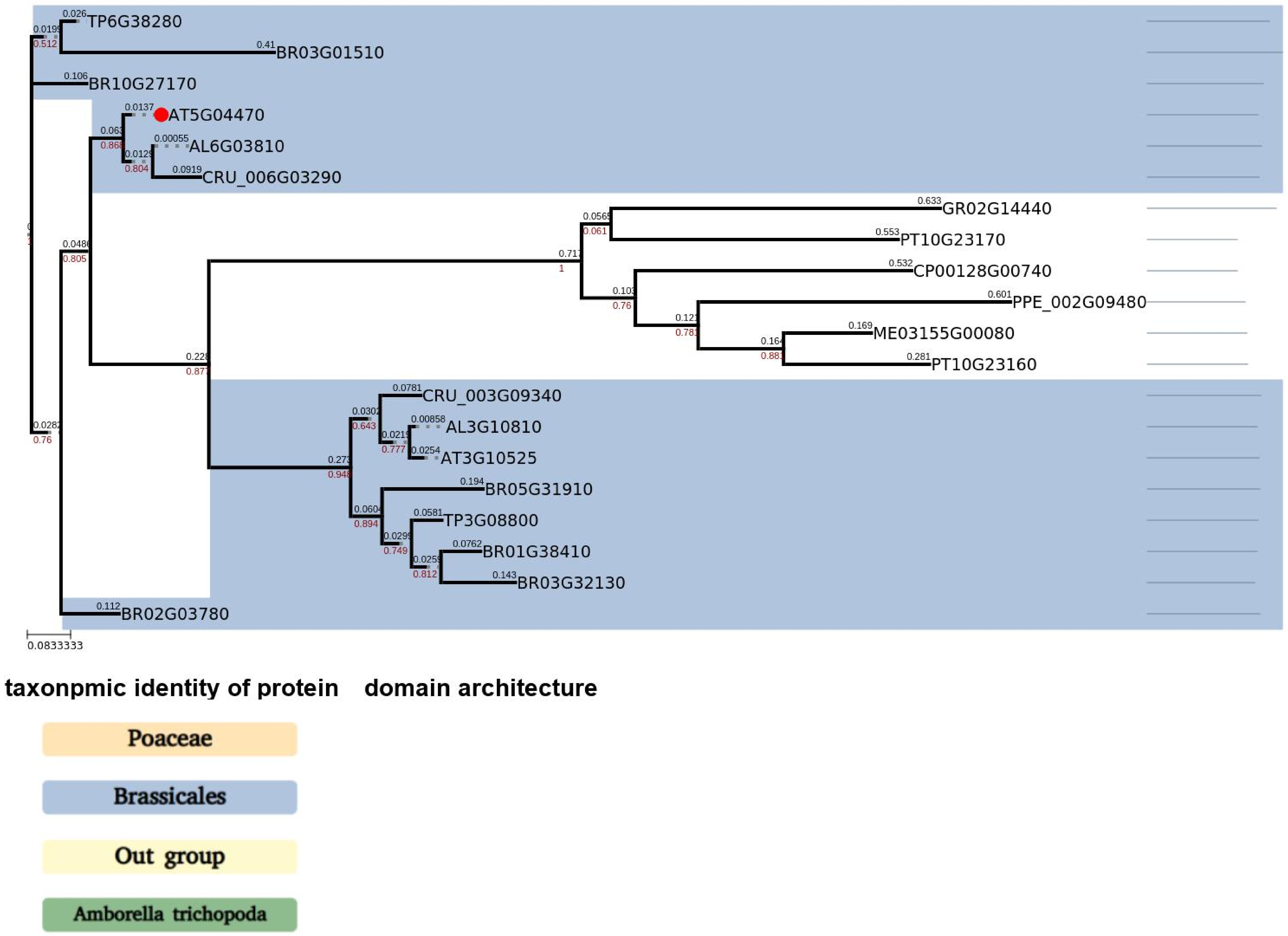

Figure S52. PhyML phylogenetic relations and composition of domains for AT5G06650, AT2G41940, AT3G58070 homologues of representative plant species. The color legends of species and domains is given below

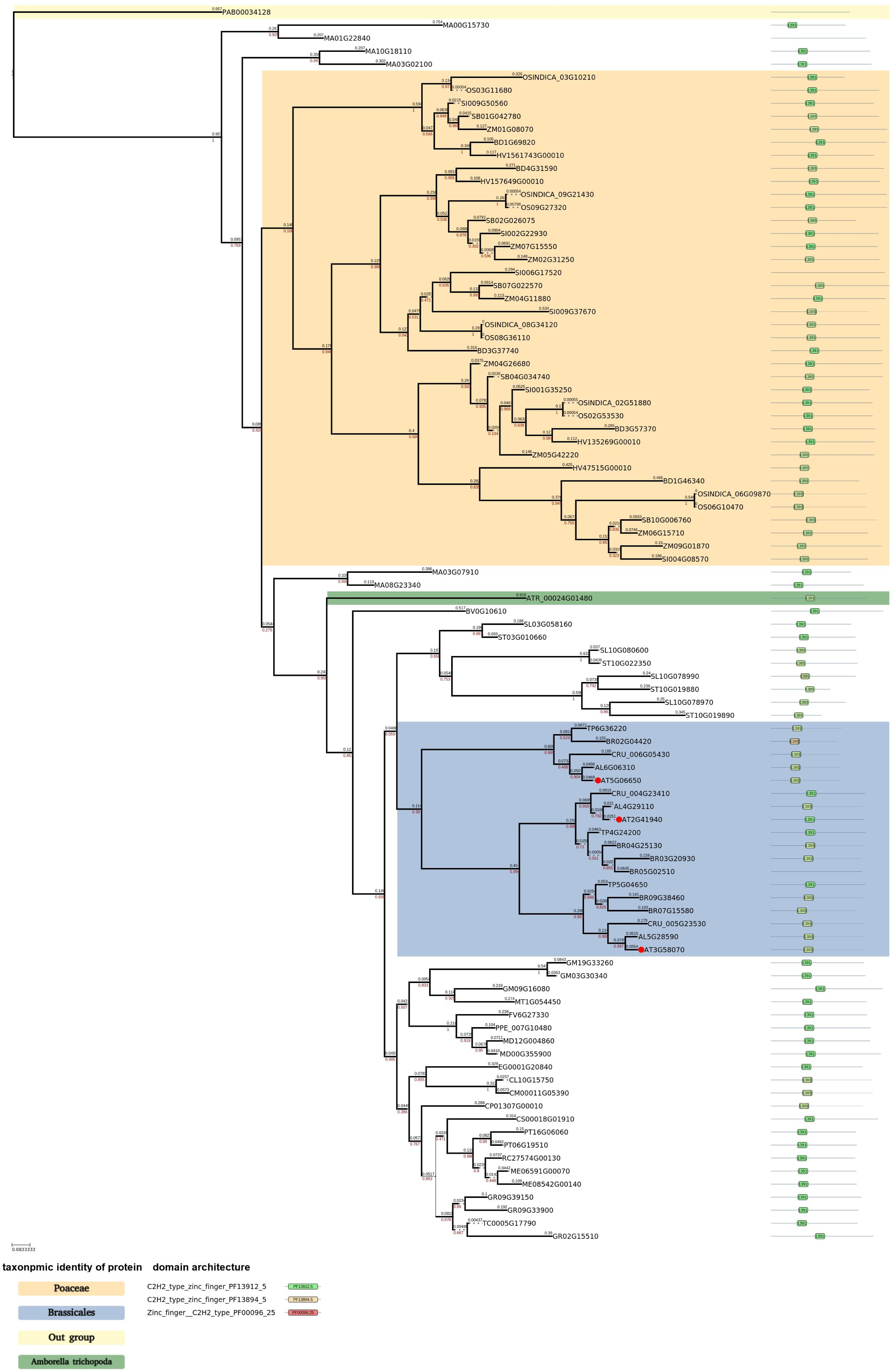

Figure S53. PhyML phylogenetic relations and composition of domains for AT5G18410 homologues of representative plant species. The color legends of species and domains is given below

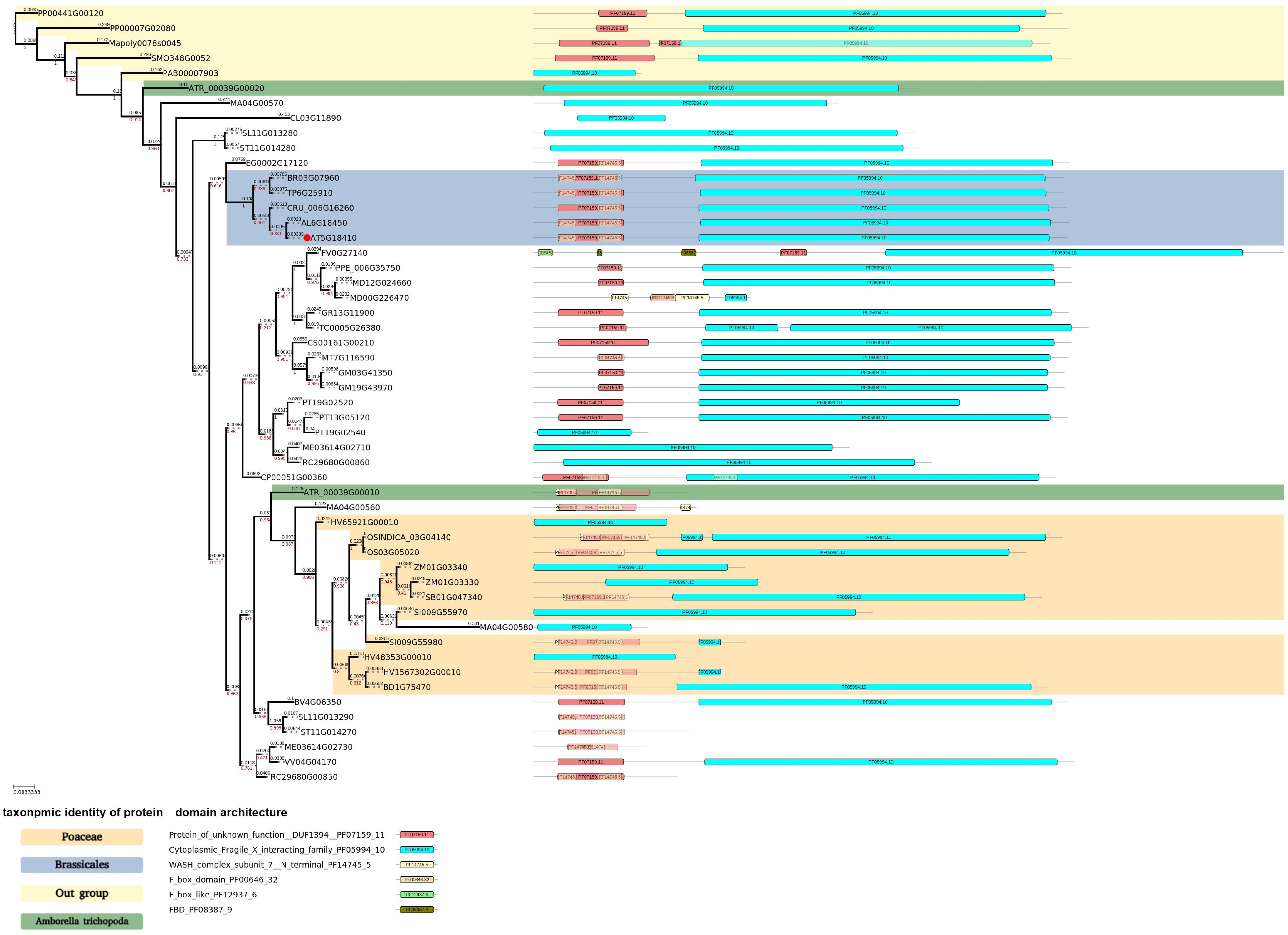

Figure S54. PhyML phylogenetic relations and composition of domains for AT1G17580, AT5G20490 homologues of representative plant species. The color legends of species and domains is given below

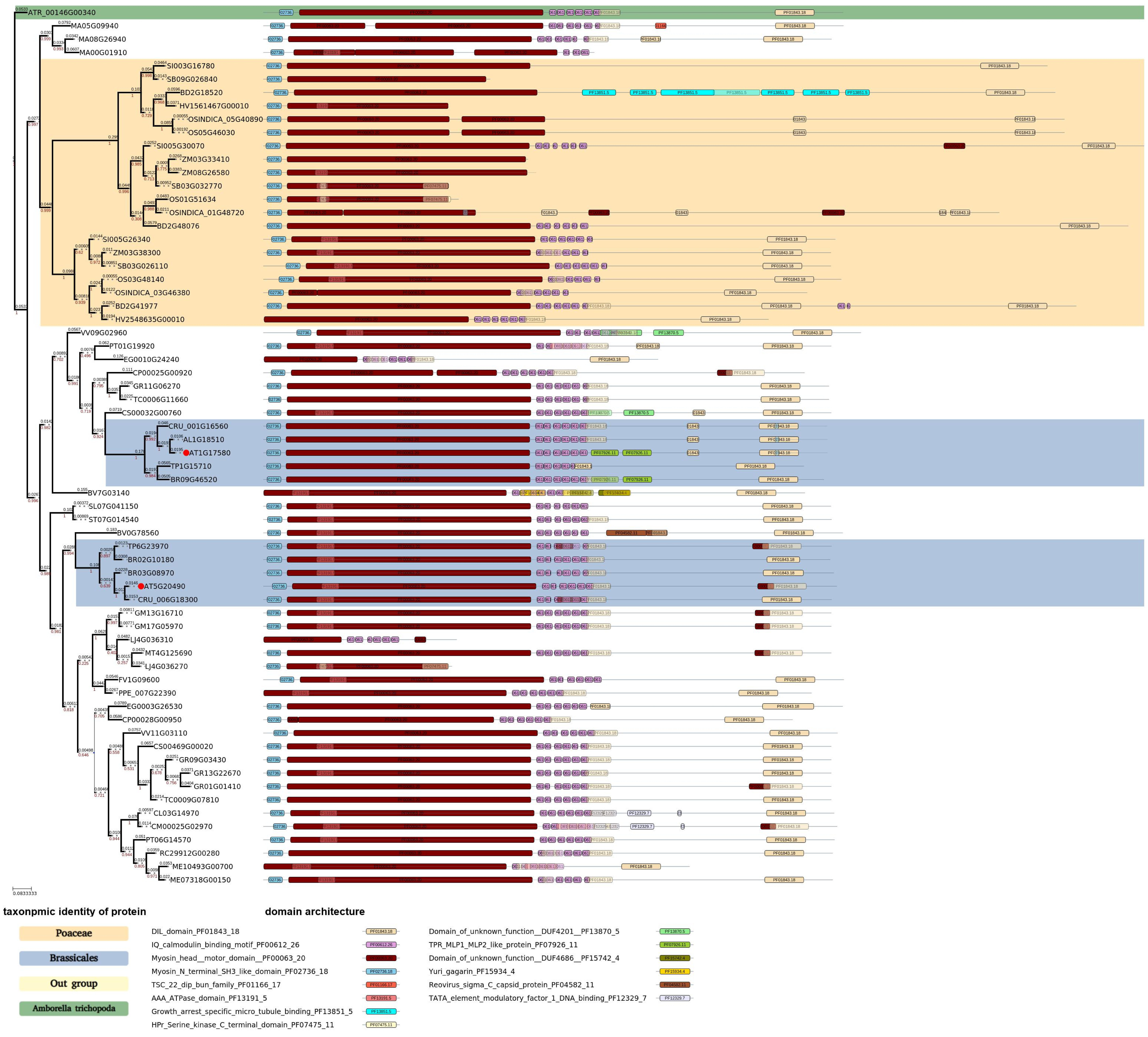

Supplement: Supplementary file 11 — Figure S45: PhyML phylogenetic relations and composition of domains for AT4G14147 homologues of representative plant species. Figure S46: PhyML phylogenetic relations and composition of domains for AT4G15415 homologues of representative plant species. Figure S47: PhyML phylogenetic relations and composition of domains for AT4G20780 homologues of representative plant species. Figure S48: PhyML phylogenetic relations and composition of domains for AT4G22910 homologues of representative plant species. Figure S49: PhyML phylogenetic relations and composition of domains for AT4G24210 homologues of representative plant species. Figure S50: PhyML phylogenetic relations and composition of domains for AT4G38600 homologues of representative plant species. Figure S51: PhyML phylogenetic relations and composition of domains for AT4G04470 homologues of representative plant species. Figure S52: PhyML phylogenetic relations and composition of domains for AT5G06650; AT2G41940; AT3G58070 homologues of representative plant species. Figure S53: PhyML phylogenetic relations and composition of domains for AT5G18410 homologues of representative plant species. Figure S54: PhyML phylogenetic relations and composition of domains for AT1G17580; AT5G20490 homologues of representative plant species. (PDF 7225 kb) [file 12870_2019_1640_MOESM11_ESM.pdf]
