# Supplementary material for: The evolution of gene regulatory networks controlling Arabidopsis thaliana L. trichome development
Source: BMC Plant Biol. 2019 Feb 15;19(Suppl 1):53. doi: 10.1186/s12870-019-1640-2 (PMC6393967; doi:10.1186/s12870-019-1640-2)

Figure S55. PhyML phylogenetic relations and composition of domains for AT5G20570 homologues of representative plant species. The color legends of species and domains is given below

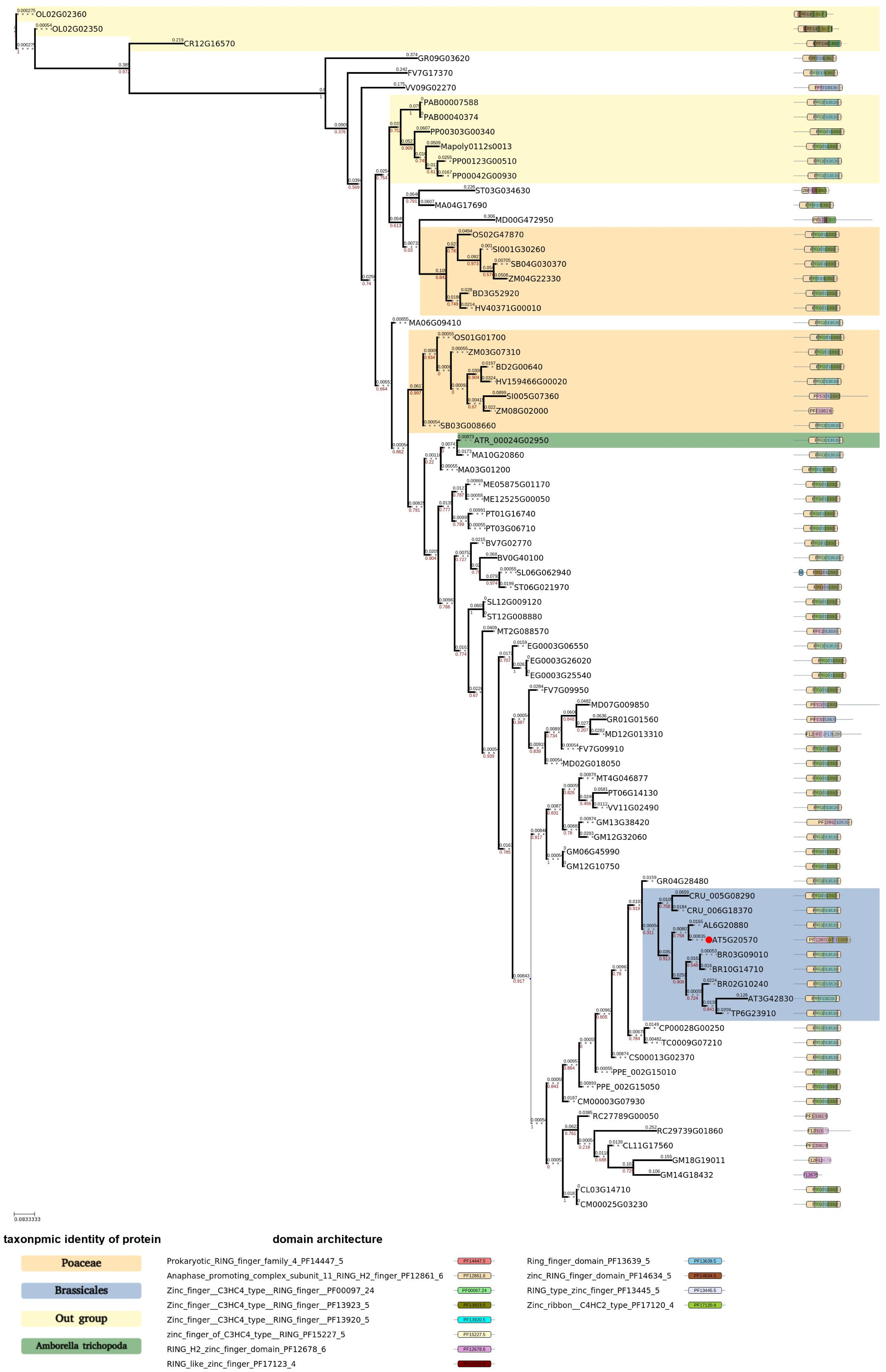

Figure S56. PhyML phylogenetic relations and composition of domains for AT2G36010, AT1G47870, AT5G22220 homologues of representative plant species. The color legends of species and domains is given below

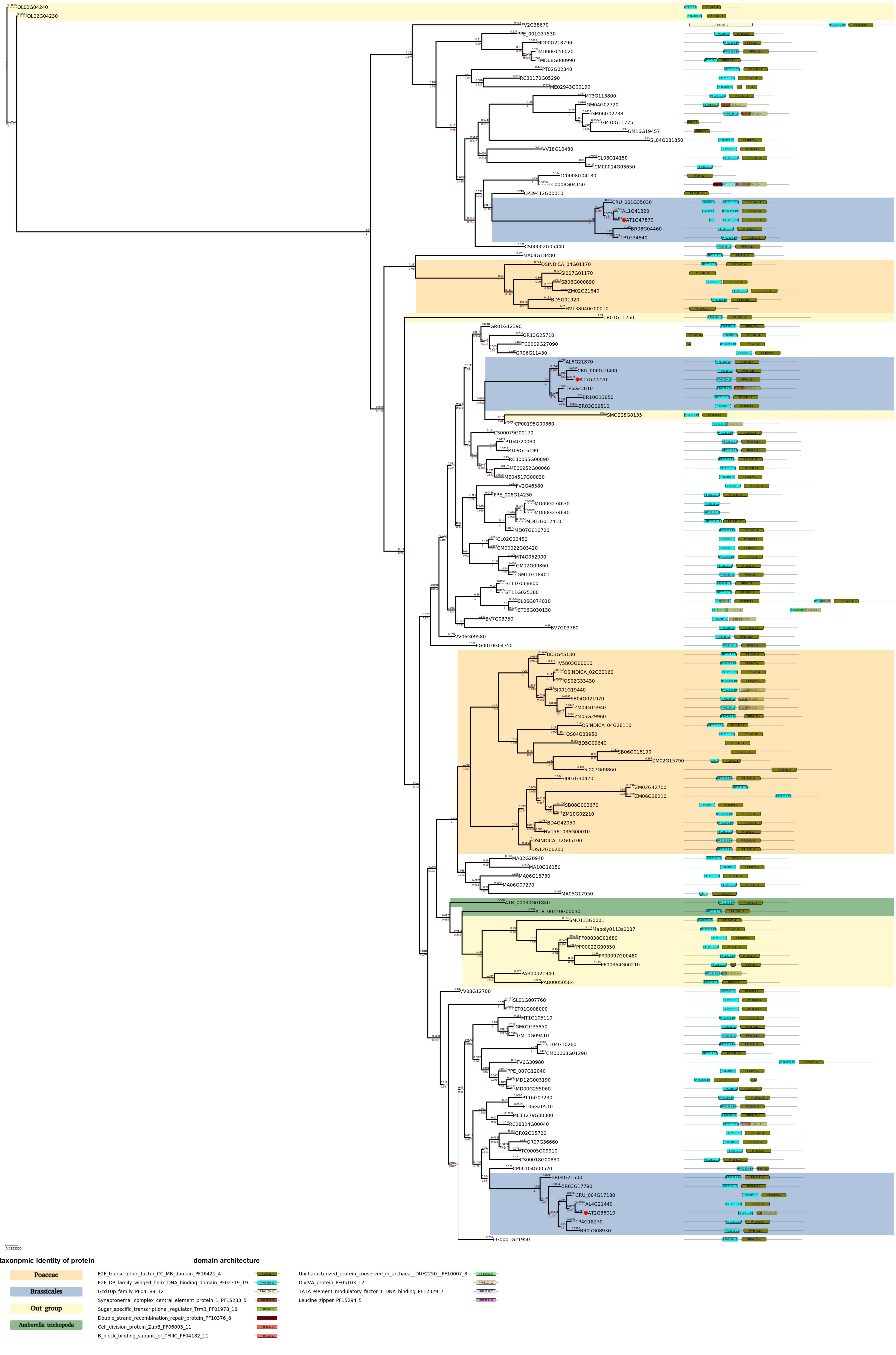

Figure S57. PhyML phylogenetic relations and composition of domains for AT5G24310, AT5G42030, AT2G46225 homologues of representative plant species. The color legends of species and domains is given below

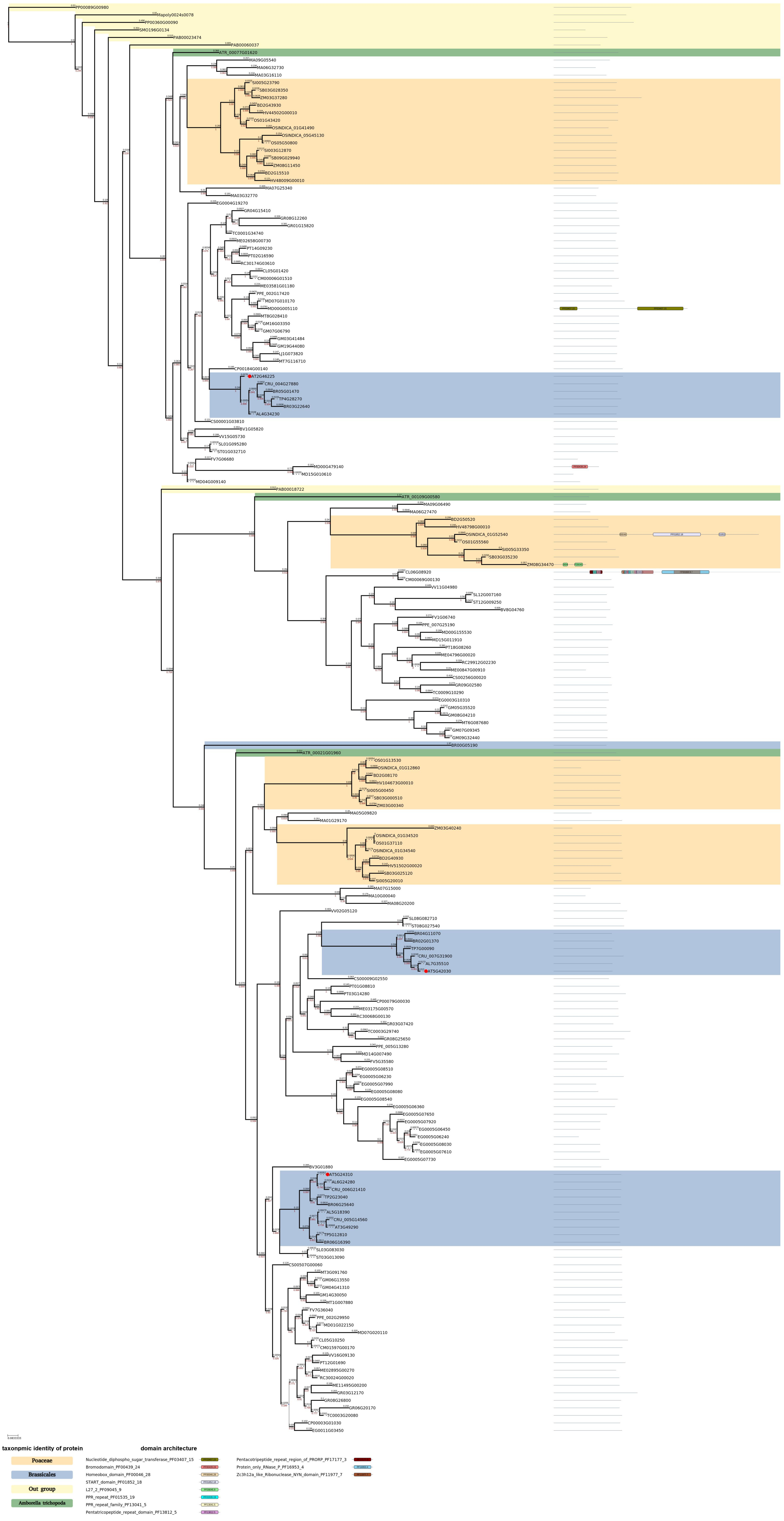

Figure S58. PhyML phylogenetic relations and composition of domains for AT5G28646 homologues of representative plant species. The color legends of species and domains is given below

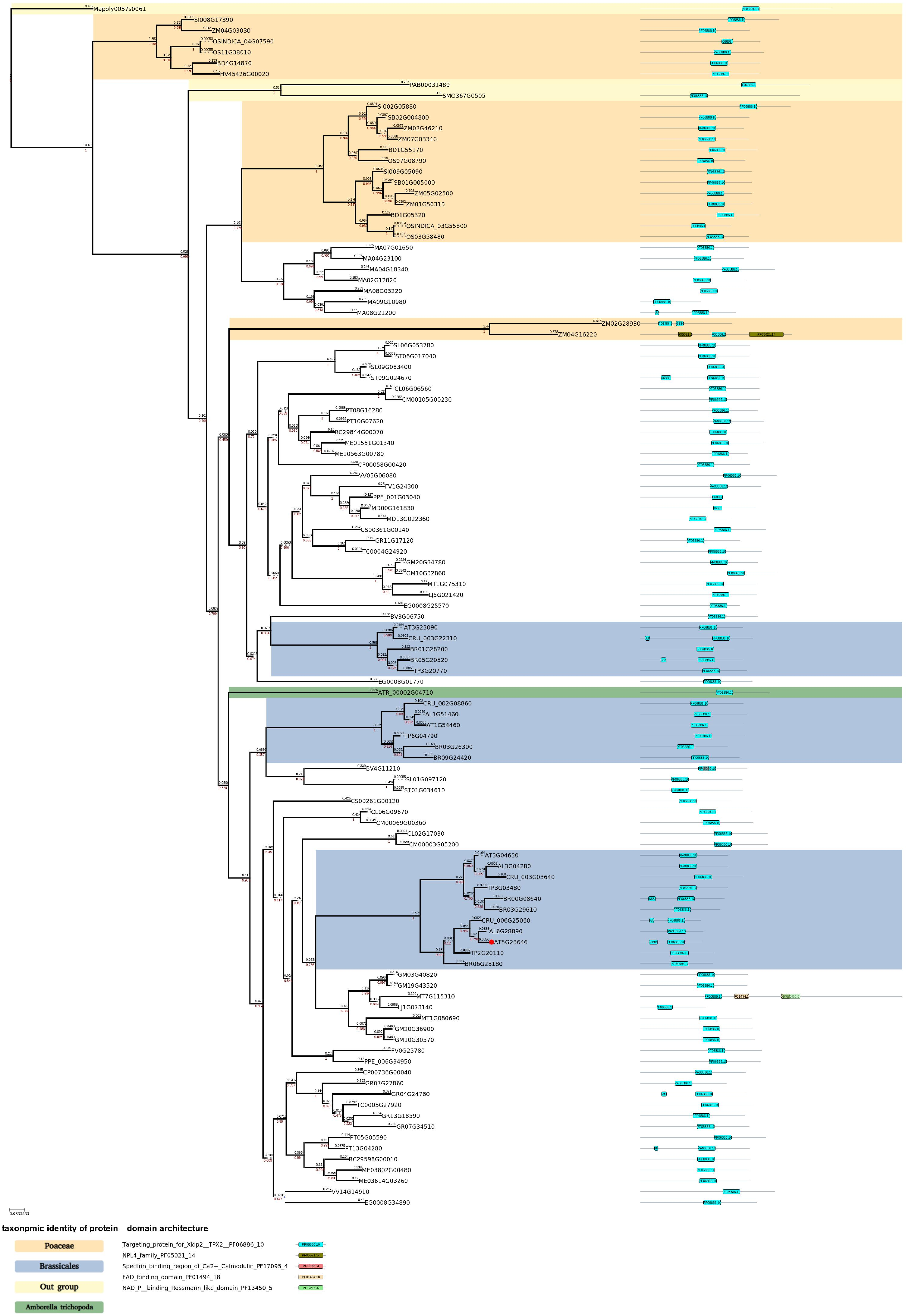

Figure S59. PhyML phylogenetic relations and composition of domains for AT5G42080 homologues of representative plant species. The color legends of species and domains is given below

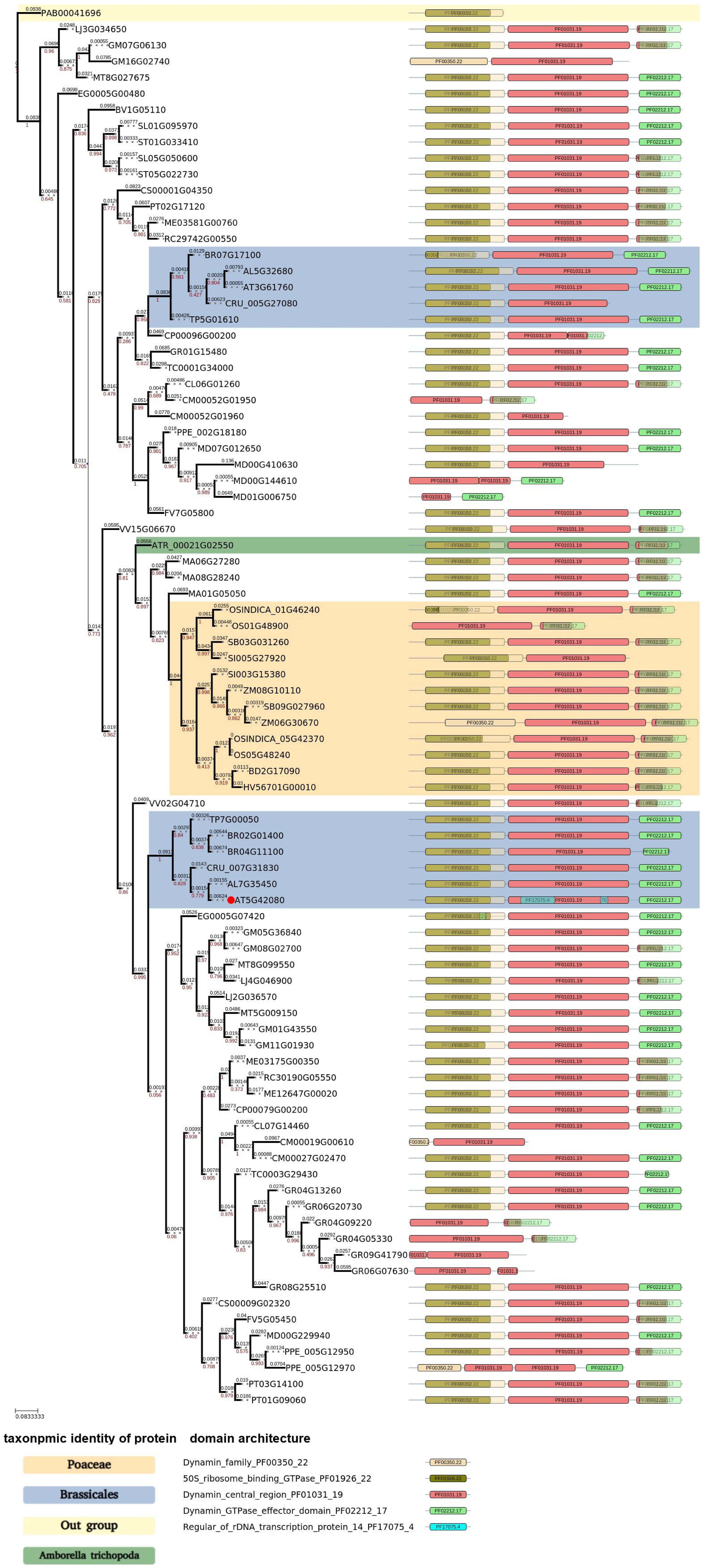

Figure S60. PhyML phylogenetic relations and composition of domains for AT5G43900 homologues of representative plant species. The color legends of species and domains is given below

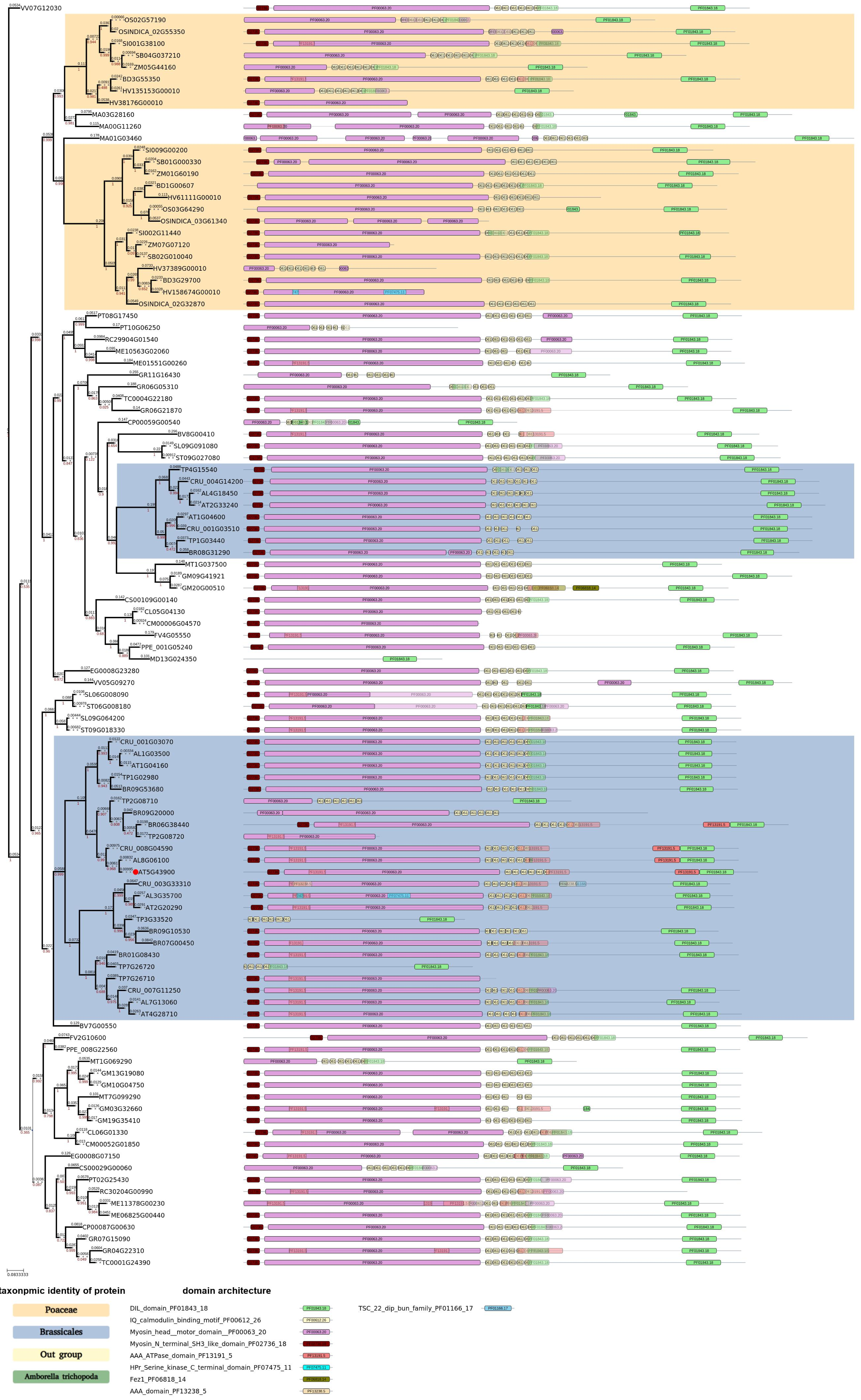

Figure S61. PhyML phylogenetic relations and composition of domains for AT5G45190, AT4G19600 homologues of representative plant species. The color legends of species and domains is given below

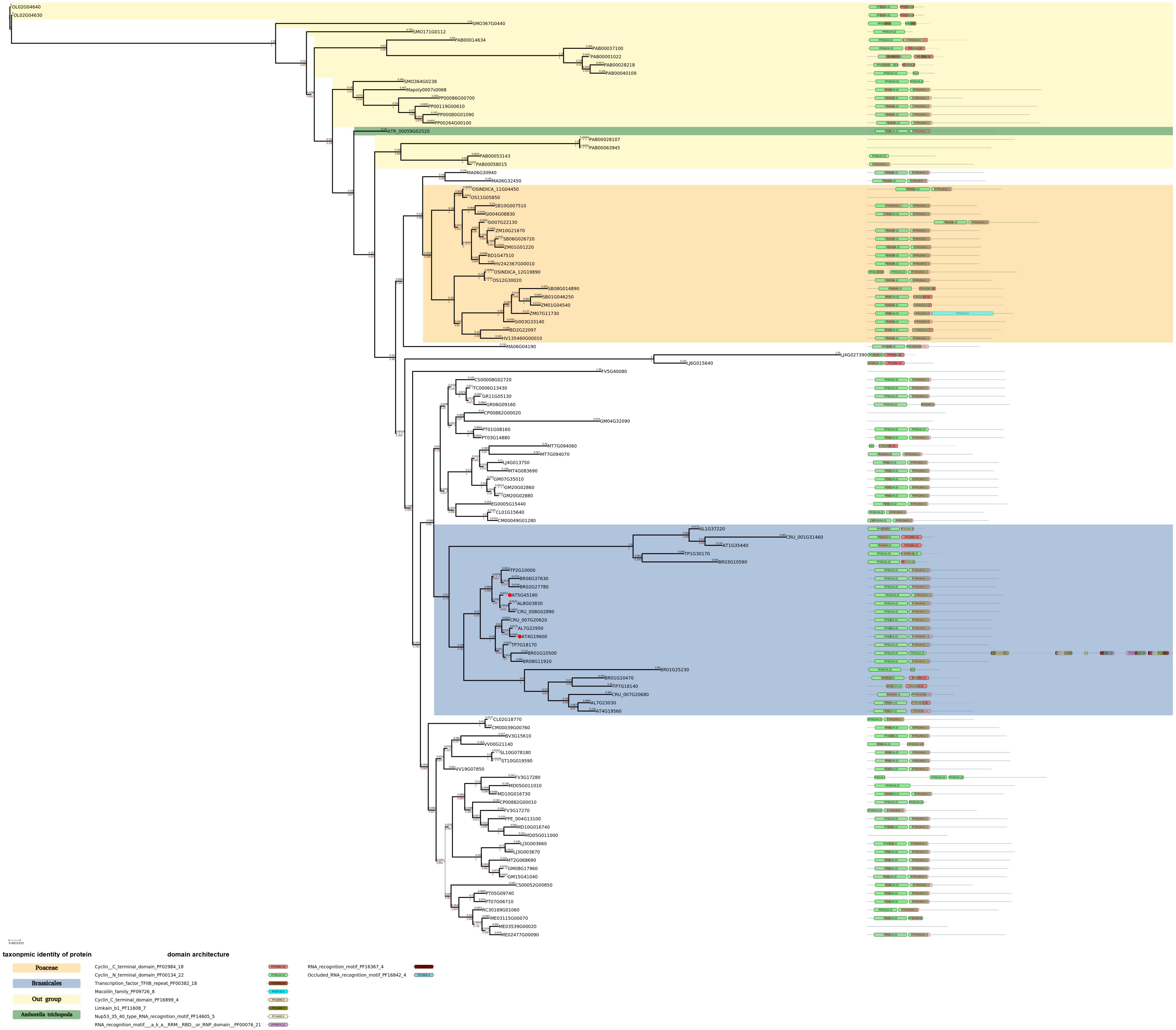

Figure S62. PhyML phylogenetic relations and composition of domains for AT2G33540 homologues of representative plant species. The color legends of species and domains is given below

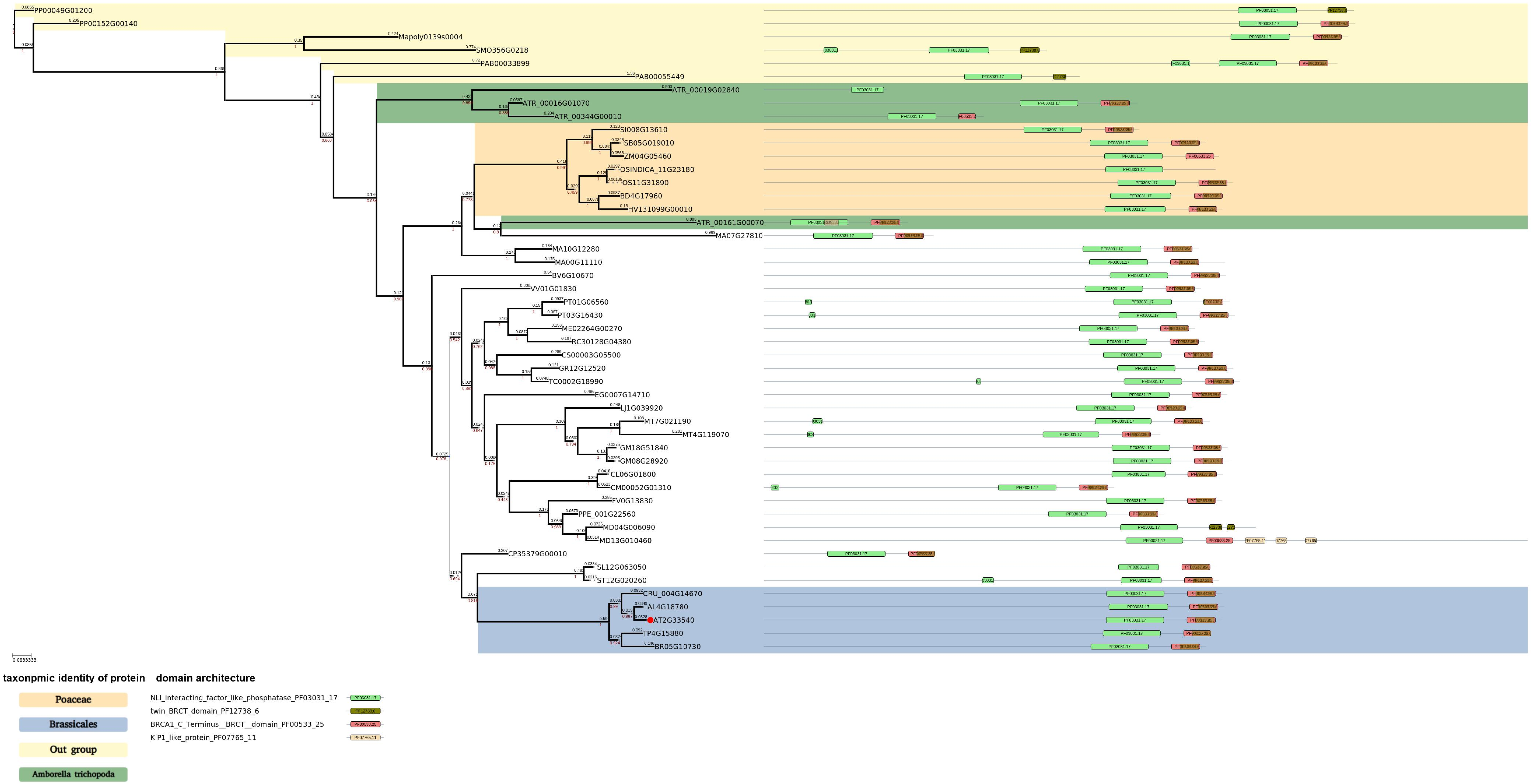

Figure S63. PhyML phylogenetic relations and composition of domains for AT5G58230 homologues of representative plant species. The color legends of species and domains is given below

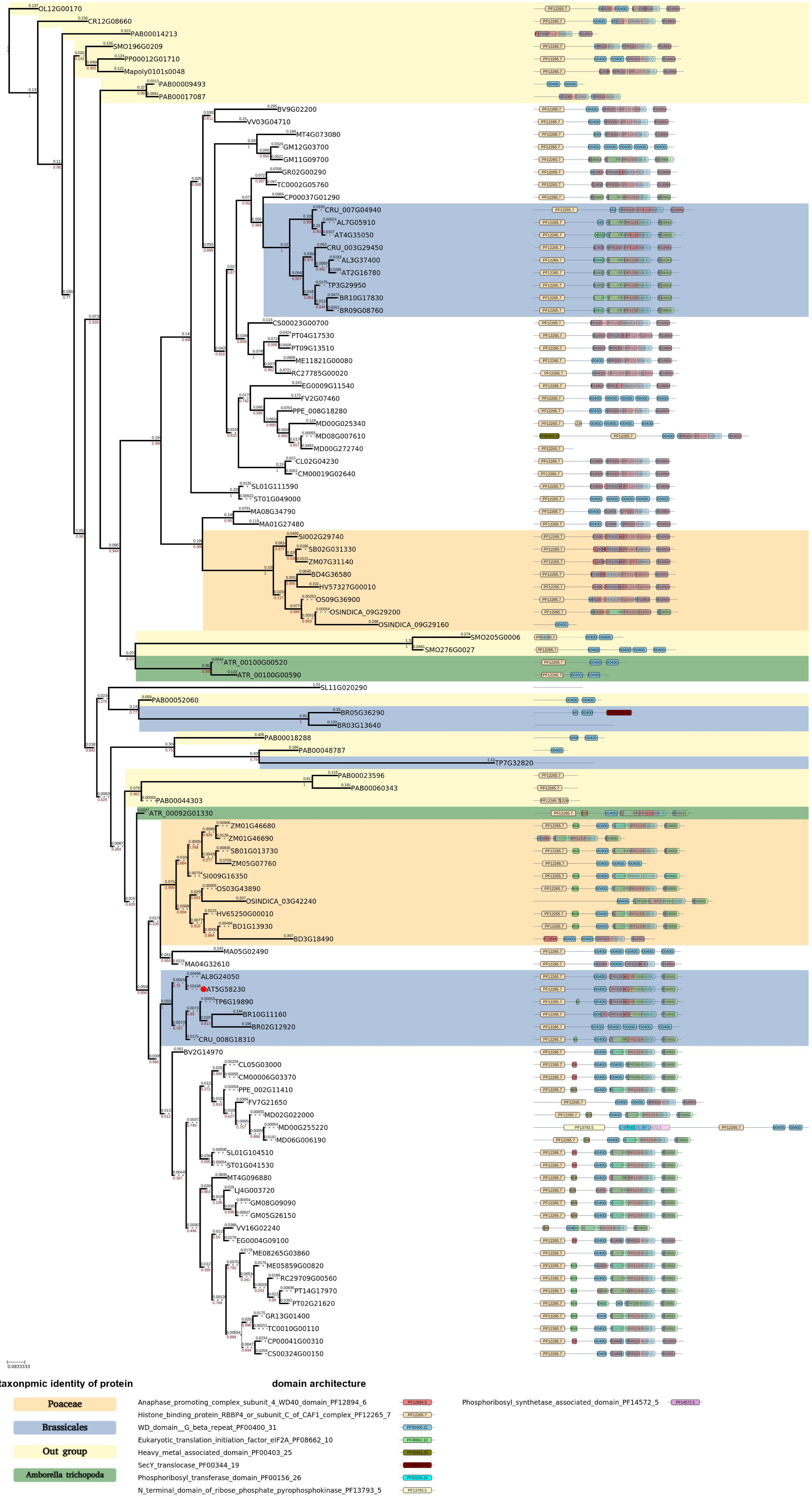

Figure S64. PhyML phylogenetic relations and composition of domains for AT5G64630 homologues of representative plant species. The color legends of species and domains is given below

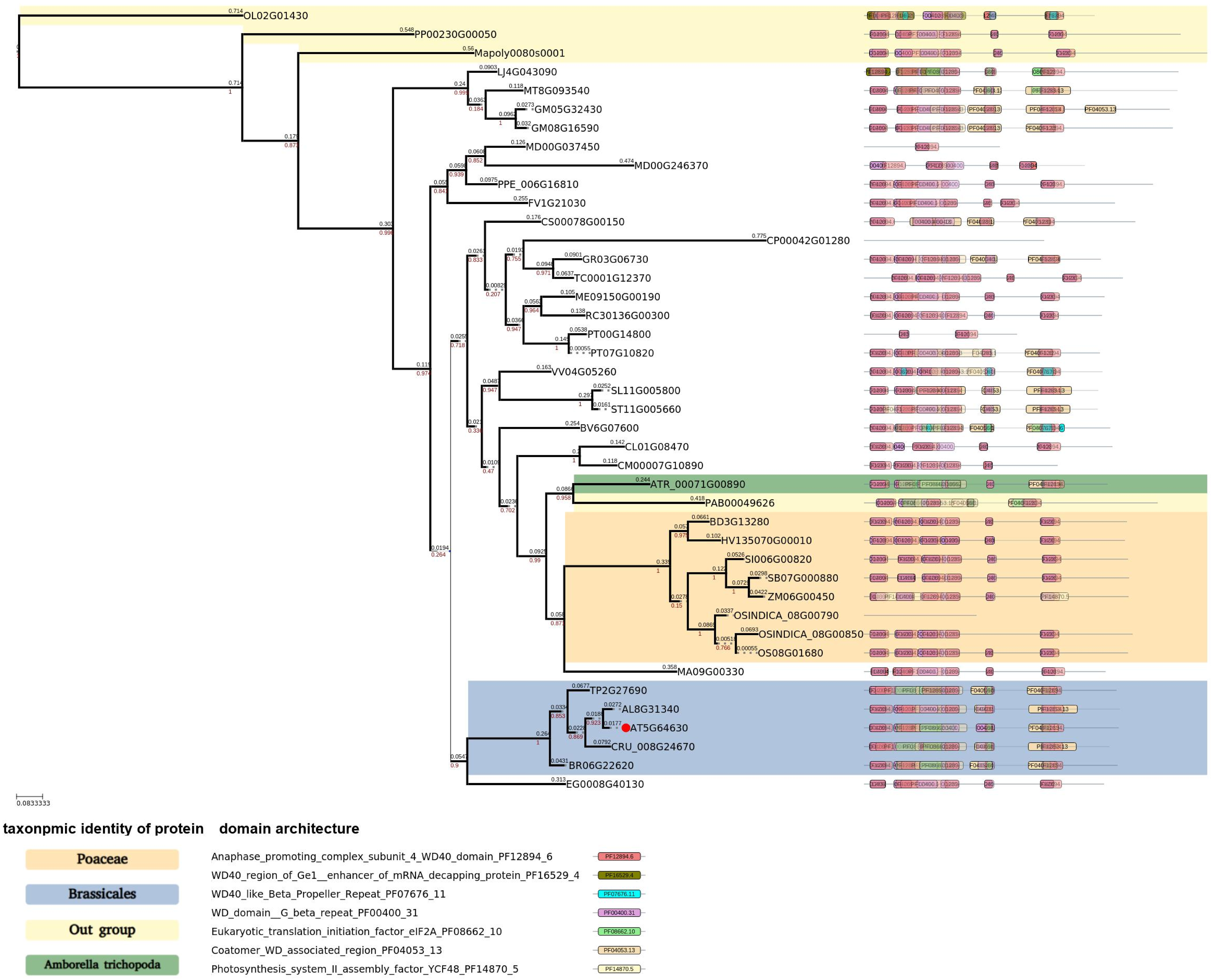

Figure S65. PhyML phylogenetic relations and composition of domains for AT5G65930 homologues of representative plant species. The color legends of species and domains is given below

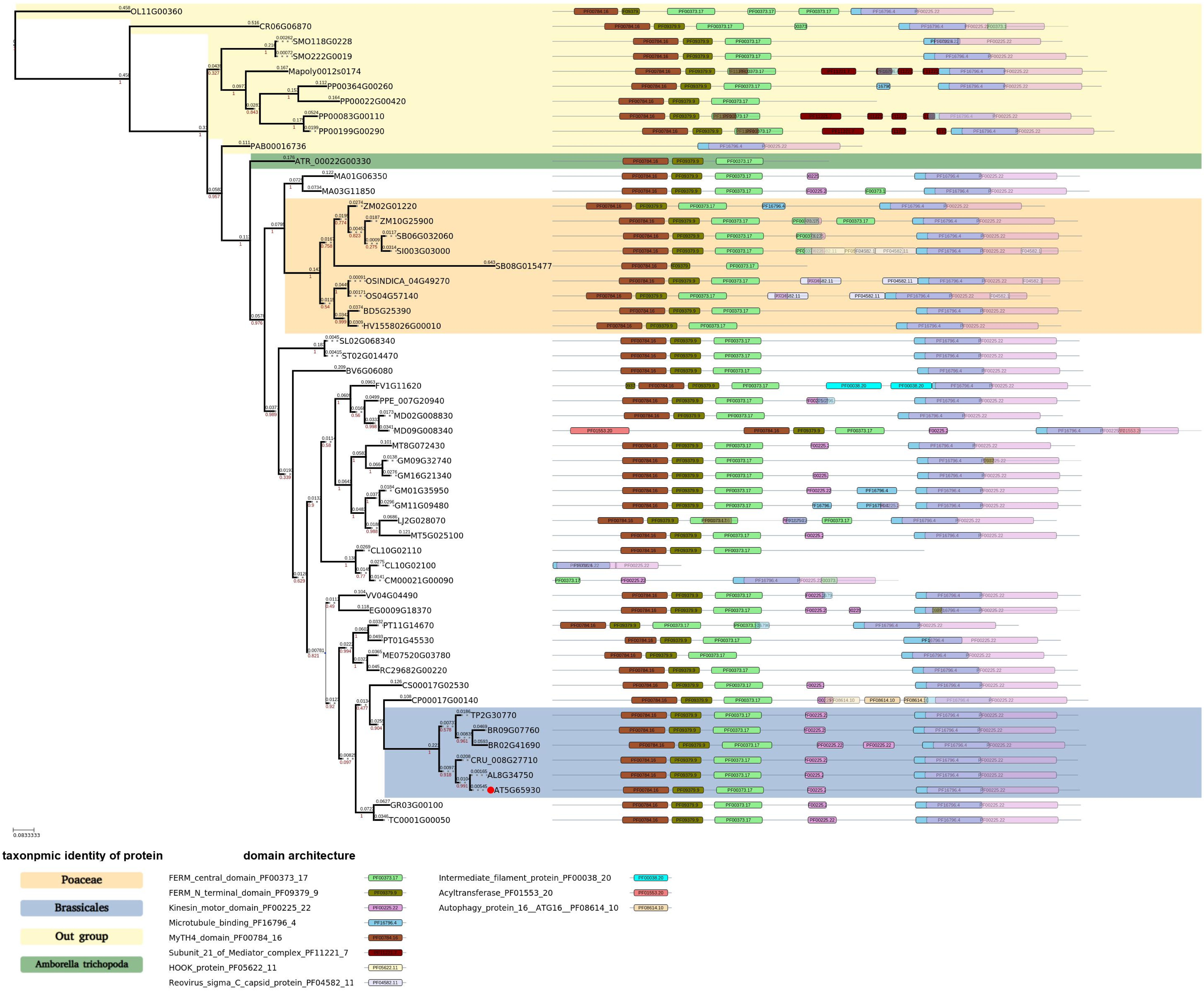

Supplement: Supplementary file 12 — Figure S55: PhyML phylogenetic relations and composition of domains for AT5G20570 homologues of representative plant species. Figure S56: PhyML phylogenetic relations and composition of domains for AT2G36010; AT1G47870; AT5G22220 homologues of representative plant species. Figure S57: PhyML phylogenetic relations and composition of domains for AT5G24310; AT5G42030; AT2G46225 homologues of representative plant species. Figure S58: PhyML phylogenetic relations and composition of domains for AT5G28646 homologues of representative plant species. Figure S59: PhyML phylogenetic relations and composition of domains for AT5G42080 homologues of representative plant species. Figure S60: PhyML phylogenetic relations and composition of domains for AT5G43900 homologues of representative plant species. Figure S61: PhyML phylogenetic relations and composition of domains for AT5G45190; AT4G19600 homologues of representative plant species. Figure S62: PhyML phylogenetic relations and composition of domains for AT2G33540 homologues of representative plant species. Figure S63: PhyML phylogenetic relations and composition of domains for AT5G58230 homologues of representative plant species. Figure S64: PhyML phylogenetic relations and composition of domains for AT5G64630 homologues of representative plant species. Figure S65: PhyML phylogenetic relations and composition of domains for AT5G65930 homologues of representative plant species. (PDF 9760 kb) [file 12870_2019_1640_MOESM12_ESM.pdf]
