# Supplementary material for: The evolution of gene regulatory networks controlling Arabidopsis thaliana L. trichome development
Source: BMC Plant Biol. 2019 Feb 15;19(Suppl 1):53. doi: 10.1186/s12870-019-1640-2 (PMC6393967; doi:10.1186/s12870-019-1640-2)

Figure S5. PhyML phylogenetic relations and composition of domains for AT1G01510 homologues of representative plant species. The color legends of species and domains is given below

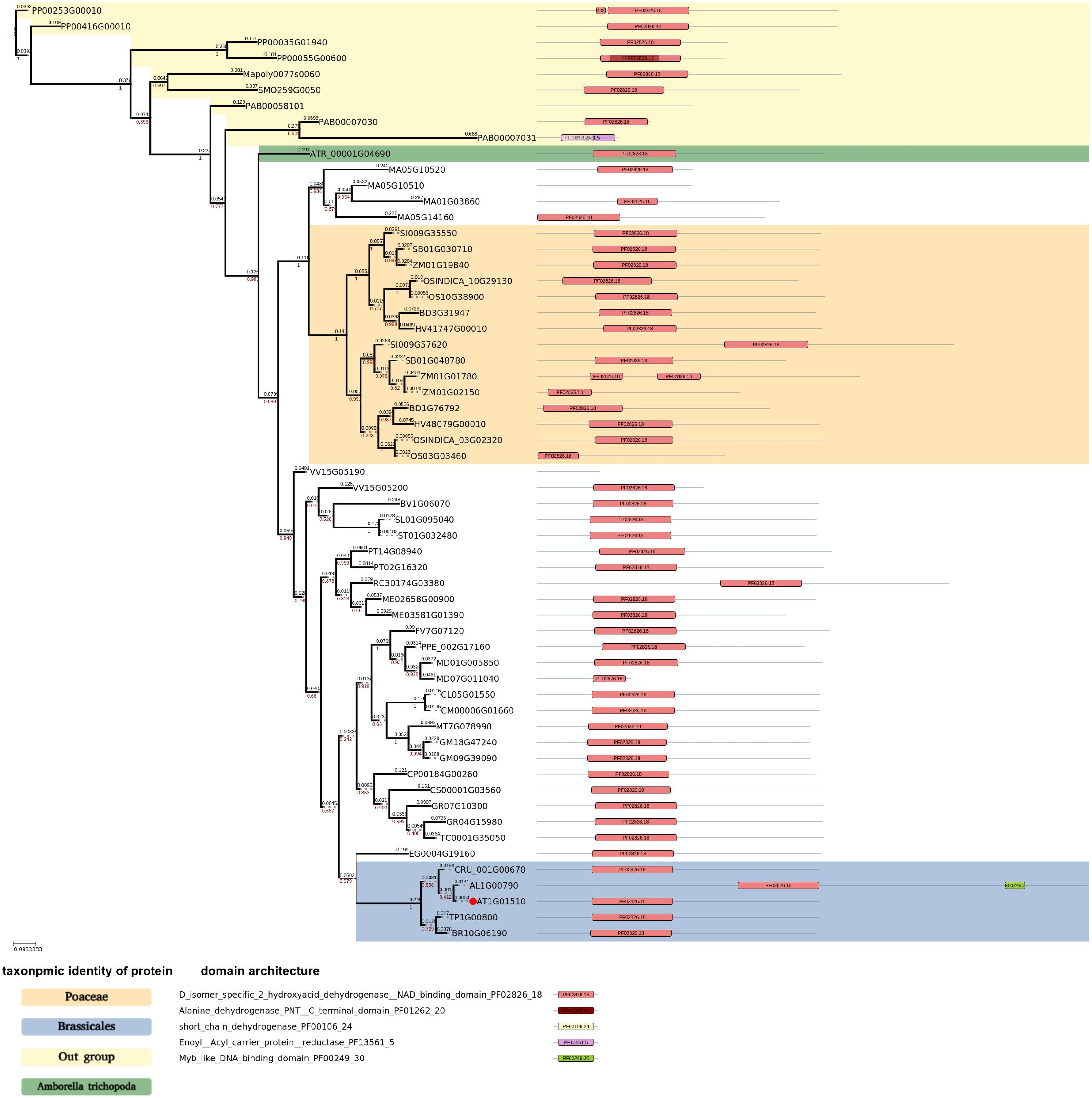

Figure S6. PhyML phylogenetic relations and composition of domains for AT1G01520 homologues of representative plant species. The color legends of species and domains is given below

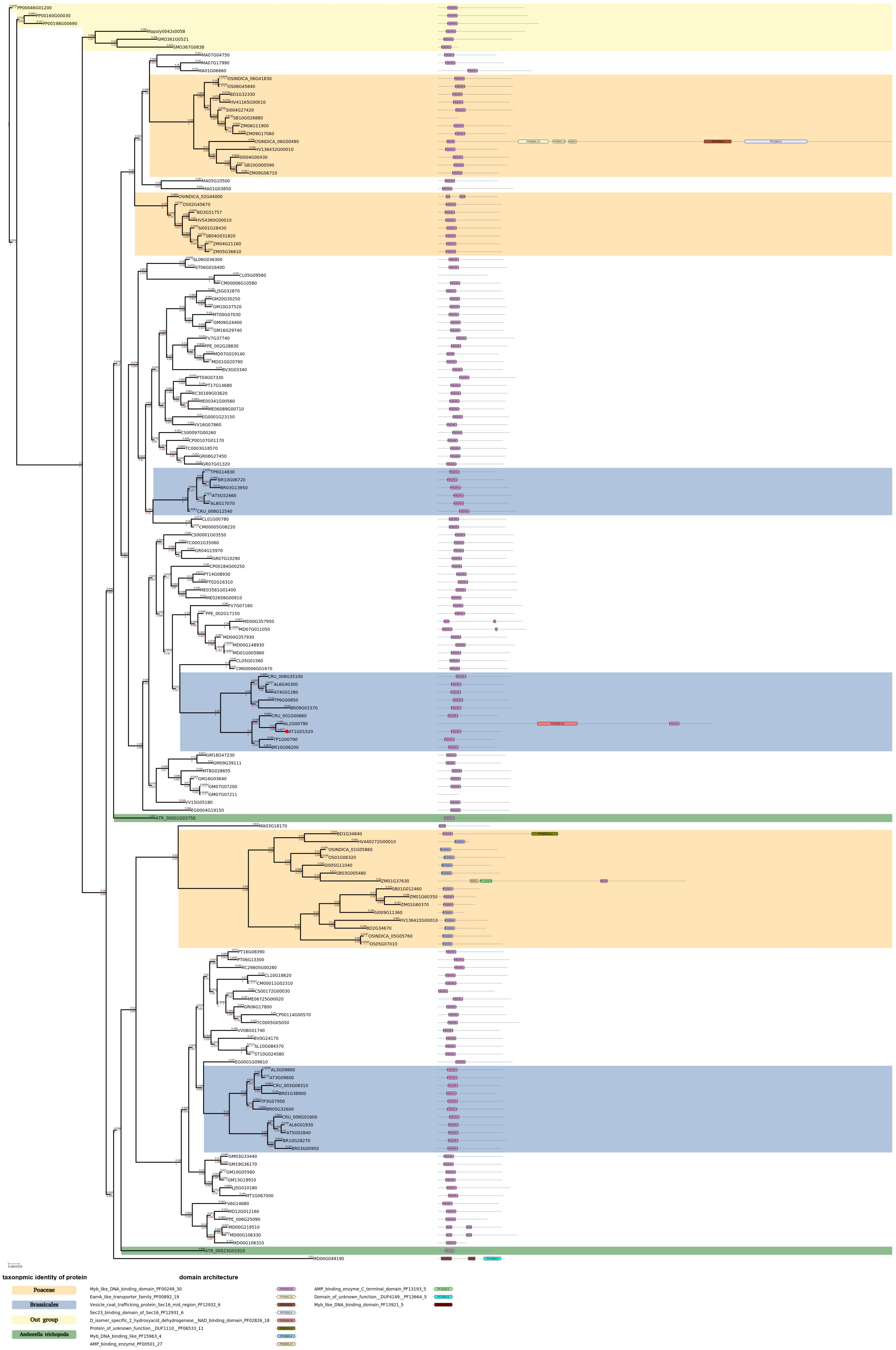

Figure S7. PhyML phylogenetic relations and composition of domains for AT1G03060 homologues of representative plant species. The color legends of species and domains is given below

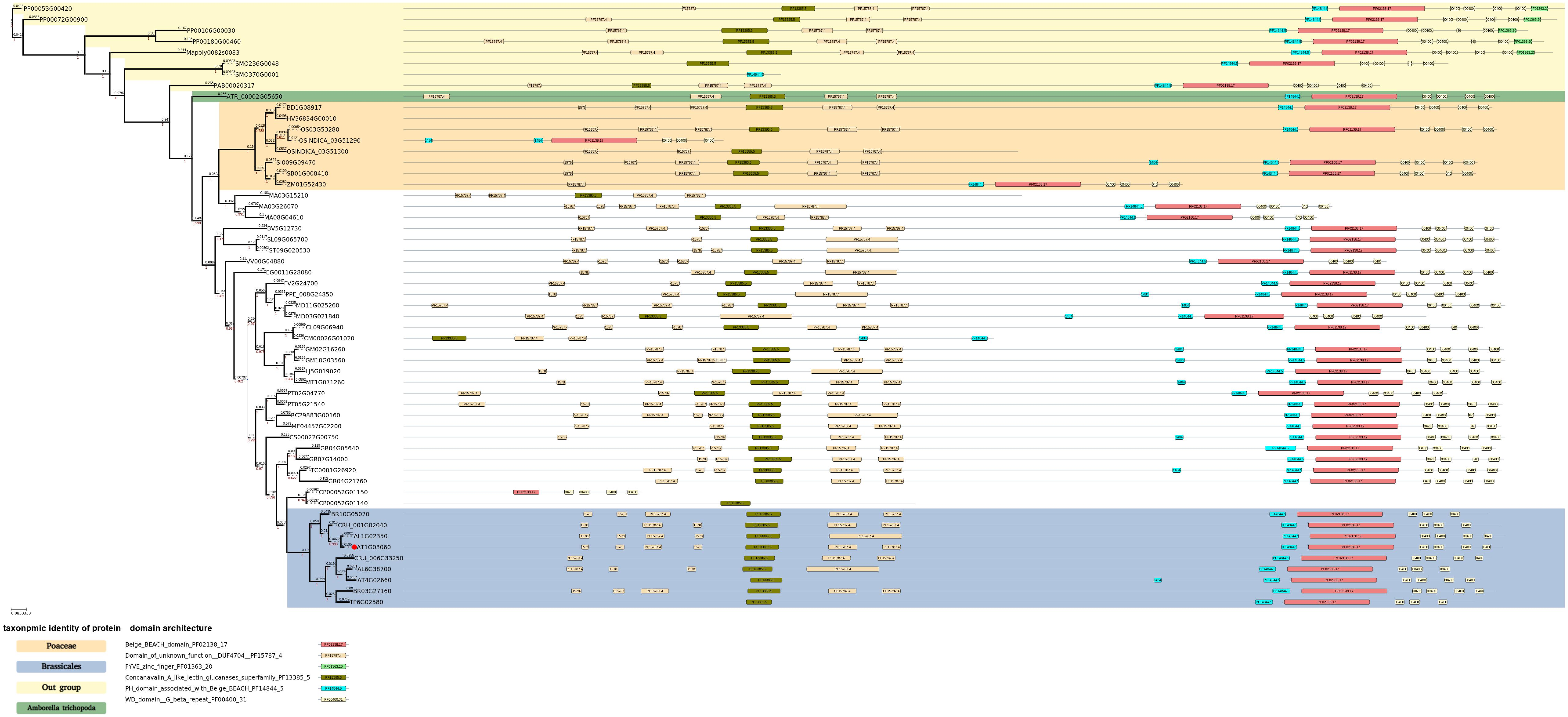

Figure S8. PhyML phylogenetic relations and composition of domains for AT1G05230, AT4G21750, AT4G04890, AT1G79840 homologues of representative plant species. The color legends of species and domains is given below

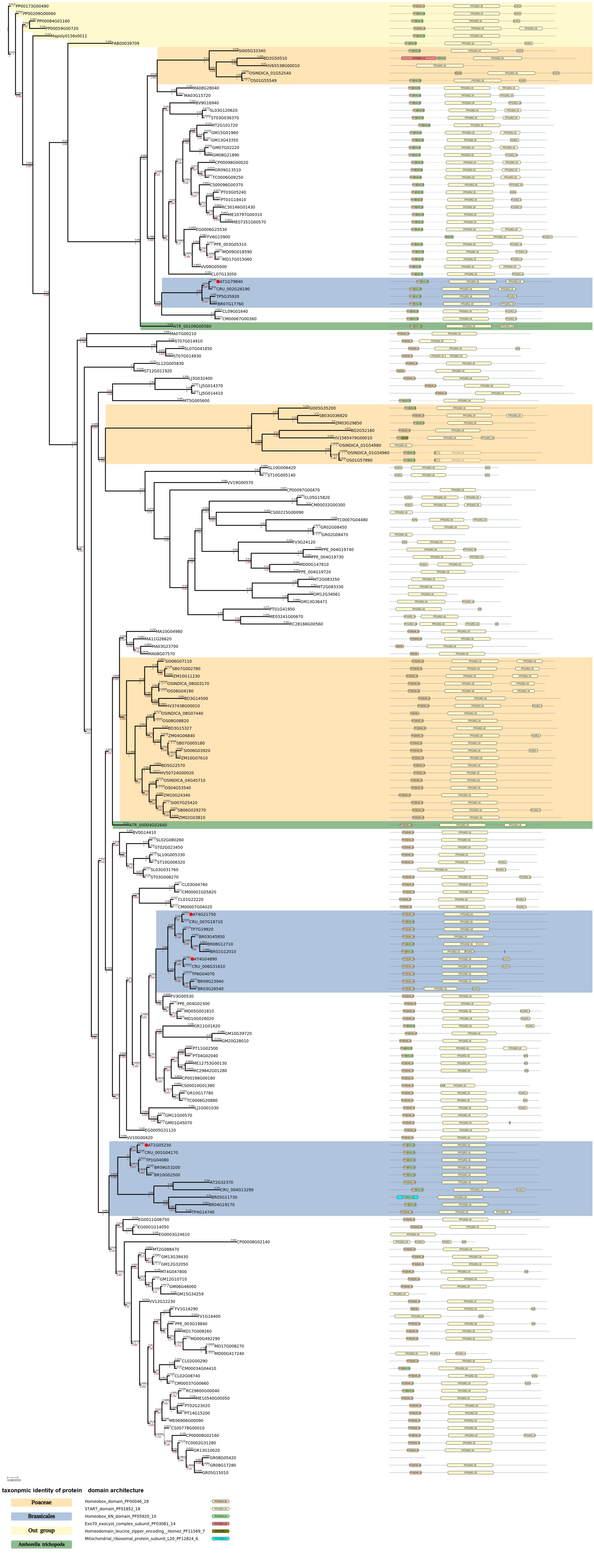

Figure S9. PhyML phylogenetic relations and composition of domains for AT1G67030, AT3G58070, AT2G41940, AT5G06650, AT1G10480, AT1G68360 homologues of representative plant species. The color legends of species and domains is given below

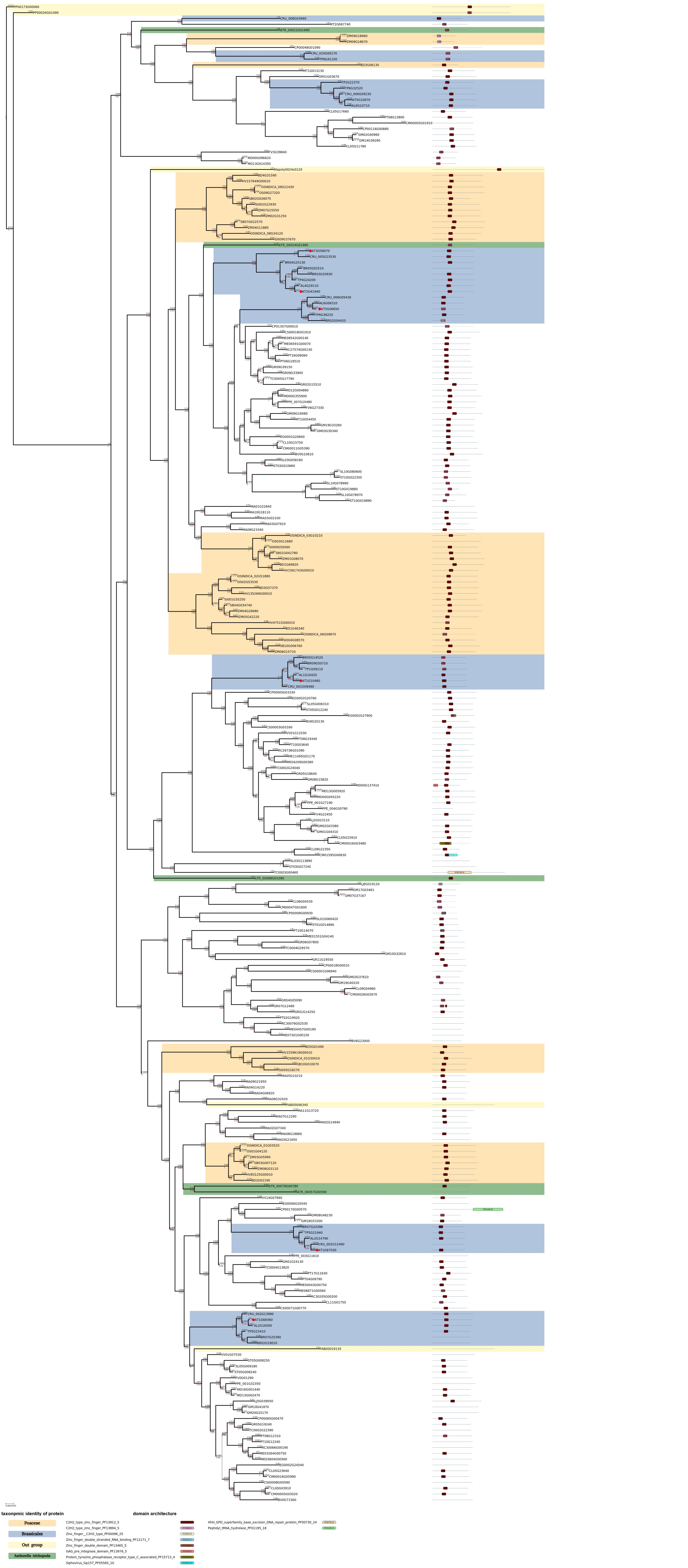

Figure S10. PhyML phylogenetic relations and composition of domains for AT1G13180 homologues of representative plant species. The color legends of species and domains is given below

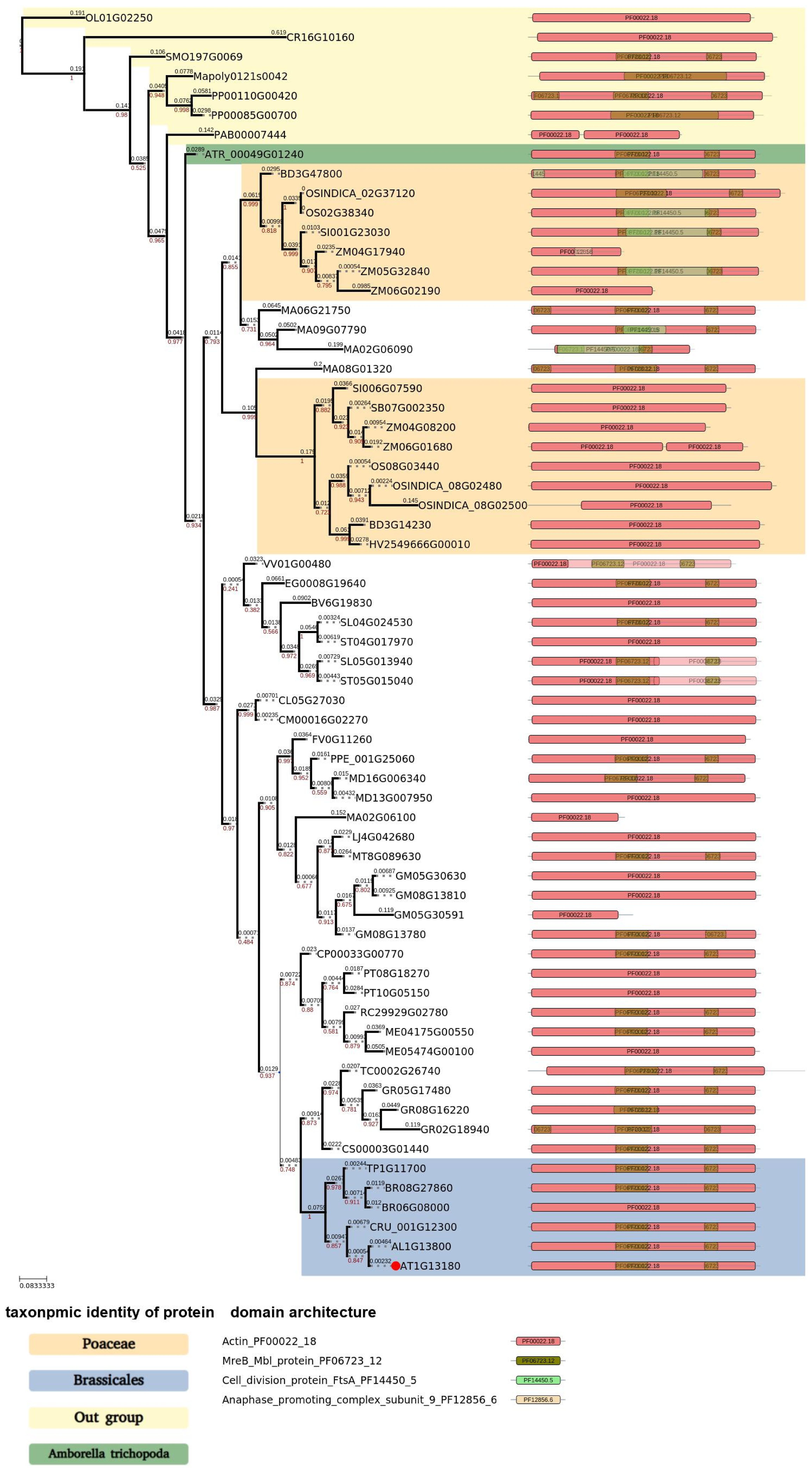

Figure S11. PhyML phylogenetic relations and composition of domains for AT1G66350, AT5G17490, AT3G03450, AT2G01570, AT1G14920 homologues of representative plant species. The color legends of species and domains is given below

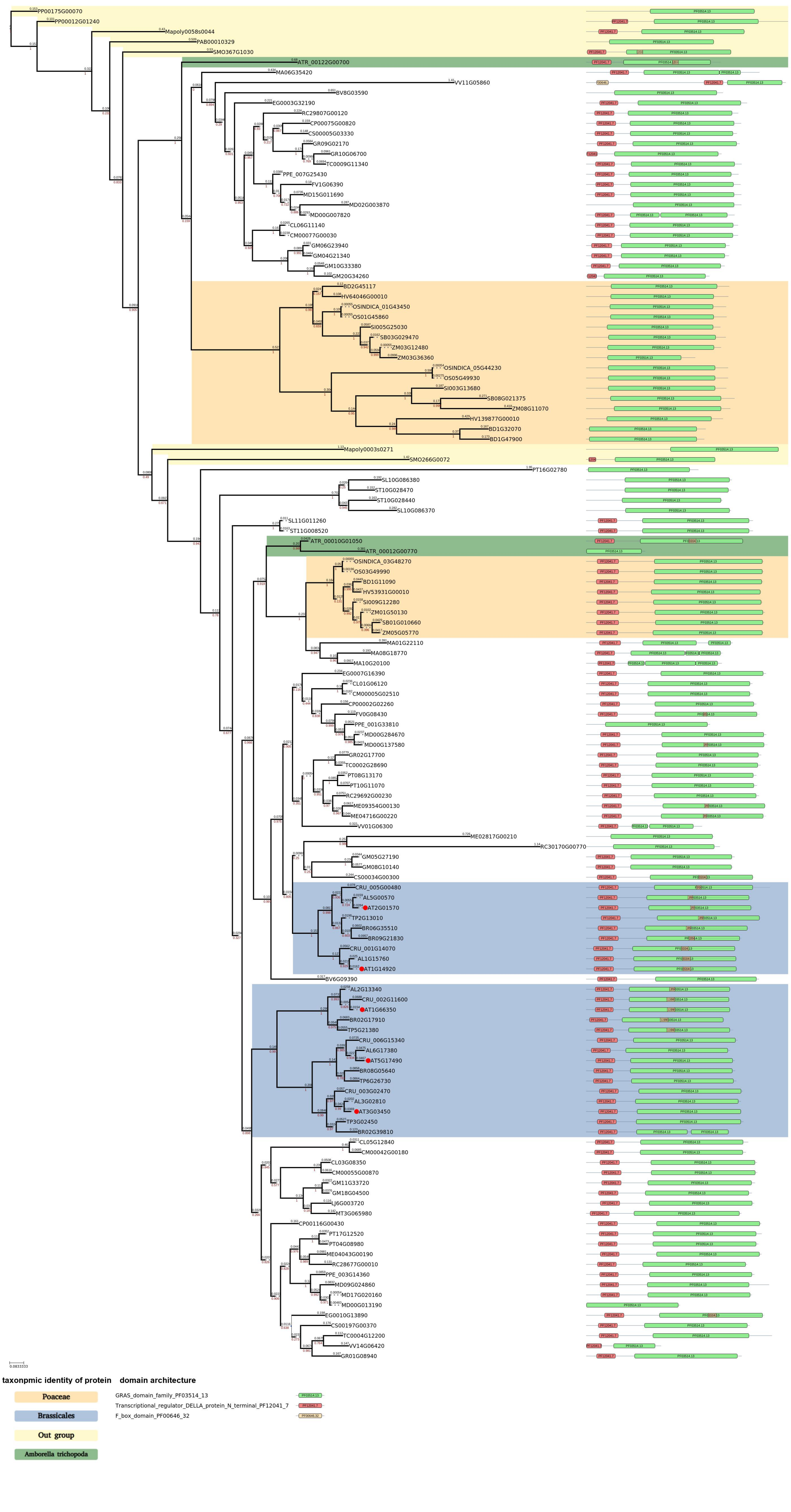

Figure S12. PhyML phylogenetic relations and composition of domains for AT1G17920, AT1G73360 homologues of representative plant species. The color legends of species and domains is given below

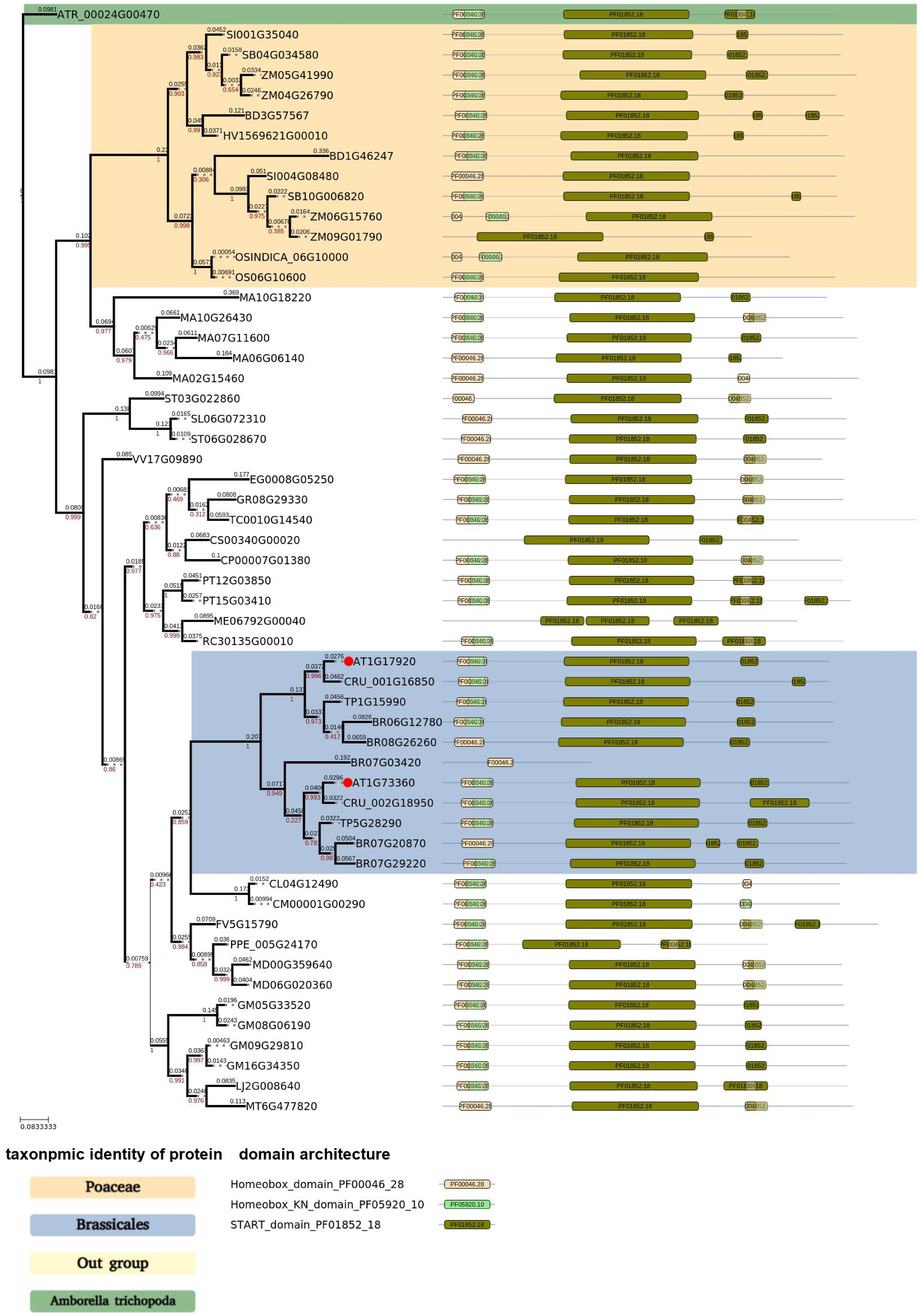

Figure S13. PhyML phylogenetic relations and composition of domains for AT1G19835 homologues of representative plant species. The color legends of species and domains is given below

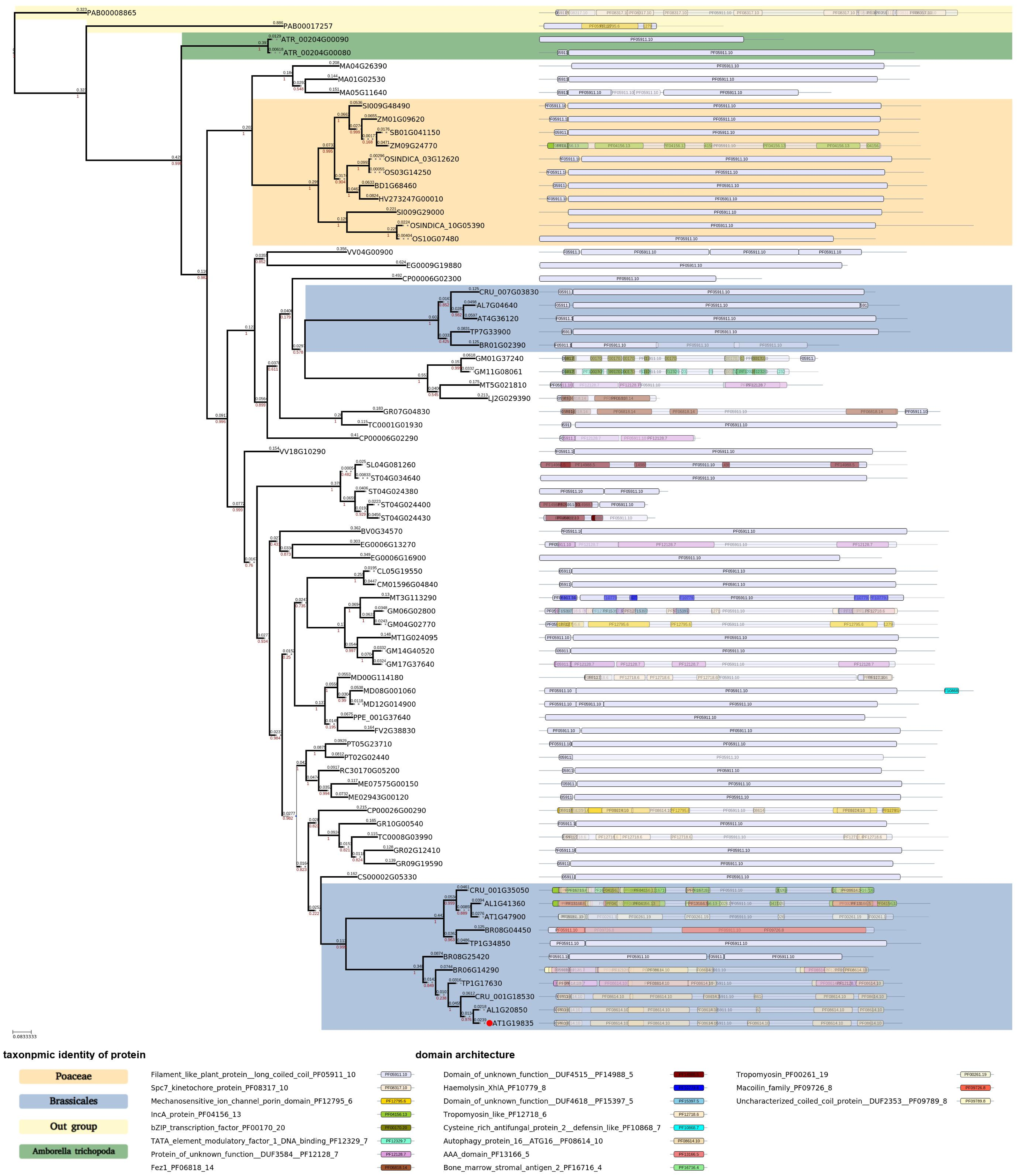

Figure S14. PhyML phylogenetic relations and composition of domains for AT1G25540 homologues of representative plant species. The color legends of species and domains is given below

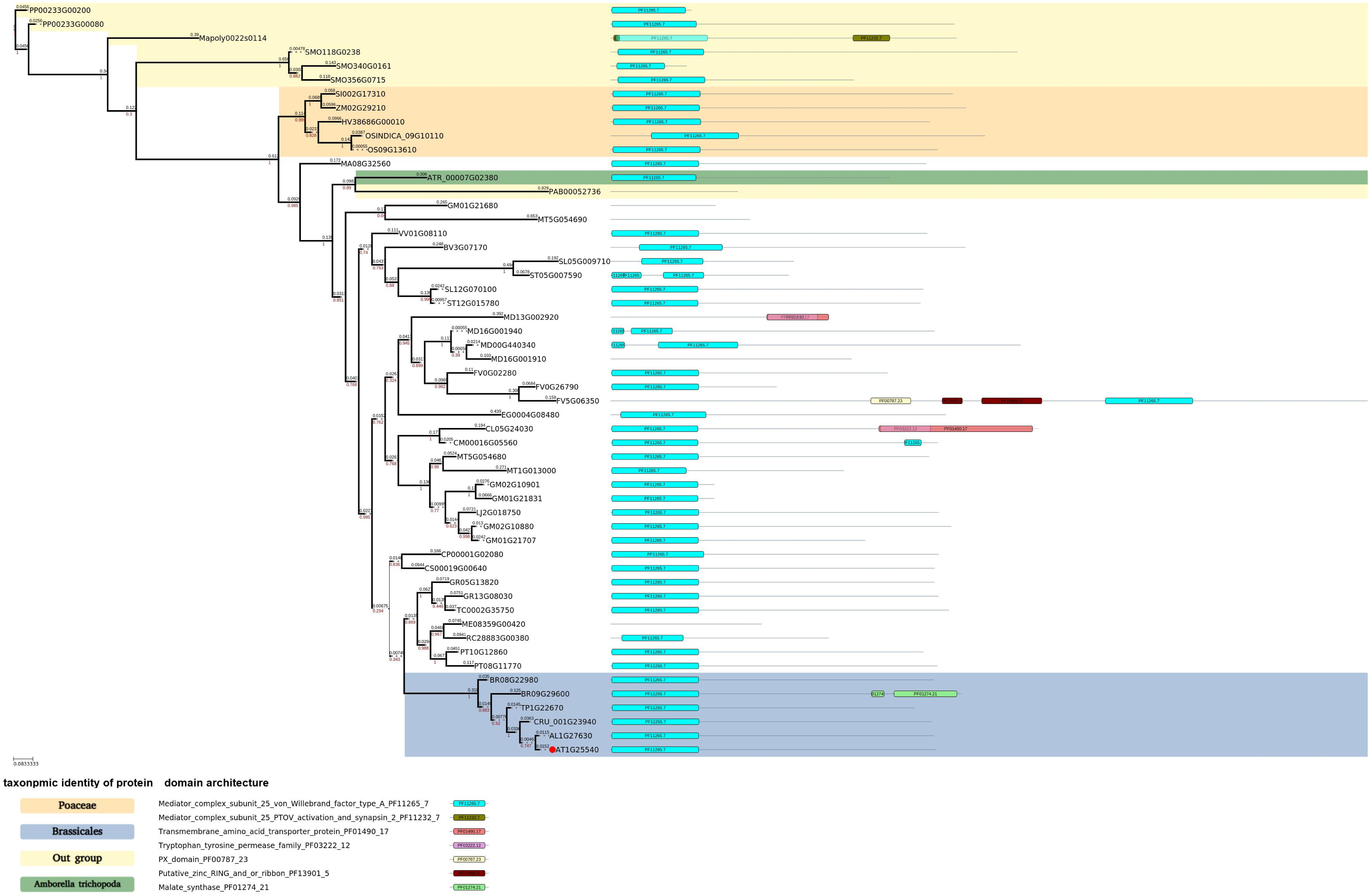

Supplement: Supplementary file 7 — Figure S5: PhyML phylogenetic relations and composition of domains for AT1G01510 homologues of representative plant species. Figure S6: PhyML phylogenetic relations and composition of domains for AT1G01520 homologues of representative plant species. Figure S7: PhyML phylogenetic relations and composition of domains for AT1G03060 homologues of representative plant species. Figure S8: PhyML phylogenetic relations and composition of domains for AT1G05230; AT4G21750; AT4G04890; AT1G79840 homologues of representative plant species. Figure S9: PhyML phylogenetic relations and composition of domains for AT1G67030; AT3G58070; AT2G41940; AT5G06650; AT1G10480; AT1G68360 homologues of representative plant species. Figure S10: PhyML phylogenetic relations and composition of domains for AT1G13180 homologues of representative plant species. Figure S11: PhyML phylogenetic relations and composition of domains for AT1G66350; AT5G17490; AT3G03450; AT2G01570; AT1G14920 homologues of representative plant species. Figure S12: PhyML phylogenetic relations and composition of domains for AT1G17920; AT1G73360 homologues of representative plant species. Figure S13: PhyML phylogenetic relations and composition of domains for AT1G19835 homologues of representative plant species. Figure S14: PhyML phylogenetic relations and composition of domains for AT1G25540 homologues of representative plant species. (PDF 10138 kb) [file 12870_2019_1640_MOESM7_ESM.pdf]
