# Supplementary material for: The evolution of gene regulatory networks controlling Arabidopsis thaliana L. trichome development
Source: BMC Plant Biol. 2019 Feb 15;19(Suppl 1):53. doi: 10.1186/s12870-019-1640-2 (PMC6393967; doi:10.1186/s12870-019-1640-2)

Figure S15. PhyML phylogenetic relations and composition of domains for AT1G29170, AT2G34150, AT2G38440 homologues of representative plant species. The color legends of species and domains is given below

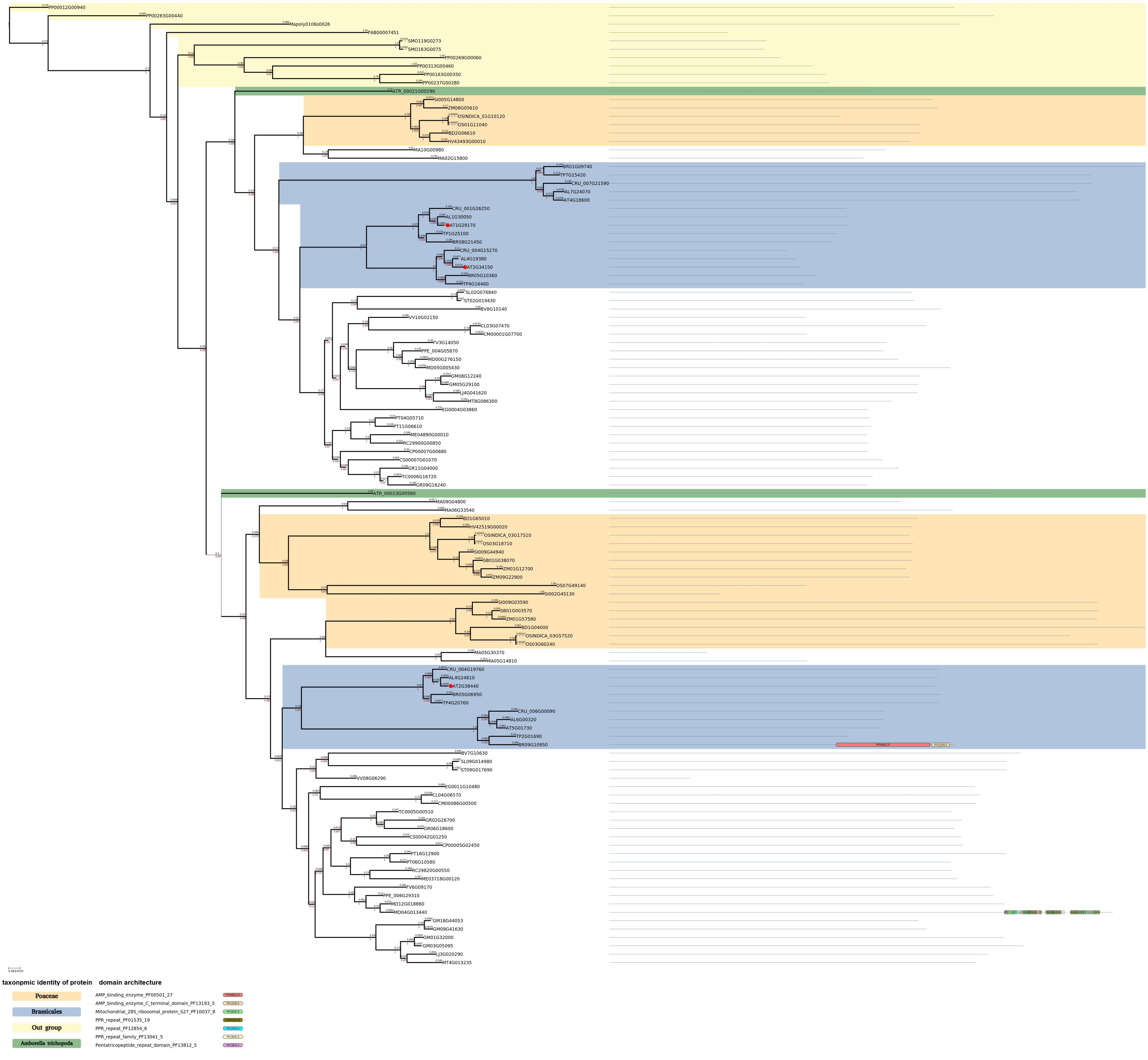

Figure S16. PhyML phylogenetic relations and composition of domains for AT2G33385, AT1G30825 homologues of representative plant species. The color legends of species and domains is given below

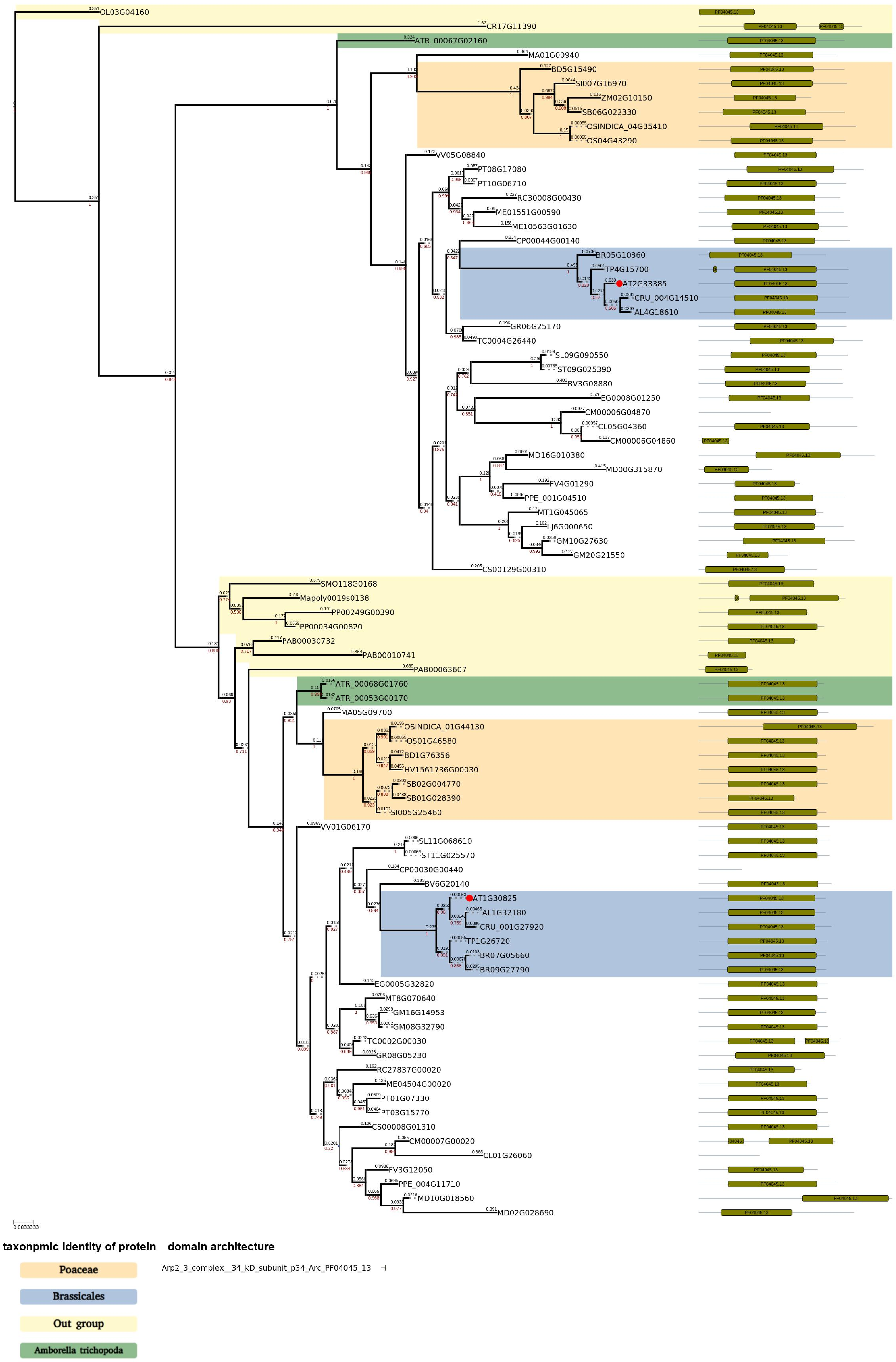

Figure S17. PhyML phylogenetic relations and composition of domains for AT1G33240 homologues of representative plant species. The color legends of species and domains is given below

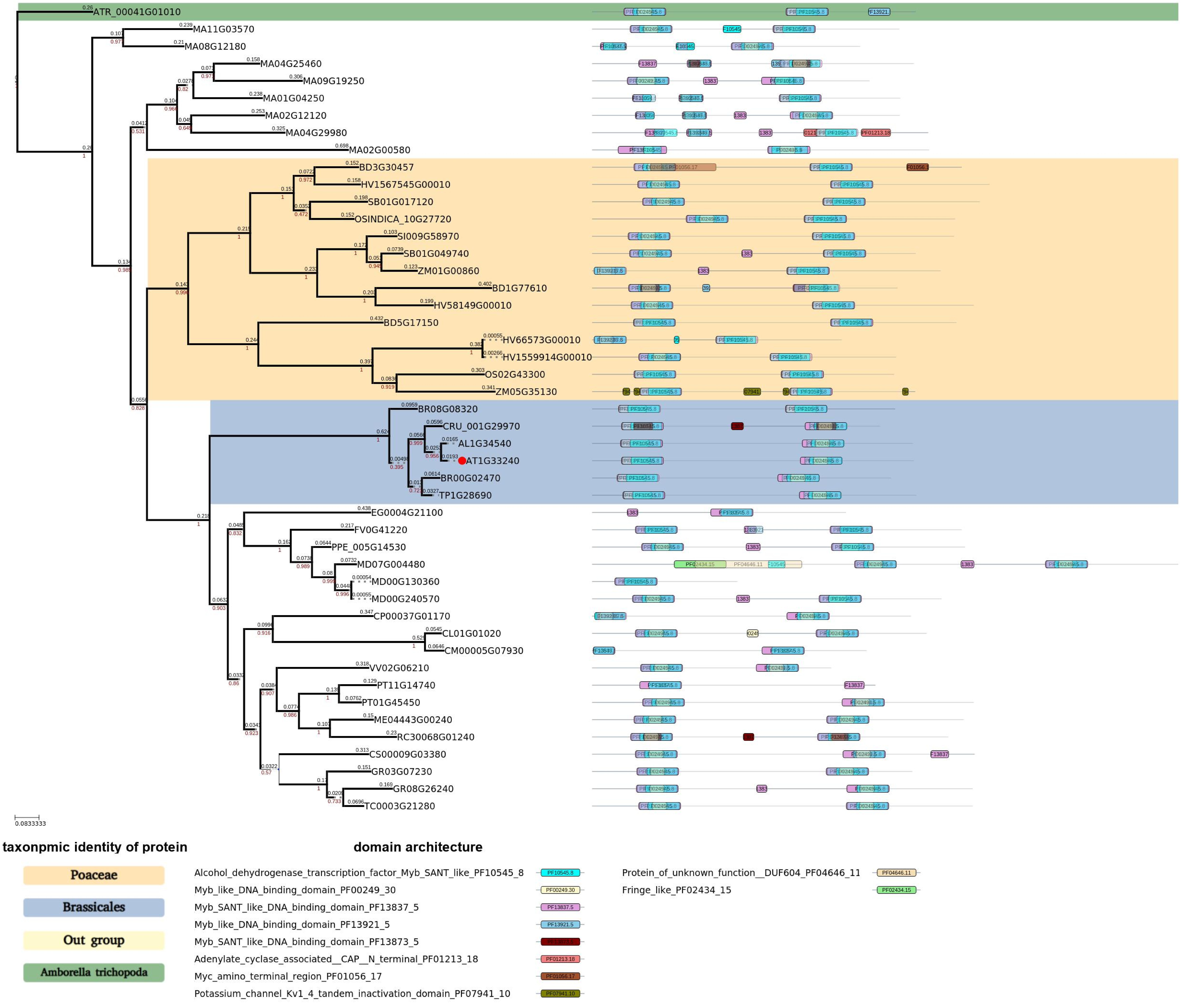

Figure S18. PhyML phylogenetic relations and composition of domains for AT1G60430 homologues of representative plant species. The color legends of species and domains is given below

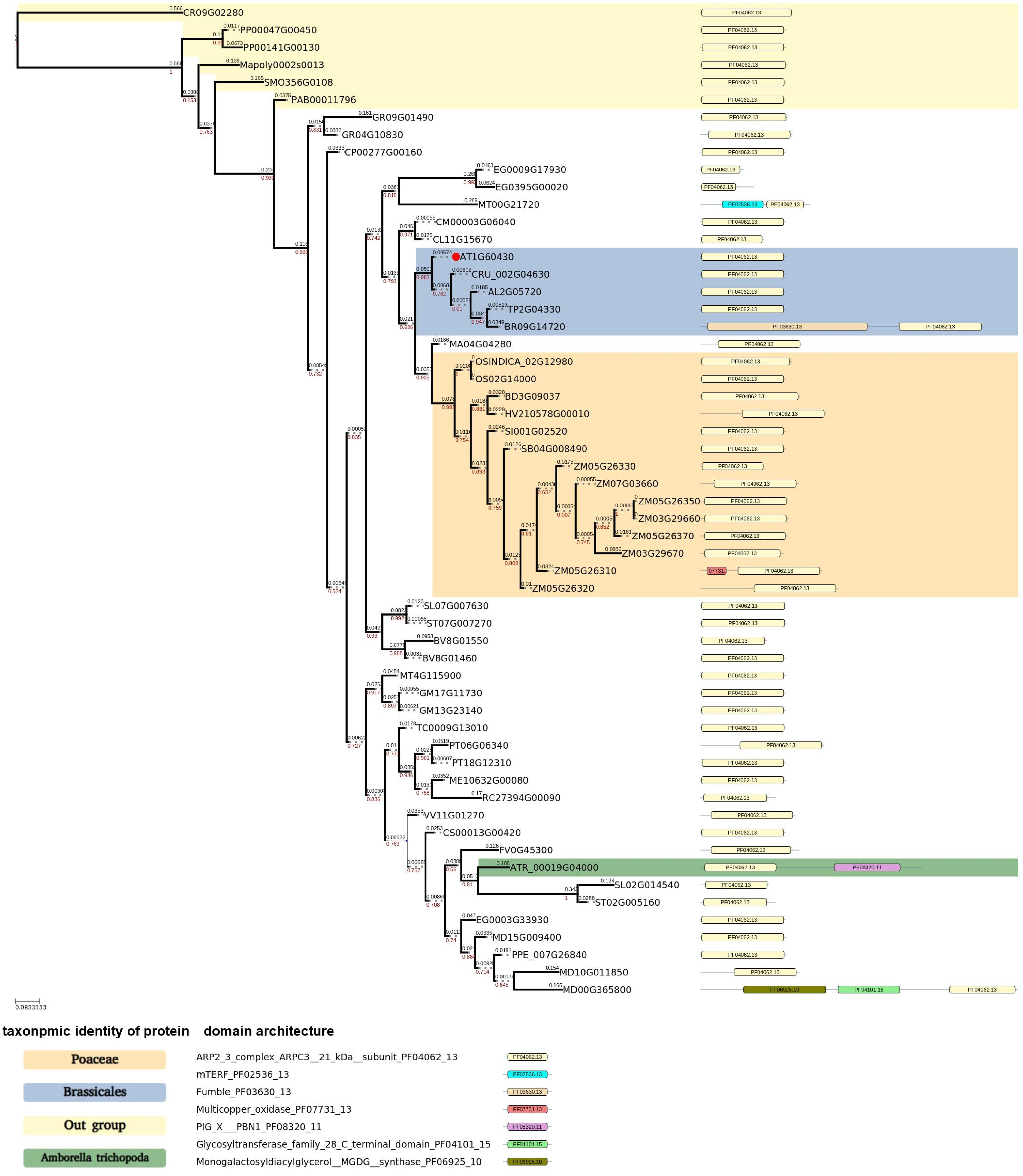

Figure S19. PhyML phylogenetic relations and composition of domains for AT1G64690 homologues of representative plant species. The color legends of species and domains is given below

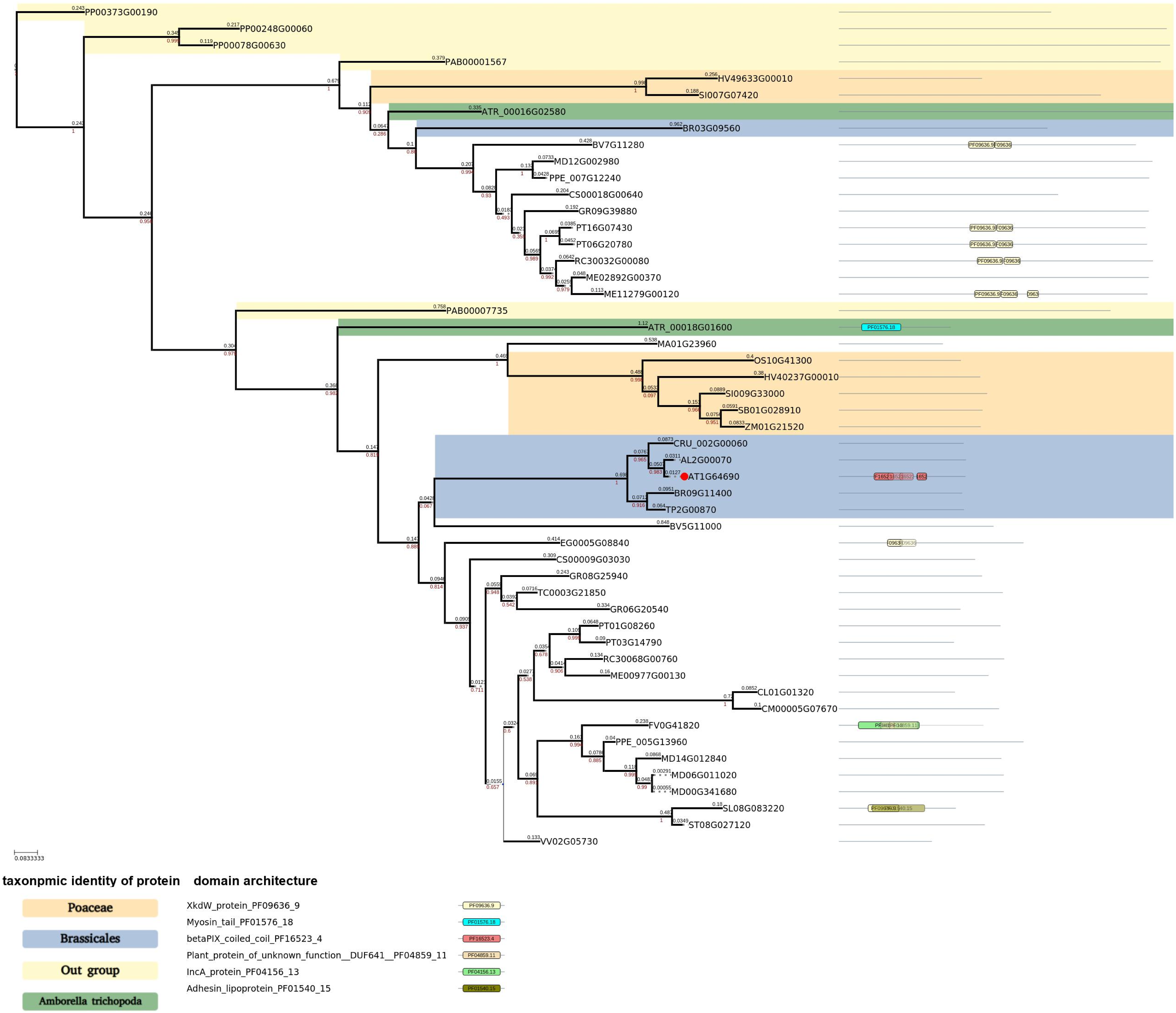

Figure S20. PhyML phylogenetic relations and composition of domains for AT1G65470 homologues of representative plant species. The color legends of species and domains is given below

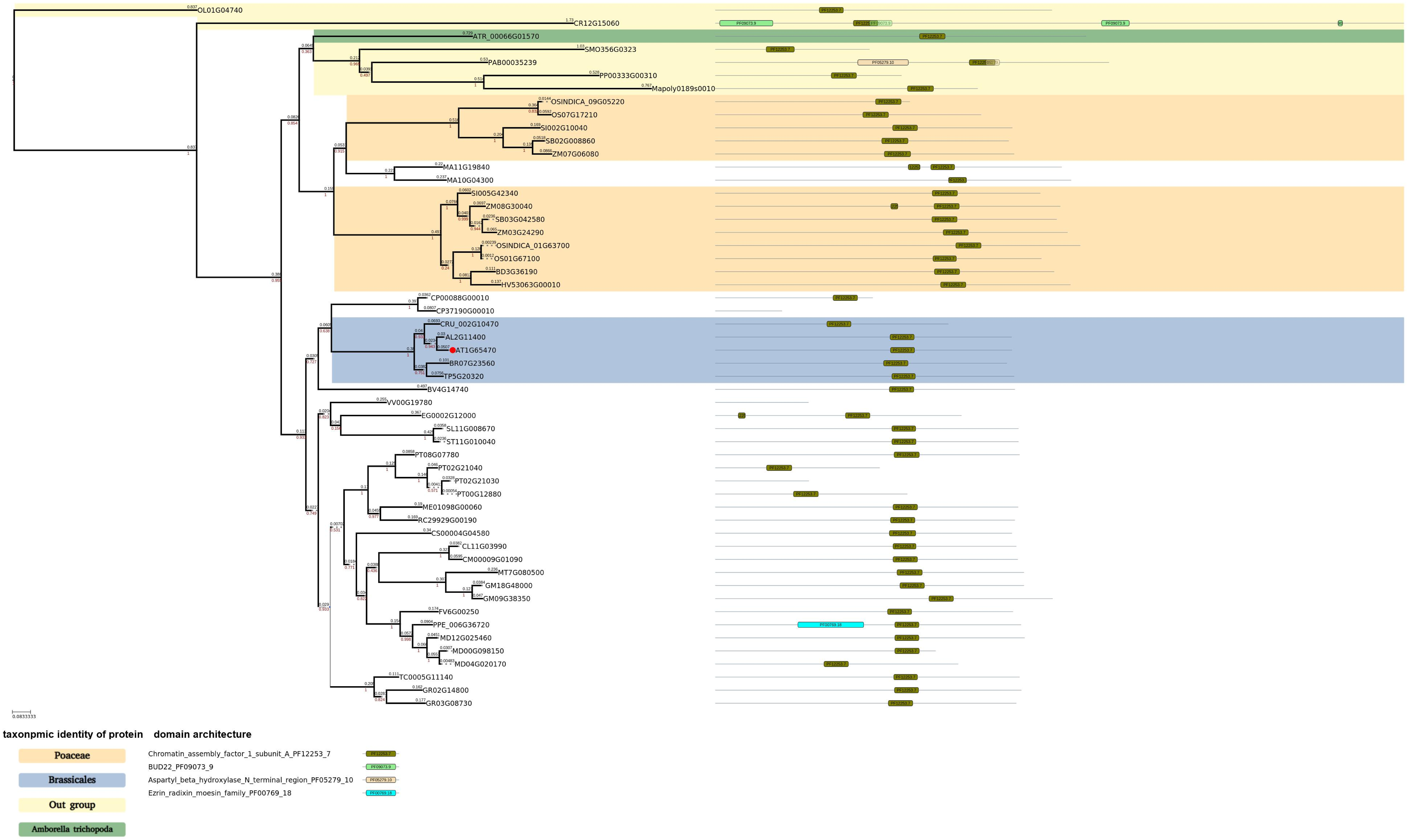

Figure S21. PhyML phylogenetic relations and composition of domains for AT5G38110, AT1G66740 homologues of representative plant species. The color legends of species and domains is given below

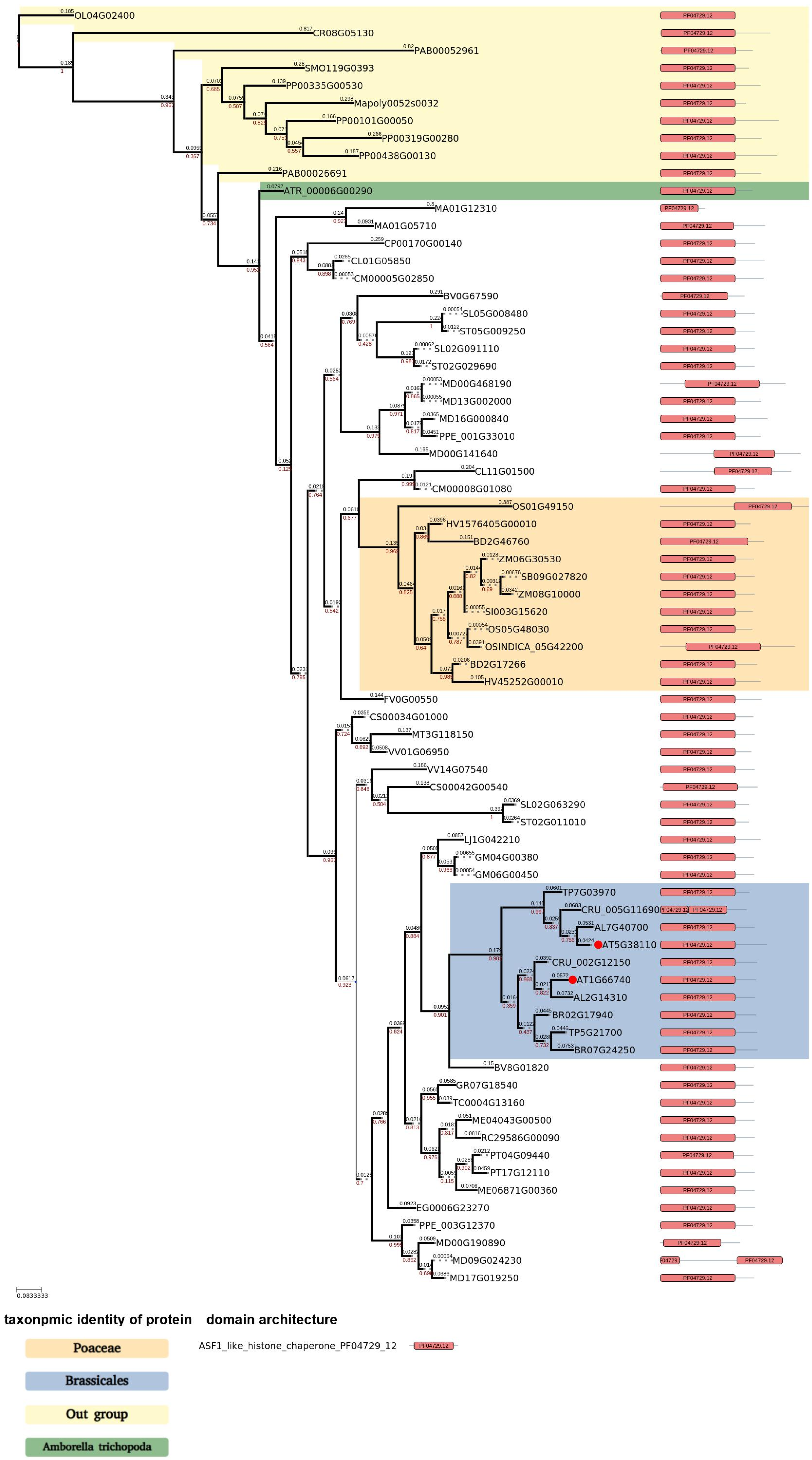

Figure S22. PhyML phylogenetic relations and composition of domains for AT1G69490 homologues of representative plant species. The color legends of species and domains is given below

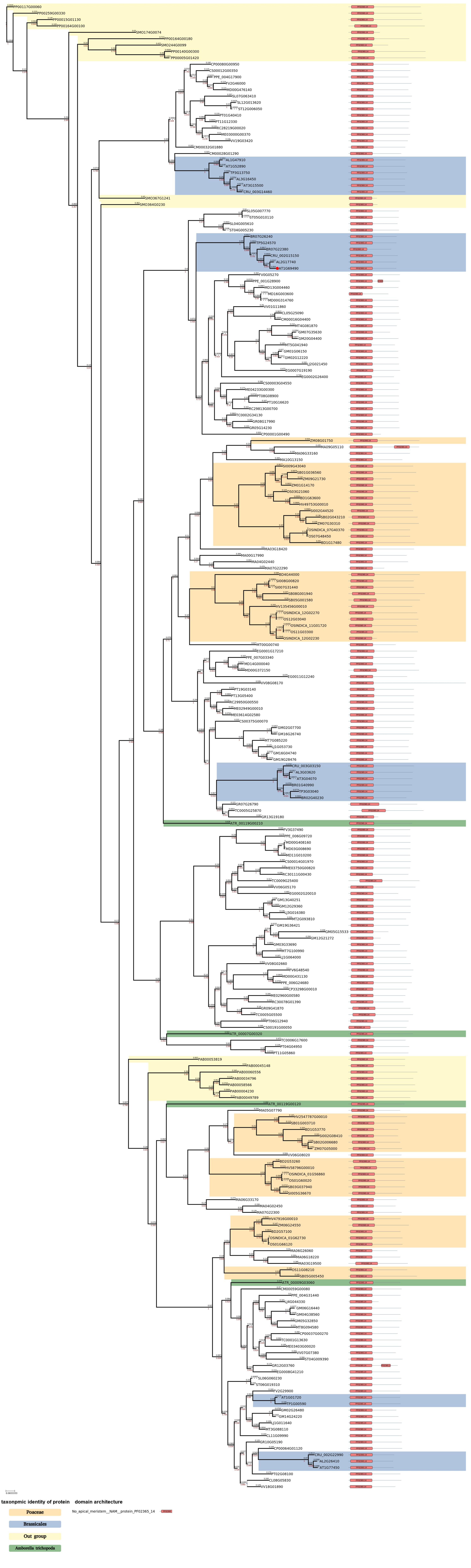

Figure S23. PhyML phylogenetic relations and composition of domains for AT1G75950 homologues of representative plant species. The color legends of species and domains is given below

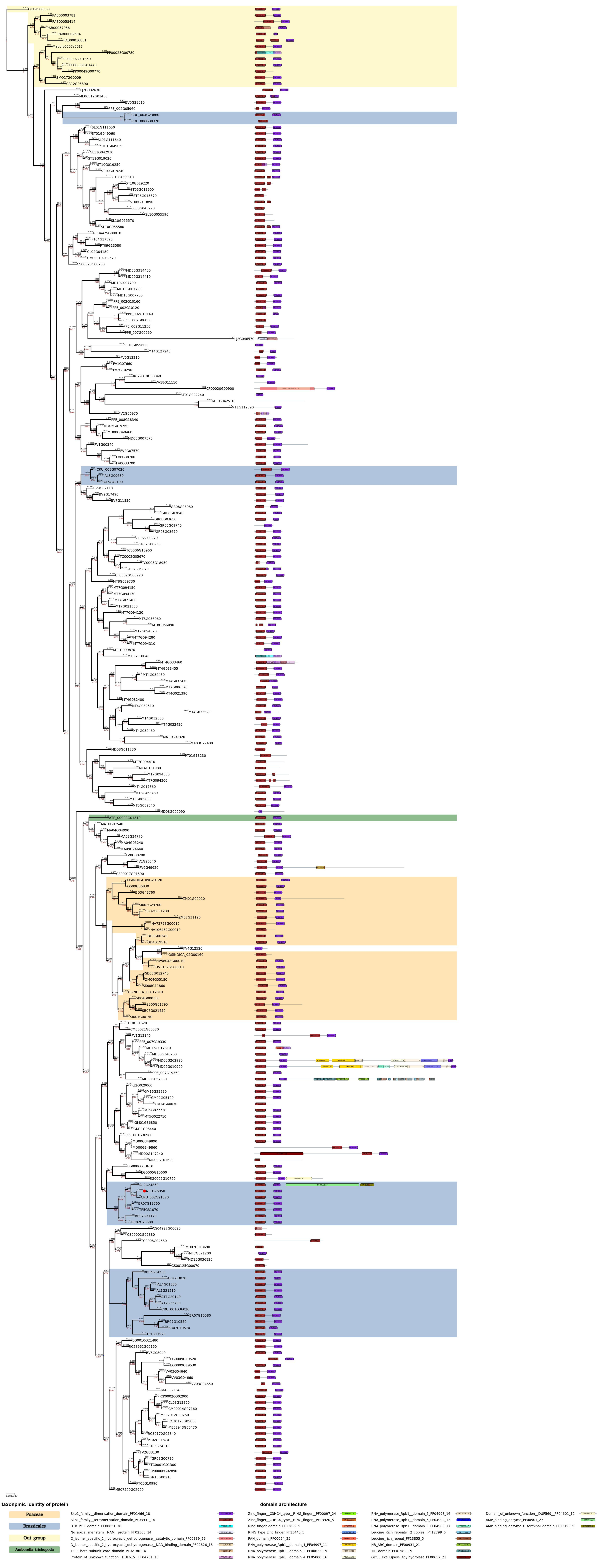

Figure S24. PhyML phylogenetic relations and composition of domains for AT1G80350 homologues of representative plant species. The color legends of species and domains is given below

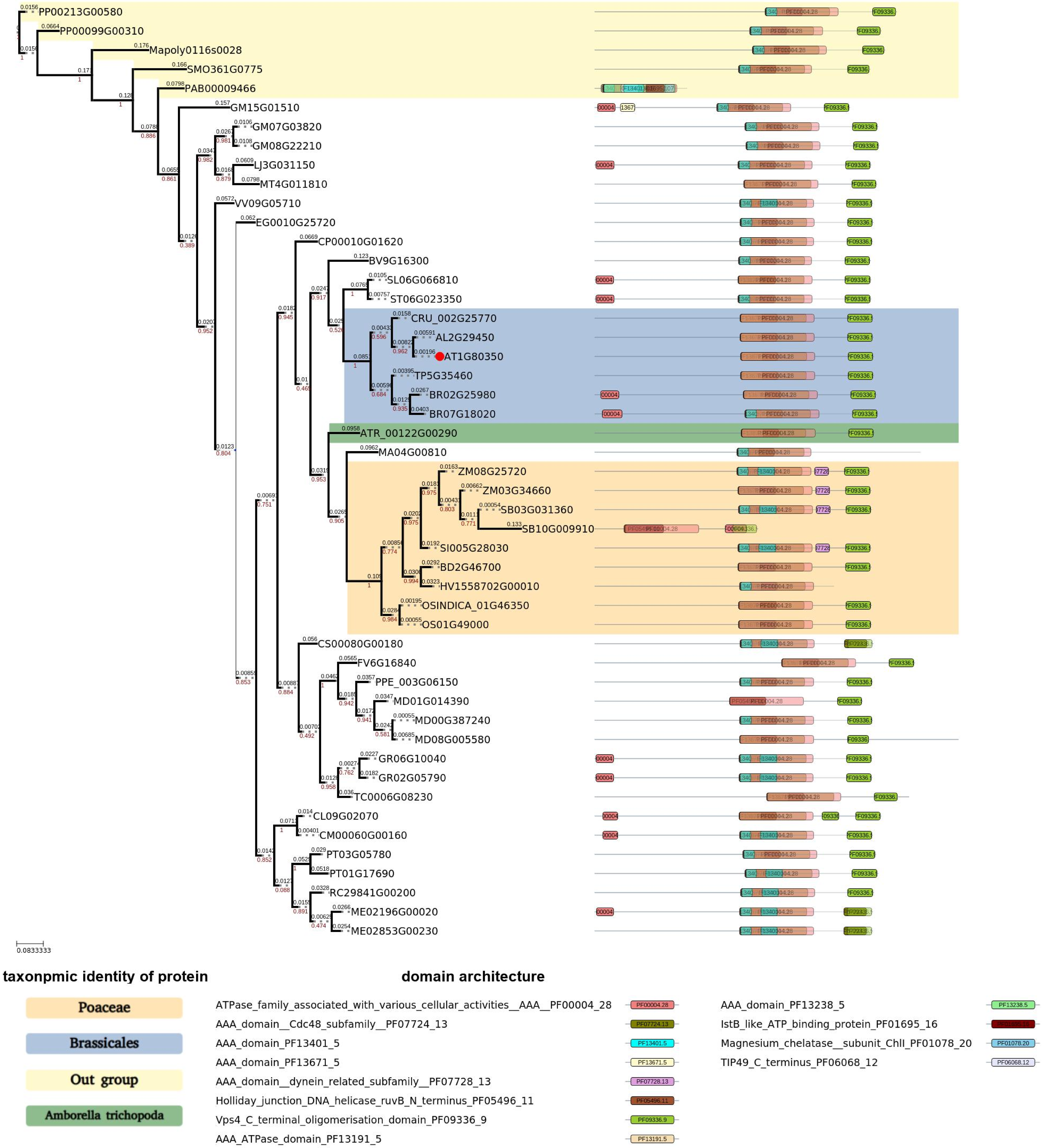

Supplement: Supplementary file 8 — Figure S15: PhyML phylogenetic relations and composition of domains for AT1G29170; AT2G34150; AT2G38440 homologues of representative plant species. Figure S16: PhyML phylogenetic relations and composition of domains for AT2G33385; AT1G30825 homologues of representative plant species. Figure S17: PhyML phylogenetic relations and composition of domains for AT1G33240 homologues of representative plant species. Figure S18: PhyML phylogenetic relations and composition of domains for AT1G60430 homologues of representative plant species. Figure S19: PhyML phylogenetic relations and composition of domains for AT1G64690 homologues of representative plant species. Figure S20: PhyML phylogenetic relations and composition of domains for AT1G65470 homologues of representative plant species. Figure S21: PhyML phylogenetic relations and composition of domains for AT5G38110; AT1G66740 homologues of representative plant species. Figure S22: PhyML phylogenetic relations and composition of domains for AT1G69490 homologues of representative plant species. Figure S23: PhyML phylogenetic relations and composition of domains for AT1G75950 homologues of representative plant species. Figure S24: PhyML phylogenetic relations and composition of domains for AT1G80350 homologues of representative plant species. (PDF 7989 kb) [file 12870_2019_1640_MOESM8_ESM.pdf]
